# Enantioselective electrophilic α-fluorination catalyzed by an artificial metalloenzyme

**DOI:** 10.1039/d6sc00858e

**Published:** 2026-03-24

**Authors:** Jinmeng Yu, Chang Wang, Wenhao Hu, Huan Wang, Jing Zhao, Hui-Jie Pan

**Affiliations:** a State Key Laboratory of Coordination Chemistry, Chemistry and Biomedicine Innovation Center (ChemBIC), ChemBioMed Interdisciplinary Research Center at Nanjing University, School of Chemistry and Chemical Engineering, Nanjing University Nanjing 210023 P. R. China huijie.pan@nju.edu.cn

## Abstract

Fluorine incorporation profoundly influences the properties of pharmaceuticals and imaging agents, yet enzymatic C–F bond formation remains exceedingly rare. Here we report an enantioselective electrophilic α-fluorination catalyzed by an artificial metalloenzyme assembled from a biotinylated Cu(ii) Lewis acid cofactor embedded in streptavidin. Targeted mutagenesis of residues S112 and K121 yielded variants delivering up to 95% ee. Substrate scope studies revealed distinct steric and electronic influences on both reactivity and selectivity. Docking and molecular dynamics simulations indicate that precise cofactor positioning and steric shielding from K121Q govern the approach of the electrophilic fluorinating reagent, accounting for the observed enantioselectivity. These findings demonstrate that electrophilic fluorination chemistry can be engineered into protein environments and highlight the broader potential of artificial metalloenzymes to enable new-to-nature biotransformations.

## Abstract 2

Enantioselective electrophilic α-fluorination was achieved using a Sav–biotin-based artificial metalloenzyme with moderate to high yields and enantioselectivities.

## Graphical abstract

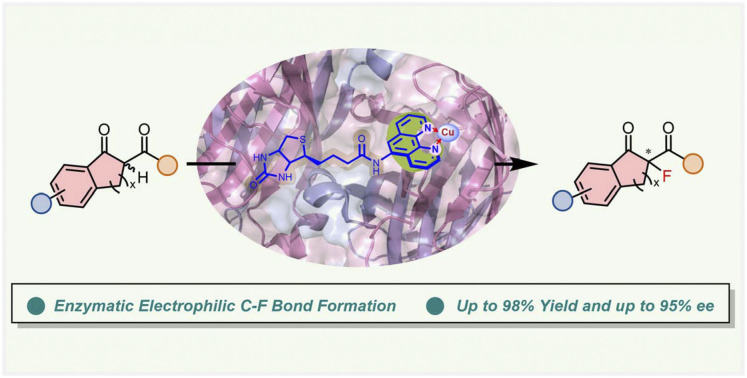

## Introduction

Fluorine-containing compounds play a pivotal role in modern chemistry, medicine, and molecular imaging. The incorporation of fluorine atoms or fluorinated motifs can profoundly modulate molecular properties, improving metabolic stability, bioavailability, and target selectivity.^1,2^ Consequently, more than one quarter of approved pharmaceuticals contain at least one C–F bond.^3–5^ Moreover, fluorine isotopes extend these applications even further: ^18^F is essential for positron emission tomography (PET),^6^ and ^19^F provides a powerful handle for magnetic resonance imaging (MRI).^7^ These wide-ranging applications underscore the continuing demand for efficient and selective catalytic strategies for C–F bond formation.

**Fig. 1:**
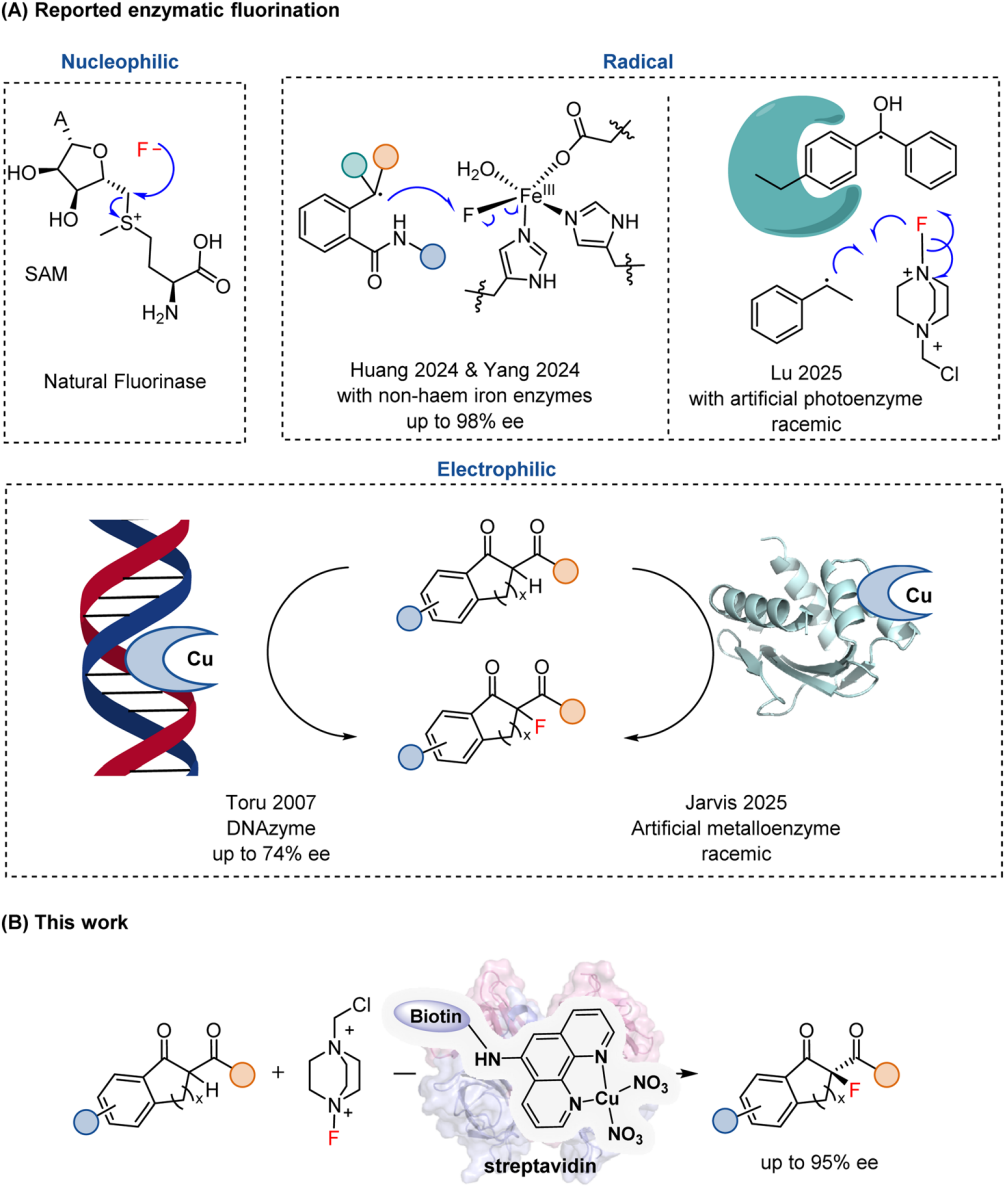
Enzymatic fluorination. (A) Reported enzymatic fluorinations with three distinct mechanisms; (B) enzymatic enantioselective electrophilic fluorination catalyzed by artificial metalloenzyme based on streptavidin–biotin technology.

In synthetic chemistry, diverse approaches^8–14^ have been established for constructing C–F bonds, including electrophilic, nucleophilic, and radical fluorination. By contrast, enzymatic C–F bond formation remains exceedingly rare.^15–17^ To date, only a single natural fluorinase has been identified, which catalyzes the nucleophilic substitution of *S*-adenosylmethionine (SAM) with fluoride to yield 5′-fluorodeoxyadenosine (5′-FDA) (Fig. 1A).^18,19^ In addition, engineered glycosyltransferases have been shown to generate glycosyl fluorides through fluoride displacement reactions.^20^

The growing demand for sustainable chemical synthesis and the rapid expansion of the bioeconomy call for biocatalytic systems capable of executing transformations beyond nature's repertoire. Developing fluorinating enzymes that operate through alternative mechanisms would therefore greatly expand the scope of biocatalysis. Along this line, non-heme iron enzymes have recently been repurposed by the Huang^21^ and Yang^22^ groups to mediate enantioselective intramolecular radical relay fluorination (Fig. 1A), while the Lu^23^ group reported a *de novo* photoenzyme enabling intermolecular radical fluorination, albeit in a racemic manner (Fig. 1A).

In sharp contrast to the rapid progress in small-molecule catalysis, enzymatic C–F bond formation *via* an electrophilic mechanism remains particularly challenging. A DNA-based catalyst (DNAzyme) was reported to promote enantioselective electrophilic fluorination of β-ketoesters with enantiomeric excesses of up to 74% (Fig. 1A),^24^ demonstrating that chiral macromolecular scaffolds can impart stereocontrol over this transformation. In parallel, protein-based artificial metalloenzymes derived from sterol carrier protein 2-like (SCP2L) scaffolds have recently been shown to catalyze electrophilic fluorination; however, these systems afforded only racemic products (Fig. 1A).^25^ Collectively, these studies highlight that achieving highly enantioselective electrophilic fluorination within a protein-based catalytic framework remains an unmet challenge.

Encouraged by the success of chiral Lewis acid catalysis^26–31^ in enantioselective electrophilic fluorination and by recent advances in artificial metalloenzymes (ArMs) that introduce abiological reactivities into protein scaffolds,^32–48^ we sought to construct a protein-based ArM capable of promoting enantioselective electrophilic C–F bond formation. Among available ArM platforms, the streptavidin–biotin (Sav–biotin) system offers exceptional versatility, owing to its ultrahigh binding affinity and its ability to accommodate diverse metal cofactors (Rh,^49^ Ir,^50^ Cu,^51^ Fe,^52,53^ Ru^54^) as well as reactive intermediates such as radicals,^52^ anions,^55^ and cations.^56^ Sav-biotin-based Lewis acidases have previously enabled reactions including conjugate additions,^57,58^ Mannich reaction^59^ and Diels–Alder cycloadditions,^60^ underscoring their capacity to stabilize multiple intermediates and control stereochemical outcomes within a protein environment.

Building upon these advances, we developed a Sav–biotin-based artificial metalloenzyme that promotes Lewis acid-mediated electrophilic fluorination with high enantioselectivity (up to 95% ee, Fig. 1B). Benefiting from facile and robust assembly, together with the ready evolvability of both the metallocofactor and the protein scaffold, this system provides a highly modular and tunable platform for asymmetric fluorination.

## Results

For a direct comparison with precedent examples,^24,25^ the α-fluorination of 1a with electrophilic fluorinating reagent Selectfluor (2) was selected as the model reaction. In the absence of a catalyst, a bit of background reactivity was observed (Table 1, Entry 1). The addition of either the Lewis acid cofactor or the Sav protein alone enhanced product formation, indicating the catalytic activity of the cofactor and a beneficial effect of the protein scaffold (Table 1, Entries 2 and 3). In the presence of a holo artificial metalloenzyme assembled from cofactor 1 or cofactor 2 with wild-type Sav, enantioselectivity were obtained (Table 1, Entries 4 and 5). Among them, cofactor 1 gave better performance.

**Table 1:** Model reaction development and reaction condition optimizations

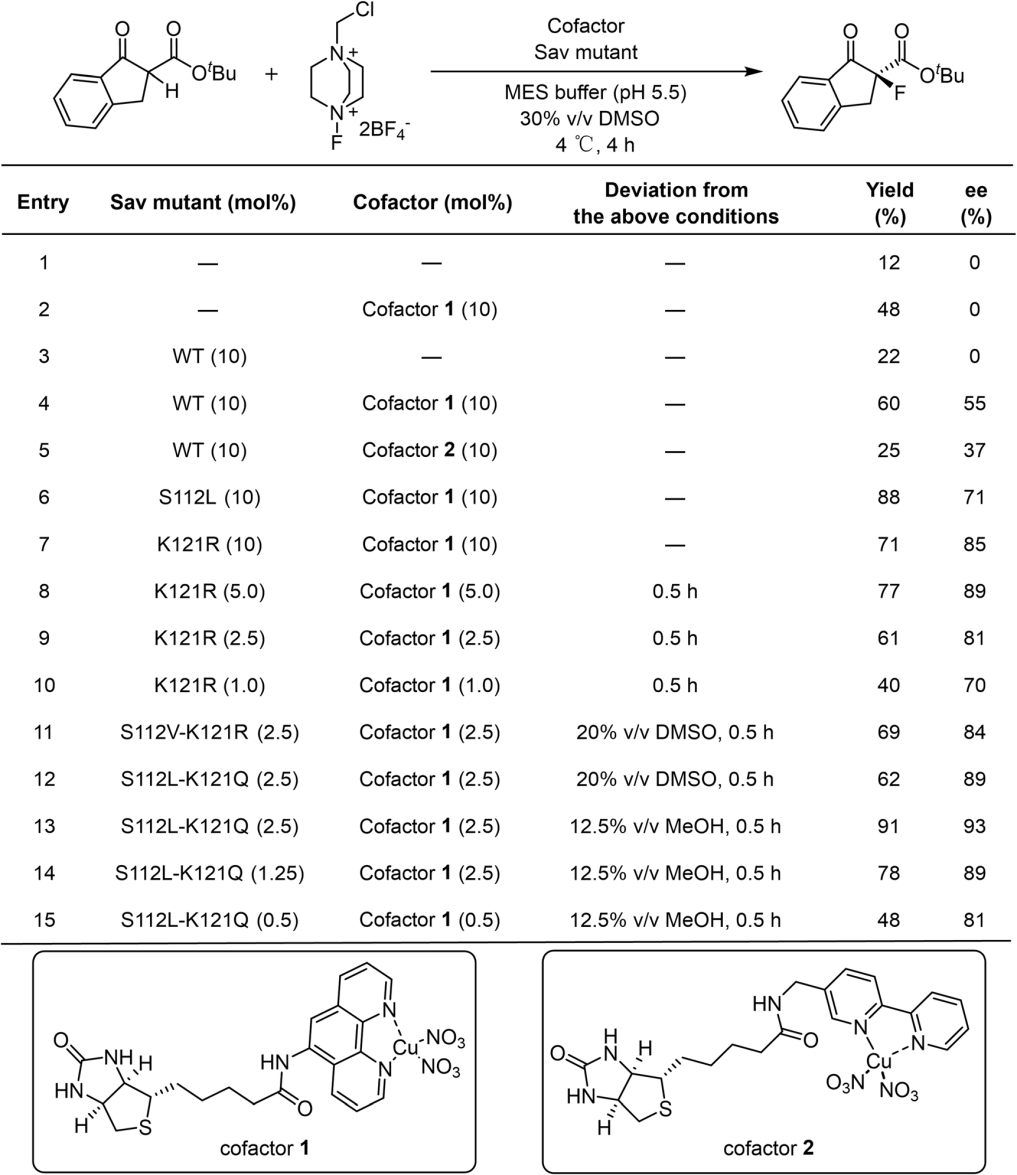

**Fig. 2:**
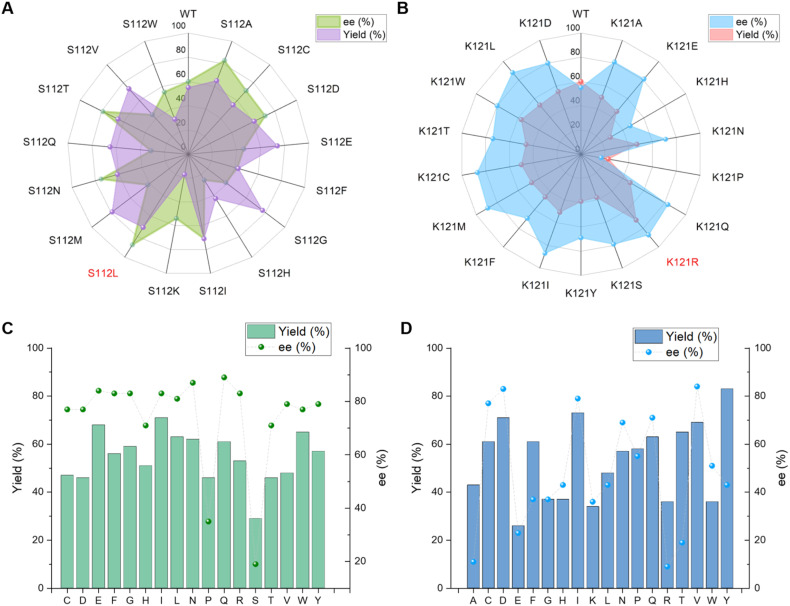
Screening of Sav mutants. (A) Screening of Sav mutants at S112 position; (B) screening of Sav mutants at K121 position; (C) screening of Sav double mutants of S112L–K121X; (D) screening of Sav double mutants of S112X–K121R.

We next screened Sav mutants focusing on residues S112 and K121, which are close to the metal center (Fig. 4B) and are reported to critically influence the reactivity and enantioselectivity of Sav–biotin system^49,53,58,61^ (Fig. 2A, B and Table S2). Two variants, S112L and K121R, showed markedly improved performance, affording product 3a in 88% yield with 71% ee, and 71% yield with 85% ee, respectively (Table 1, Entries 6 and 7). With these mutants, the catalyst loading could be reduced to 2.5 mol% and the reaction time shortened to 0.5 h without compromising yield or selectivity (Tables 1, Entry 9 and S3 and S4). Further exploration of double mutants revealed limited improvement (Fig. 2C, D and Table S5). Introducing S112V into K121R slightly increased the ee from 81% to 84%, while combining K121Q with S112L improved the ee to 89%. Subsequent optimization of the reaction conditions using the S112L–K121Q mutant revealed that using methanol as cosolvent was beneficial, affording 3a in 91% yield and 93% ee (Tables 1, Entry 13; S6 and S7). The sav/cofactor ratio was crucial. Changing the tetramer Sav/cofactor ratio from 1 : 1 to 1 : 2 leaded to a moderate drop in yield and slight drop in enantioselectivity (Table 1, Entry 14). Under lower catalyst loading (0.5 mol%), the reaction still worked, although with a lower yield of 48% and a diminished ee of 81% (Table 1, Entry 15).

**Fig. 3:**
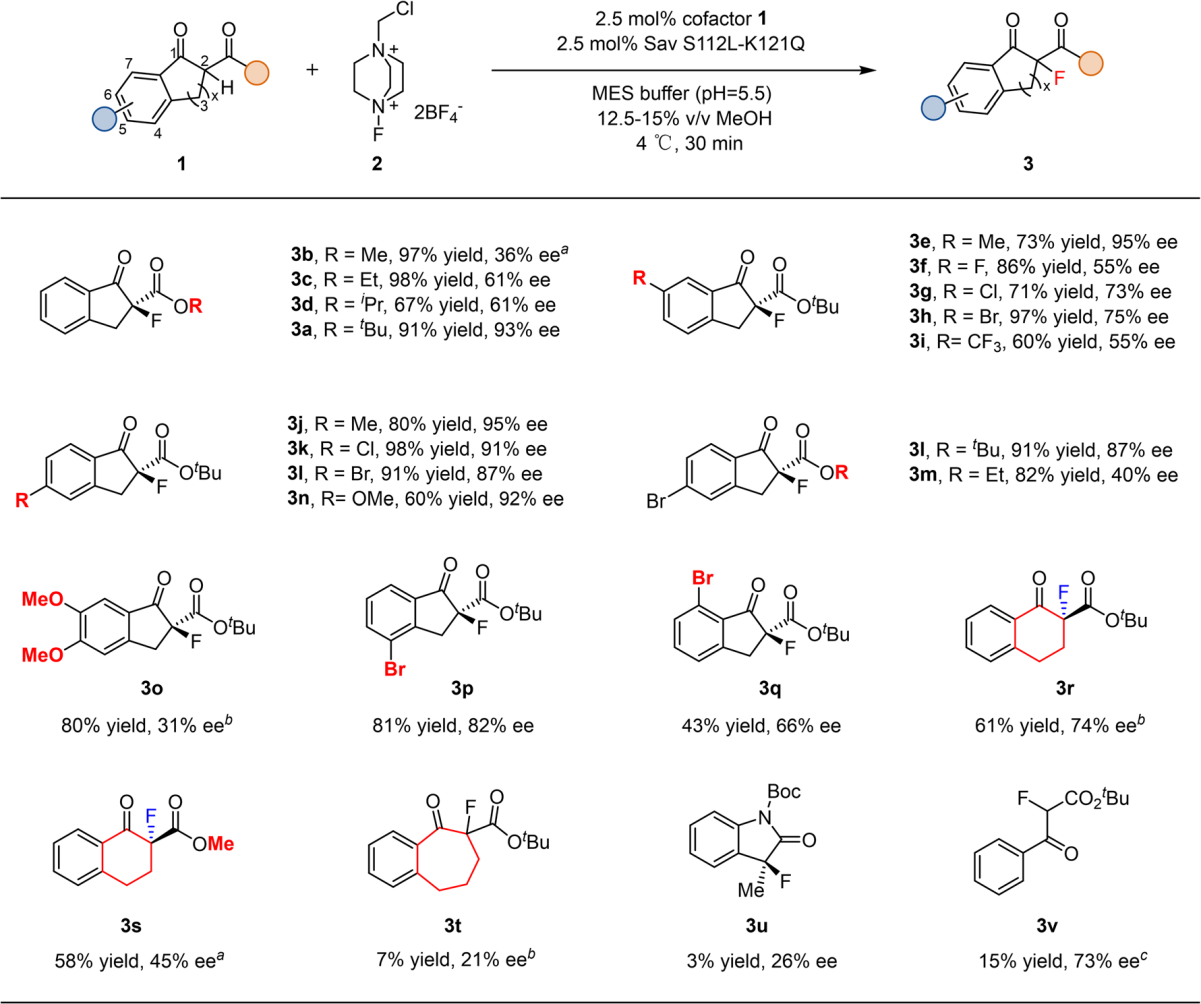
Substrate scope investigation. The absolute configuration of the product was assigned by comparing the HPLC trace with literature.^28,30,62–65^^*a*^ Sav S112L–K121N was used. ^*b*^ Sav S112I–K121R was used. ^*c*^ Sav K121R was used.

With the optimized system, we examined the substrate scope of this transformation (Fig. 3). The steric bulk of the ester group in 1 had a pronounced effect on enantioselectivity. Substrate 1b bearing a small methyl ester gave only 36% ee, whereas increasing the steric demand gradually enhanced the selectivity (3a–3d). Substituents at the 6-position of the aromatic ring also influenced enantioselectivity. Neutral groups such as methyl were well tolerated, while electron-withdrawing substituents significantly decreased the ee, following a trend where stronger withdrawing effects led to lower enantioselectivity (3e–3i). In contrast, substitutions at the 5-position were generally well accommodated, and variations in electronic properties (3j–3n) had little impact on either yield or ee. A 5,6-dimethoxy-substituted substrate (3o) exhibited poor reactivity, likely due to steric congestion. The reaction was more sensitive to substituents at the 7-position than at the 4-position (3p*vs.*3q). Expanding the cyclic ketone from a five-membered indanone to a six-membered tetralone significantly reduced both yield and enantioselectivity (3r and 3s), while further expansion to a seven-membered ring led to only 7% yield and 21% ee (3t). An indole-derived substrate was also compatible with this catalytic system, albeit affording the product (3u) with diminished yield and enantioselectivity. A non-cyclic substrate could also be converted in 15% yield and 73% ee (3v). These results indicate that substrates deviating substantially from the model scaffold are less efficiently accommodated within the enzyme pocket, and further protein evolution will be required to expand the substrate scope. Overall, compared with the DNAzyme approach,^24^ our system shows higher catalytic efficiency (2.5 mol% *versus* 30 mol% enzyme loading), improved enantioselectivity (up to 95% ee *versus* 74% ee), and a broader substrate scope.

**Fig. 4:**
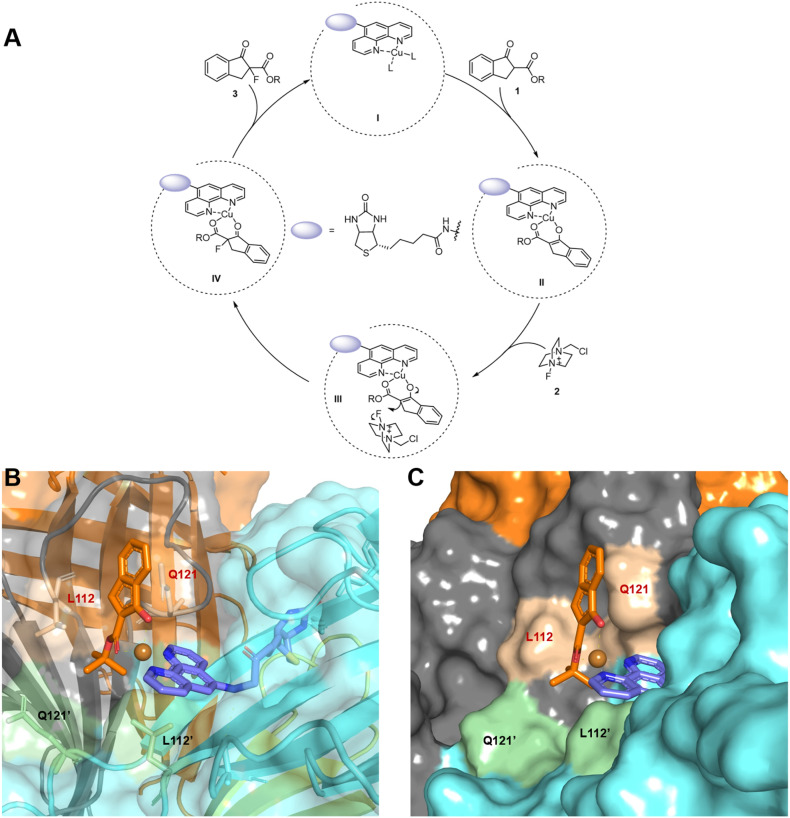
Mechanistic study. (A) Proposed catalytic cycle; (B) intermediate II obtained from docking and MD using the crystal structure of a reported streptavidin mutant^66^ (PDB: 5K67, mutations at S112 and K121 were introduced *in silico*); (C) illustration of the steric effect by Q121.

Previous Lewis acid-mediated fluorination reactions employing reagent 2 are generally proposed to proceed *via* an electrophilic pathway.^26–31^ Consistent with this mechanism, radical trapping experiments revealed that the addition of TEMPO had no discernible effect on the reaction outcome, either in the presence or absence of the Sav protein, thereby excluding the involvement of radical intermediates (Fig. S8). Therefore, we propose a catalytic cycle depicted in Fig. 4A. In the enzyme pocket, substrate 1 first coordinates to the Cu(ii) center through its two carbonyl oxygens, displacing the original NO_3_^−^ or H_2_O ligands. Subsequent deprotonation at C2 generates the nucleophilic intermediate II, which then attacks the fluorinating reagent 2 to form the C–F bond, as illustrated in III. This C–F bond-forming step is expected to be enantiodetermining. Finally, product 3 is released *via* intermediate IV, completing the catalytic cycle.

To elucidate the origin of enantioselectivity, we performed docking and molecular dynamics (MD) simulations on intermediate II using the optimal mutant Sav-S112L–K121Q, cofactor 1, and substrate 1a (Fig. 4B). Cofactor 1 is stably positioned at the interface of two streptavidin monomers through the anchoring effect of its biotin unit. The deprotonated substrate 1a binds the Cu center through both oxygen atoms, adopting a tetrahedral coordination environment around the metal. This activated intermediate sits at the monomer–monomer interface, in close proximity to residues L112 and Q121.

As shown in Fig. 4C, residue Q121 sterically blocks one face of 1a, leaving the opposite face accessible to the incoming reagent 2, which leads to the observed *R* configuration. The steric profile of Q121 also discriminates between the bulky *t*-Bu ester and planar phenyl substituents, preventing 1a from adopting the alternative, 180°-rotated binding mode. This structural gating explains the progressively improved enantioselectivity from 3b to 3d and ultimately 3a.

## Conclusion

In conclusion, we have developed an artificial metalloenzyme catalyzed electrophilic C–F bond formation reaction, a reaction type remains challenging in biocatalysis. By integrating a biotinylated Lewis acidic Cu(ii) cofactor into the streptavidin scaffold, we achieved efficient and highly enantioselective α-fluorination, with optimized Sav-S112L–K121Q variants delivering up to 95% ee under mild conditions. Docking and MD studies established how cofactor positioning and the steric shielding imposed by Q121 govern the approach of the fluorinating reagent and thereby dictate enantioselectivity. Together, these results highlight the broader potential of ArMs to expand biocatalytic space toward transformations traditionally restricted to chemical catalysis.

## Author contributions

J. Y. and C. W. conducted the cofactor synthesis, reaction condition optimization, directed evolution and substrate scope investigation. W. H. conducted the MD simulation. H.-J. P., J. Yu., C. W. and W. H. wrote the manuscript with input from all authors. H.-J. P., H. W. and J. Z. coordinated and conceived the project.

## Conflicts of interest

The authors declare no competing interests.

## Supplementary Material

SC-OLF-D6SC00858E-s001

## Data Availability

All data for the replication of this work are given in the supplementary information (SI) or can be obtained by the lead contact upon reasonable request. Supplementary information is available. See DOI: https://doi.org/10.1039/d6sc00858e.
