# Supplementary material for: Enantioselective electrophilic α-fluorination catalyzed by an artificial metalloenzyme
Source: Chem Sci. 2026 Mar 24;17(19):9562–9. doi: 10.1039/d6sc00858e (PMC13011859; doi:10.1039/d6sc00858e)
Supplement: SC-017-D6SC00858E-s001 [file SC-017-D6SC00858E-s001.pdf]

Supplementary Information (SI) for Chemical Science.
This journal is © The Royal Society of Chemistry 2026

Supporting Information

Enantioselective Electrophilic α-Fluorination Catalyzed by an Artificial Metalloenzyme

Jinmeng Yu[1], Chang Wang[1], Wenhao Hu, Huan Wang, Jing Zhao, Hui-Jie Pan*

State Key Laboratory of Coordination Chemistry, Chemistry and Biomedicine Innovation Center (ChemBIC), ChemBioMed Interdisciplinary Research Center at Nanjing University, School of Chemistry and Chemical Engineering
Nanjing University
Nanjing 210023, P. R. China
E-mail: huijie.pan@nju.edu.cn

1. Jinmeng Yu and Chang Wang contribute equally.

TABLE OF CONTENTS

1. General Information 3
1.1 Materials and Reagents 3
1.2 Instrumentation 3
2. Supporting Experimental Tables 4
3 Directed Evolution and Mutant Creation 10
3.1 Materials and general methods for directed evolution 10
3.2 Mutant creation 10
4. Expression and Purification of Sav Mutants 11
4.1 General Procedure for the Expression of Sav 11
4.2 General Procedure for the Purification of Sav 11
4.3 Media and Buffers for Cell Growth and Cell Lysis, Dialysis, and Protein Purification 12
4.4 Sequence of Selected Sav Mutants 13
5. General procedure for the artificial metalloenzyme catalyzed fluorination reactions 14
6. Docking and MD simulation 15
6.1 Molecular Docking Methods 15
6.2 Molecular Dynamics Simulation Methods 15
7. Synthesis of Starting Materials 17
7.1 Synthesis of Substrates 17
7.2 Synthesis of Ligands and Cofactors 18
7.3 Preparation of Racemic Products 21
7.4 NMR and HPLC Spectra 30
8. HPLC Spectra of the products 54

1. General Information

1.1 Materials and Reagents

All chemical reagents were purchased from commercial suppliers (Aldrich Chemical, Alfa, J&K Chemical, Energy Chemical, Bide Pharmatech Ltd., Tianjin Xiensi Biochemical Technology Co., Ltd.) and used directly without further purification. All biological materials were obtained from commercial suppliers: PrimeSTAR Max DNA Polymerase and DpnI restriction enzyme were purchased from Takara Biomedical Technology Co., Ltd.; Escherichia coli BL21 (DE3) pLySs competent cells were sourced from Shanghai Weidi Biotechnology Co., Ltd.; oligonucleotide primers, ampicillin sodium, peptone, and yeast extract were acquired from Sangon Biotech Co., Ltd. (Shanghai, China); all genes were synthesized by TransGen Biotech Co., Ltd. (Beijing, China).

1.2 Instrumentation

Analytical thin layer chromatography (TLC) analyses were performed using 0.25 mm silica gel 60-F254 plates, with visualization achieved by UV light (254 nm). Flash column chromatography separations were carried out using silica gel (300-400 mesh). ^{1}H nuclear magnetic resonance (^{1}H NMR) spectra and ^{13}C nuclear magnetic resonance (^{13}C NMR) spectra were recorded at room temperature using a Bruker AMX-400 instrument (400 MHz) or a Bruker DRX-500 instrument (500 MHz).Chemical shifts are expressed in parts per million (δ) referenced to chloroform (7.26 ppm or 77.16 ppm), dichloromethane (5.32 ppm or 53.84 ppm), methanol (3.31 ppm or 49.00 ppm), acetonitrile (1.94 ppm or 118.26 ppm), dimethyl sulfoxide (2.50 or 39.52 ppm). The multiplicity of peaks is denoted as follows: s = singlet, d = doublet, t = triplet, q = quartet, dd = doublet of doublets, dt = doublet of triplets, td = triplet of doublets, ddd = doublet of doublet of doublets, etc., m = multiplet, br = broad. All spectra were analyzed using MestReNova software and calibrated against the residual solvent peaks. High-resolution mass spectra were recorded on a Thermo Fisher Q Exactive mass spectrometer using electrospray ionization-Orbitrap (ESI-Orbitrap) technology. High-performance liquid chromatography (HPLC) profiles were obtained using a Shimadzu LC-2050C HPLC system with commercially available normal-phase HPLC columns.

2. Supporting Experimental Tables

Table S1| Sequences of primers specific for Sav mutants

Sav_S112L_fw	CAGTGGCTGCTGACCCTGGGCACCACCGAGGCC
Sav_S112L_rv	GGCCTCGGTGGTGCCCAGGGTCAGCAGCCACTG
Sav_S121R_fw	GAGGCCAACGCCTGGCGCTCCACGCTGGTCGGC
Sav_S121R_rv	GCCGACCAGCGTGGAGCGCCAGGCGTTGGCCTC
Sav_S112L-K121Q_fw	GAGGCCAACGCCTGGCAGTCCACGCTGGTCGGC
Sav_S112L-K121Q_rv	GCCGACCAGCGTGGACTGCCAGGCGTTGGCCTC
Sav_S112V-K121R_fw	CAGTGGCTGCTGACCGTCGGCACCACCGAGGCC
Sav_S112V-K121R_rv	GGCCTCGGTGGTGCCGACGGTCAGCAGCCACTG
Sav_S112I-K121R_fw	CAGTGGCTGCTGACCATCGGCACCACCGAGGCC
Sav_S112I-K121R_rv	GGCCTCGGTGGTGCCGATGGTCAGCAGCCACTG
Sav_S112L-K121N_fw	GAGGCCAACGCCTGGAATTCCACGCTGGTCGGC
Sav_S112L-K121N_rv	GCCGACCAGCGTGGAATTCCAGGCGTTGGCCTC

Table S2| Screening of purified Sav variants in asymmetric fluorination

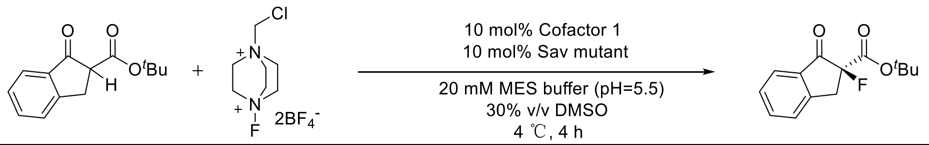

Entry	Sav mutant	Yield (%)	ee (%)
1	Sav WT	60	55
2	Sav S112A	83	65
3	Sav S112C	71	55
4	Sav S112D	71	61
5	Sav S112E	46	74
6	Sav S112F	42	43
7	Sav S112G	39	77
8	Sav S112H	25	43
9	Sav S112I	71	71
10	SavS112G	39	77
11	Sav S112K	54	17
12	Sav S112L	88	71
13	Sav S112M	42	79
14	Sav S112N	75	61
15	Sav S112Q	31	65
16	Sav S112T	79	65
17	Sav S112V	44	73
18	Sav S112W	55	31
19	Sav K121A	50	81
20	Sav K121E	46	81
21	Sav K121H	28	47
22	Sav K121N	47	83
23	Sav K121P	23	17
24	Sav K121Q	47	83
25	Sav K121R	71	85
26	Sav K121S	38	79
27	Sav K121Y	39	69
28	Sav K121I	51	89
29	Sav K121F	46	69
30	Sav K121M	47	89
31	Sav K121C	50	87
32	Sav K121T	46	74
33	Sav K121W	57	80
34	Sav K121L	53	88
35	Sav K121D	55	80

Standard conditions: [1] = 2.0 mM, [2] = 10.0 mM, [Sav mutant] = 20 μM, Cofactor 1=[biotin-Cu(phen)(NO_3)$_2$] (20 μM), 300 μL of MES buffer (20 mM, pH 5.5) and 30% DMSO (v/v) as co-solvent, 4 °C, 4 h. The total reaction volume was 500 μL. Yield and ee were determined by HPLC.

Table S3| Optimization of reaction temperature

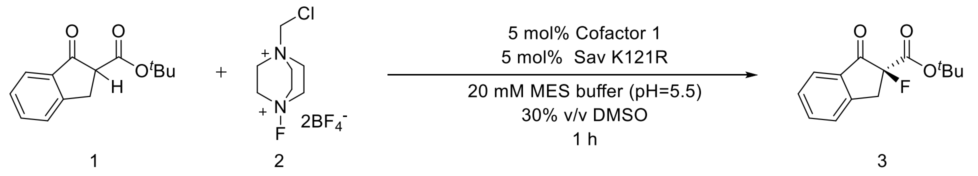

Entry	Reaction temperature (℃)	Yield (%)	ee (%)
1	0	66	89
2	4	69	89
3	20	56	88
4	25	55	87

Standard conditions: [1] = 2.0 mM, [2] = 10.0 mM, [Sav K121R] = 10 μM, Cofactor 1=[biotin-Cu(phen)(NO_3)$_2$] (10 μM), 300 μL of MES buffer (20 mM, pH 5.5) and 30% DMSO (v/v) as co-solvent. The total reaction volume was 500 μL. Yield and ee were determined by HPLC.

Table S4| Optimization of reaction time

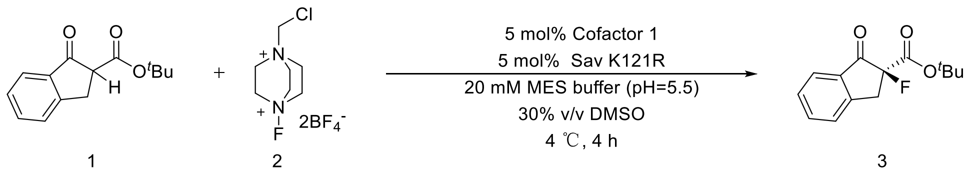

Entry	Deviation from the above conditions	Yield (%)	ee (%)
1	1 h	71	85
2	2 h	63	85
3	4 h	65	87
4	6 h	59	87
5	8 h	61	85
6	16 h	62	85
7	30 min	77	89
8	10 min	74	89

Standard conditions: [1] = 2.0 mM, [2] = 10.0 mM, [Sav K121R] = 10 μM, Cofactor 1=[biotin-Cu(phen)(NO_3)$_2$] (10 μM), 300 μL of MES buffer (20 mM, pH 5.5) and 30% DMSO (v/v) as co-solvent. The total reaction volume was 500 μL. Yield and ee were determined by HPLC.

Table S5| Screening of purified Sav S112L-K121X and S112X-K121R variants for asymmetric fluorination

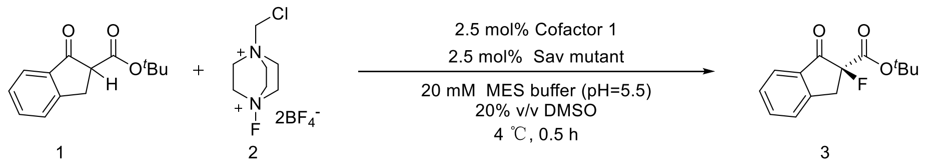

Entry	Sav mutant	Yield (%)	ee (%)
1	Sav S112L-K121C	47	77
2	Sav S112L-K121D	46	77
3	Sav S112L-K121E	68	84
4	Sav S112L-K121F	56	83
5	Sav S112L-K121G	59	83
6	Sav S112L-K121H	51	71
7	Sav S112L-K121I	71	83
8	Sav S112L-K121L	63	81
9	Sav S112L-K121N	62	87
10	Sav S112L-K121P	46	35
11	Sav S112L-K121Q	62	89
12	Sav S112L-K121R	53	83
13	Sav S112L-K121S	29	19
14	Sav S112L-K121T	46	71
15	Sav S112L-K121V	48	79
16	Sav S112L-K121W	65	77
17	Sav S112L-K121Y	57	79
18	Sav S112A-K121R	43	11
19	Sav S112C-K121R	61	77
20	Sav S112D-K121R	71	83
21	Sav S112E-K121R	26	23
22	Sav S112F-K121R	61	37
23	Sav S112G-K121R	37	37
24	Sav S112H-K121R	37	43
25	Sav S112I-K121R	73	79
26	Sav S112K-K121R	34	36
27	Sav S112L-K121R	48	43
28	Sav S112N-K121R	57	69
29	Sav S112P-K121R	58	55
30	Sav S112Q-K121R	63	71
31	Sav S112R-K121R	36	9
32	Sav S112T-K121R	65	19
33	Sav S112V-K121R	69	84
34	Sav S112W-K121R	36	51
35	Sav S112Y-K121R	83	43

Standard conditions: [1] = 2.0 mM, [2] = 10.0 mM, [Sav mutant] = 5 μM, Cofactor1 =[biotin-Cu(phen)(NO_3)$_2$] (5 μM), 300 μL of MES buffer (20 mM, pH 5.5) and 20% DMSO (v/v) as co-solvent, 4 °C, 0.5 h. The total reaction volume was 500 μL. Yield and ee were determined by HPLC.

Table S6| Optimization of reaction buffer

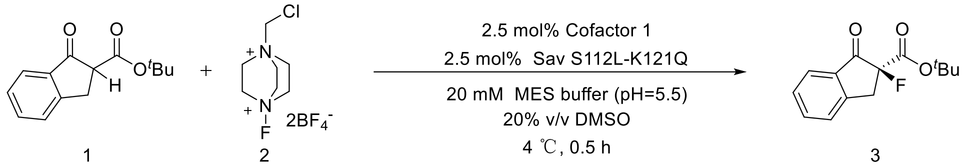

Entry	Variations of standard conditions	Yield (%)	ee (%)
1	20 mM MES pH=5.0	43	87
2	20 mM MES pH=5.5	62	89
3	20 mM MES pH=6.0	53	79
4	20 mM MES pH=6.5	42	53
5	20 mM MOPS pH=5.5	51	59
6	20 mM MOPS pH=6.0	71	85
7	20 mM MOPS pH=6.5	70	77
8	20 mM MOPS pH=7.0	70	77
9	20 mM MOPS pH=7.5	47	27
10	50 mM MES pH=5.5	79	66
11	100 mM MES pH=5.5	52	43
12	10 mM PBS pH=7.4	70	7
13	20 mM Tris-Hcl pH=7.4	83	3
14	20 mM Citric acid pH=6.0	72	0

Standard conditions: [1] = 2.0 mM, [2] = 10.0 mM, [Sav S112L-K121Q] = 5 μM, Cofactor 1=[biotin-Cu(phen)$(NO_3)_2$] (5 μM), 300 μL of MES buffer (20 mM, pH 5.5) and 20% DMSO (v/v) as co-solvent, 4 °C, 0.5 h. The total reaction volume was 500 μL. Yield and ee were determined by HPLC.

Table S7| Optimization of methanol concentration as a cosolvent for Sav-catalyzed asymmetric fluorination

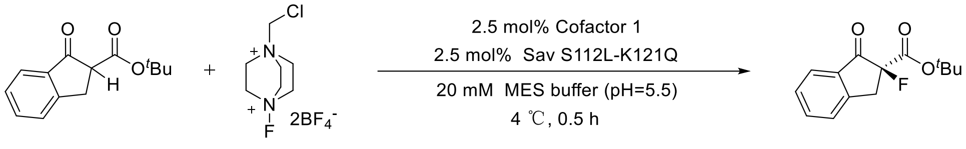

Entry	Deviation from the above conditions	Yield (%)	ee (%)
1	12.5% MeOH	91	93
2	15% MeOH	97	89
3	20% MeOH	81	86

Standard conditions: [1] = 2.0 mM, [2] = 10.0 mM, [Sav S112L-K121Q] = 5 μM, Cofactor 1 [biotin-Cu(phen)(NO_3)$_2$] (5 μM), 300 μL of MES buffer (20 mM, pH 5.5), 4 °C, 0.5 h. The total reaction volume was 500 μL. Yield and ee were determined by HPLC.

Table S8| Results of the free radical trapping experiment

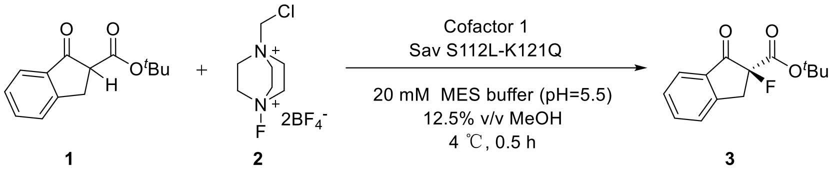

Entry	Sav mutant (2.5 mol%)	Cofactor (2.5 mol%)	Deviation from the above conditions	Yield (%)	ee (%)
1	S112L-K121Q	cofactor **1**	--	91	93
2	S112L-K121Q	cofactor **1**	with 1.0 eq TEMPO	85	82
3	--	--	--	30	0
4	--	--	with 1.0 eq TEMPO	18	0

Standard conditions: [1] = 2.0 mM, [2] = 10.0 mM, [Sav S112L-K121Q] = 5 μM, Cofactor 1 [biotin-Cu(phen)(NO_3)$_2$] (5 μM), 300 μL of MES buffer (20 mM, pH 5.5) and 12.5% MeOH (v/v) as co-solvent, 4 °C, 0.5 h. The total reaction volume was 500 μL. Yield and ee were determined by HPLC.

3 Directed Evolution and Mutant Creation

3.1 Materials and general methods for directed evolution

Molecular biology reagents and chemicals were purchased from Takara Biomedical Technology Co., Ltd., unless otherwise noted. Primers were synthesized by Sangon Biotech Co., Ltd. (Shanghai, China) and their sequences are listed in Table S2.

3.2 Mutant creation

Primers were designed using a two-step PCR approach. In the first step, a primer containing the desired mutagenic sites and a silent primer were used to generate a short DNA fragment. This fragment was subsequently recovered and employed as a megaprimer in the second step to amplify the entire plasmid. The PCR product from the second step was digested with the DpnI restriction enzyme, after which 10 μL of the resulting mixture was transformed into chemically competent Escherichia coli BL21(DE3) cells. Single colonies from the transformation plates were selected and cultured overnight at 37 °C in LB medium containing ampicillin (100 μg/mL). The overnight cultures were centrifuged, and plasmid DNA was purified using the Sangon Plasmid Miniprep Kit. The purified plasmids were then sequenced to confirm the presence of the desired mutations.

4. Expression and Purification of Sav Mutants

4.1 General Procedure for the Expression of Sav

For protein expression, plasmids harboring Sav variants were transformed into Escherichia coli BL21 (DE3) pLySs competent cells. Subsequently, the selected single colonies were inoculated into 8 mL of LB medium containing 60 µg/mL ampicillin and 34 µg/mL chloramphenicol, and cultured overnight at 37 °C with shaking at 220 rpm to obtain a preculture.

Next, 8 mL of the preculture was transferred into 800 mL of ZYP-5052 main medium (supplemented with 100 µg/mL ampicillin and 34 µg/mL chloramphenicol), followed by incubation at 30 °C with shaking at 220 rpm for 24 hours to conduct the main culture. After the completion of culture, bacterial cells were harvested by centrifugation at 8000 rpm for 10 min, and finally, the collected cells were stored frozen at -20 °C for subsequent use.

4.2 General Procedure for the Purification of Sav

Thawed cell pellets were resuspended in lysis buffer, and the suspension was transferred to a high-pressure homogenizer for cell disruption to obtain the cell extract (CE). The CE was loaded into a dialysis bag and subjected to three-step dialysis: first, dialyzed against 6 mol/L guanidine hydrochloride (GdmCl) dialysis buffer for ≤ 24 h; second, dialyzed against 20 mmol/L Tris-HCl buffer (pH 7.4) for 24 h; finally, dialyzed against imino biotin binding buffer (IBB) for ≥ 24 h (this step was performed at 4 °C). The clarified supernatant was collected by centrifugation (4 °C, 10,000 rpm, 90 min) and loaded onto a 2-iminobiotin agarose resin column for affinity purification. The column was first equilibrated with 5 column volumes (CV) of IBB buffer, then eluted with Milli-Q water supplemented with 1% (v/v) acetic acid (HOAc) to obtain the purified Sav mutant protein.

To remove acetic acid from the eluate, re-dialysis was performed: the protein solution was first dialyzed against 10 mmol/L Tris-HCl buffer (pH 7.4), followed by two cycles of dialysis against Milli-Q water. Finally, the protein solution was filtered through a 0.45 µm pore-size membrane to remove precipitates, snap-frozen in liquid nitrogen, and lyophilized using a freeze dryer.

4.3 Media and Buffers for Cell Growth and Cell Lysis, Dialysis, and Protein Purification

20× ZYP salts: 136 g of KH_2PO_4(50 mM), 142 g of Na_2HPO_4 (50 mM), and 66 g of $(NH_4)_2SO_4$ (25 mM) are dissolved in DI-H_2O (final volume of 1 L) and autoclaved (20 min, 121 °C, 1.5 bar).

20× ZYP sugars: 100 g of glycerol (10% v/v), 11 g of glucose-monohydrate (1.1% w/v), and 40 g of α-lactose monohydrate (4% w/v) are dissolved in DI-H_2O (final volume of 1 L) and autoclaved (20 min, 121 °C, 1.5 bar).

200 mM $MgSO_4$: 4.8 g of anhydrous $MgSO_4$ is dissolved in DI-H_2O (final volume of 200 mL) and autoclaved (20 min, 121 °C, 1.5 bar).

Auto-Induction (ZYP-5052) Medium: Dissolve 50 g of tryptone (1% w/v) and 25 g of yeast extract (0.5% w/v) in 4.45 L of DI-H_2O. Dispense the solution into bottles, 712 mL per bottle, and autoclave (30 minutes at 121 °C). Cool the medium to 55 °C or lower, then add 40 mL of 20× ZYP salts, 40 mL of 20× ZYP sugars, and 8 mL of 200 mM $MgSO_4$ to the medium.

Lysis buffer: To make 1.5 L of a 20 mM Tris–HCl, pH 7.4 buffer, 30 mL of 1 M Tris–HCl, pH 7.4 is diluted to a final volume of 1.5 L with DI-H_2O.

6 M guanidinium chloride (GdmCl) dialysis buffer, pH 1.5: 14.3 kg of GdmCl is dissolved in DI-H_2O. To adjust the pH to 1.5, 37% HCl is added. DI-H_2O is added to a total volume of 25 L.

20 mM (10 mM) Tris–HCl dialysis buffer, pH 7.4: 500 mL(250mL) of 1M Tris–HCl, pH 7.4 is diluted to a final volume of 25 L with DI-H_2O.

Iminobiotin binding (IBB) dialysis buffer, pH 10.8:105g of $NaHCO_3$ (final concentration 50 mM) and 730 g of NaCl (final concentration 0.5 M) are dissolved in DI-H_2O. The pH is adjusted to 10.8 with 5 M NaOH before DI-H_2O is added to a total volume of 25 L and stored at 4 °C.

Iminobiotin binding (IBB) buffer, pH 10.8: 8.4 g of $NaHCO_3$ (50 mM) and 58.4 g of NaCl (0.5 M) are dissolved in Milli-Q water. The pH is adjusted to 10.8 with 5 M NaOH before Milli-Q water is added to a total volume of 2 L.

Elution buffer: 20 mL of HOAc (1% v/v) is mixed with Milli-Q water (final volume of 2L).

4.4 Sequence of Selected Sav Mutants

Amino acid sequence of Sav WT
MASMTGGQQMGRDQAGITGTWYNQLGSTFIVTAGADGALTGTYESAVGNAE
SRYVLTGRYDSAPATDGSGTALGWTVAWKNNYRNAHSATTWSGQYVGGAEA
RINTQWLLTSGTTEANAWKSTLVGHDTFTKVKPSAASIDAAKKAGVNNGNPL
DAVQQ

Amino acid sequence of Sav S112L
MASMTGGQQMGRDQAGITGTWYNQLGSTFIVTAGADGALTGTYESAVGNAE
SRYVLTGRYDSAPATDGSGTALGWTVAWKNNYRNAHSATTWSGQYVGGAEA
RINTQWLLT**L**GTTEANAWKSTLVGHDTFTKVKPSAASIDAAKKAGVNNGNPL
DAVQQ

Amino acid sequence of Sav K121R
MASMTGGQQMGRDQAGITGTWYNQLGSTFIVTAGADGALTGTYESAVGNAE
SRYVLTGRYDSAPATDGSGTALGWTVAWKNNYRNAHSATTWSGQYVGGAEA
RINTQWLLTSGTTEANAW**R**STLVGHDTFTKVKPSAASIDAAKKAGVNNGNPL
DAVQQ

Amino acid sequence of Sav S112L-K121Q
MASMTGGQQMGRDQAGITGTWYNQLGSTFIVTAGADGALTGTYESAVGNAE
SRYVLTGRYDSAPATDGSGTALGWTVAWKNNYRNAHSATTWSGQYVGGAEA
RINTQWLLT**L**GTTEANAW**Q**STLVGHDTFTKVKPSAASIDAAKKAGVNNGNPL
DAVQQ

Amino acid sequence of Sav S112V-K121R
MASMTGGQQMGRDQAGITGTWYNQLGSTFIVTAGADGALTGTYESAVGNAE
SRYVLTGRYDSAPATDGSGTALGWTVAWKNNYRNAHSATTWSGQYVGGAEA
RINTQWLLT**V**GTTEANAW**R**STLVGHDTFTKVKPSAASIDAAKKAGVNNGNPL
DAVQQ

Amino acid sequence of Sav S112I-K121R
MASMTGGQQMGRDQAGITGTWYNQLGSTFIVTAGADGALTGTYESAVGNAE
SRYVLTGRYDSAPATDGSGTALGWTVAWKNNYRNAHSATTWSGQYVGGAEA
RINTQWLLT**I**GTTEANAW**R**STLVGHDTFTKVKPSAASIDAAKKAGVNNGNPL
DAVQQ

Amino acid sequence of Sav S112L-K121N
MASMTGGQQMGRDQAGITGTWYNQLGSTFIVTAGADGALTGTYESAVGNAE
SRYVLTGRYDSAPATDGSGTALGWTVAWKNNYRNAHSATTWSGQYVGGAEA
RINTQWLLT**L**GTTEANAW**N**STLVGHDTFTKVKPSAASIDAAKKAGVNNGNPL
DAVQQ

5. General procedure for the artificial metalloenzyme catalyzed fluorination reactions

The reaction mixture (total volume 0.5 mL) consisted of purified Sav S112L-K121Q (2.5 mol%), cofactor 1 (2.5 mol%), substrate 1 (2 mM), and substrate 2 (10 mM), dissolved in 20 mM MES buffer (pH 5.5) containing 12.5-15% (v/v) methanol (MeOH). The reaction was carried out at 4 °C for 0.5 h in a metal bath. Upon completion of the reaction, an internal standard, 9-thioxanthen-9-one dissolved in ethyl acetate (5 mM, 50 μL), was added, and the mixture was subsequently extracted with ethyl acetate. The organic phase was dried over anhydrous sodium sulfate. The yield and enantiomeric excess (ee) of the product were determined by normal-phase high-performance liquid chromatography (HPLC).

6. Docking and MD simulation

6.1 Molecular Docking Methods

The crystal structure of streptavidin (PDB ID: 5K67) was used as the template for generating Sav mutants. Mutagenesis and structural inspection were performed in PyMOL (version 3.1.3). Molecular docking was carried out with AutoDock Vina (version 1.2.5)[1]. Docking boxes and binding-site coordinates were defined using the GetBox plugin in PyMOL. Ligands were prepared with AutoDockTools[2] by adding hydrogens, assigning Gasteiger charges, and converting structures to PDBQT format. Protein structures were processed following the standard AutoDock Vina workflow.

6.2 Molecular Dynamics Simulation Methods

Molecular dynamics (MD) simulations were performed using the Amber 24.0 package[3]. The protein was parameterized with the AMBER ff14SB force field, while ligand parameters and partial charges were generated using AmberTools. Metal coordination parameters for the Cu(II) center were obtained using the MCPB module. The docked complex was solvated in a rectangular TIP3P water box with a 1.0 nm buffer from the protein surface. A cutoff of 0.8 nm was applied for van der Waals and short-range electrostatic interactions, and long-range electrostatics were treated with the particle mesh Ewald (PME) method. A 2 fs integration step was used throughout. The system was maintained at 300 K and 1 bar.

The system underwent a two-stage minimization (protein-backbone-restrained followed by full minimization), followed by 100 ps of NVT and 100 ps of NPT equilibration. A 10 ns pre-production run was conducted to relax the system further, after which 200 ns of production MD was performed using Berendsen thermostat and barostat coupling. Analyses including RMSD and interatomic distance calculations were performed with cpptraj, and structural visualization was carried out in PyMOL.

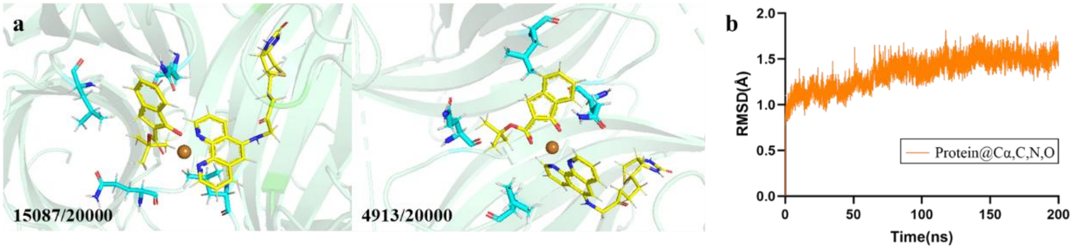

Figure S1 (a) Two most populated structures by clustering of the MD trajectory during 200 ns MD simulation. The number of frames for the two corresponding conformations is shown at the bottom left corner. (b) RMSD of the Sav-S112L-K121Q backbone atoms (Ca, C, N, O) during 200 ns MD simulations.

7. Synthesis of Starting Materials

7.1 Synthesis of Substrates

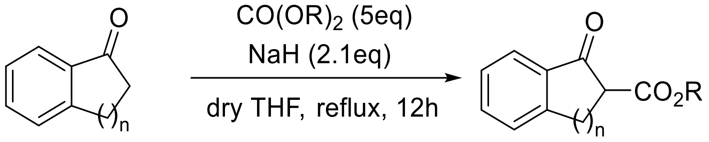

General procedure A: A solution of ketone (1.0 eq.) in abs. THF was added dropwise to a stirred solution of dialkyl carbonate (5.0 eq.) in abs. THF containing NaH (60% dispersion in mineral oil, 2.1 eq.) under a nitrogen atmosphere. The mixture was heated to reflux (12 h). After completion, cooled in an ice-bath and then acidified with 1M HCl. The residue was then extracted with EA for three times. The combined organic layers were dried over Na_2SO_4, filtered and the solvent was removed in vacuo. The crude product was purified by column chromatography to give the corresponding products.

1a[4], 1b[5], 1c[6], 1d[7], 1m[8], 1s[9] were in accordance with literature values.

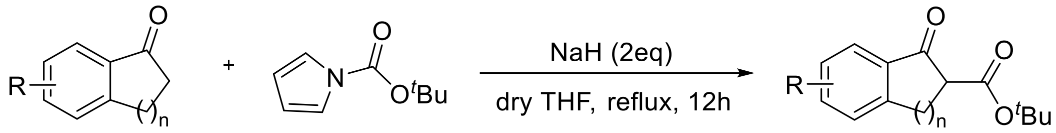

General procedure B: A solution of ketone (1.0 eq.) in abs. THF was added dropwise to a stirred solution of NaH (60% dispersion in mineral oil, 2 eq.) in abs. THF under a nitrogen atmosphere. The mixture was stirred at 25 ºC for 20min. The *t*-Butyl 1H-pyrrole-1-carboxylate was added and the mixture was heated to reflux (12 h). After completion, cooled in an ice-bath and then acidified with 1M HCl. The residue was then extracted with EA for three times. The combined organic layers were dried over Na_2SO_4, filtered and the solvent was removed in vacuo. The crude product was purified by column chromatography to give the corresponding products.

1e[9], 1f[10] 1g[11], 1h[12], 1i[13], 1j[14], 1k[14], 1l[13], 1n[7], 1o[15], 1p[9], 1q[16], 1r[12], 1t[14],1u[17] and 1v[18] were in accordance with literature values.

7.2 Synthesis of Ligands and Cofactors

Synthesis of Ligand L1[19]

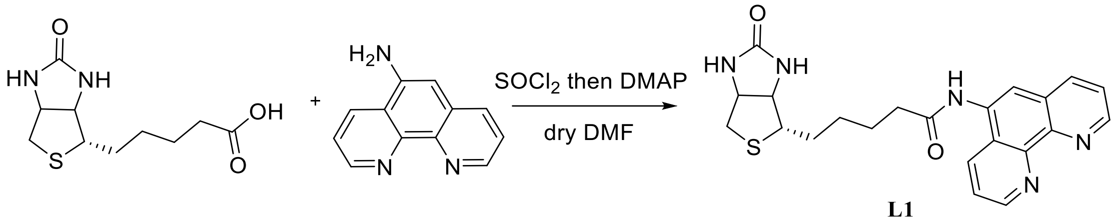

L1

Biotin (1.8 mmol) was dissolved in $SOCl_2$ in a dry round bottomed flask. The reaction mixture was stirred for 20 minutes, followed by remove excess thionyl chloride. The residue was redissolved in dry DMF (15 mL). 5-amino-phenanthroline (2 mmol) and DMAP (0.23 mmol) were dissolved in dry DMF (30 mL), to which the biotin-acyl chloride in DMF was transferred. The reaction was allowed to stir for 18 hours at room temperature, and then concentrated to ~ 3 ml, after which it was poured in ether (30 mL) while stirring. A black precipitate resulted. The solution was decanted and the solid was dissolved in hot methanol. The amide was purified using deactivated alumina chromatography.

^{1}H NMR (400 MHz, DMSO-*d*$_6$) δ 10.14 (s, 1H), 9.13 (dd, *J* = 4.4, 1.7 Hz, 1H), 9.03 (dd, *J* = 4.4, 1.8 Hz, 1H), 8.64 (dd, *J* = 8.4, 1.7 Hz, 1H), 8.49 (dd, *J* = 8.1, 1.9 Hz, 1H), 8.20 (s, 1H), 7.86 (dd, *J* = 8.5, 4.3 Hz, 1H), 7.77 (dd, *J* = 8.1, 4.4 Hz, 1H), 6.47 (s, 1H), 6.39 (s, 1H), 4.38- 4.28 (m, 1H), 4.22-4.12 (m, 1H), 3.20-3.09 (m, 1H), 2.85 (dd, *J* = 12.4, 5.2 Hz, 1H), 2.61 (d, *J* = 12.4 Hz, 1H), 2.55 (t, 2H), 1.80-1.43 (m, 6H).
^{13}C NMR (101 MHz, DMSO-*d*$_6$) δ 172.9, 163.2, 150.4, 149.7, 145.9, 143.8, 136.7, 132.5, 132.4, 128.6, 125.2, 124.2, 123.5, 120.4, 61.6, 59.7, 55.9, 36.2, 28.8, 28.6, 25.7.
HRMS (ESI) m/z calcd for $C_{22}H_{24}N_5O_2S^+$ $(M+H)^+$ 422.1645, found 422.1633.

Synthesis of Ligand L2

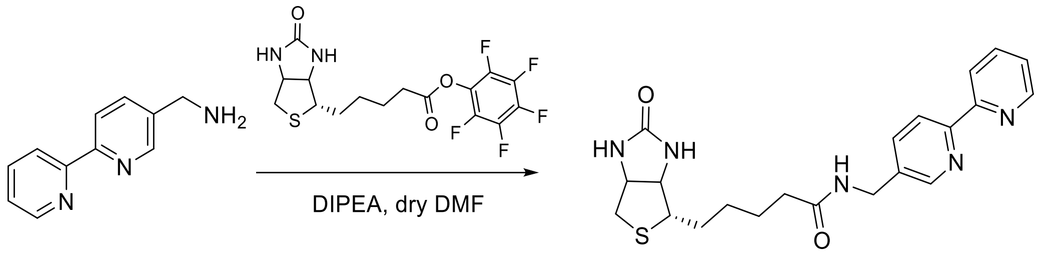

L2

[2,2'-bipyridin]-5-ylmethanamine[20] (1 mmol) and Biotin pentafluorophenyl ester[21] (1.5 mmol) (1.5 mol, 1.0 eq.) was dissolved in 8 ml of dry DMF. To this solution, DIPEA (3 mmol) was added. The reaction mixture allowed to stir for 18 hours at 40°C. Upon completion of the reaction, the mixture was concentrated under reduced pressure to remove DMF, and the residue was purified by flash chromatography on silica gel to give the desired product L2.

^{1}H NMR (400 MHz, DMSO-*d6*) δ 8.67 (d, J = 4.7 Hz, 1H), 8.57 (s, 1H), 8.46-8.31 (m, 3H), 7.93 (t, J = 7.6 Hz, 1H), 7.79 (d, J = 8.0 Hz, 1H), 7.44 (t, J = 6.1 Hz, 1H), 6.43 (s, 1H), 6.36 (s, 1H), 4.35 (d, J = 5.8 Hz, 2H), 4.33-4.25 (m, 1H), 4.11 (m, 1H), 3.08 (dt, J = 10.4, 5.4 Hz, 1H), 2.81 (dd, J = 12.4, 5.1 Hz, 1H), 2.57 (d, J = 12.4 Hz, 1H), 2.16 (t, J = 7.5 Hz, 2H), 1.64 – 1.28 (m, 6H).

^{13}C NMR (101 MHz, DMSO-d_6) δ 172.8, 163.2, 155.6, 154.4, 149.7, 148.8, 137.7, 136.6, 136.2, 124.5, 120.8, 120.6, 61.5, 59.7, 55.9, 35.6, 28.7, 28.5, 25.7.

HRMS (ESI) m/z calcd for $C_{21}H_{26}N_5O_2S^+$ (M+H)$^+$ 412.1802, found 412.1781.

Synthesis of Cofactor 1

A solution of ligand L1 (0.05 mmol) in ethanol was added dropwise to an ethanolic solution of $Cu(NO_3)_2 \cdot 3H_2O$ (0.055 mmol) under a nitrogen atmosphere. The resulting mixture was stirred at 25 °C for 12 h (overnight). After completion of the reaction, the precipitate was collected by filtration, and the filtrate was concentrated to dryness under reduced pressure via rotary evaporation. The residue was triturated with a small volume of ethanol followed by a large excess of petroleum ether (PE), and the mixture was sonicated to facilitate crystallization. The supernatant was decanted, and the remaining solid was concentrated to dryness under reduced pressure for subsequent use.

Synthesis of Cofactor 2

Under a nitrogen atmosphere, an ethanolic solution of $Cu(NO_3)_2 \cdot 3H_2O$ (0.011 mmol) was added dropwise to a solution of ligand L2 (0.01 mmol) in ethanol. The reaction mixture was stirred at ambient temperature (25 °C) for 1 h, during which the solution turned blue-green (indicating complex formation). After completion of the reaction, the mixture was filtered to remove any insoluble impurities. The filtrate was concentrated to dryness under reduced pressure via rotary evaporation. The resulting residue was washed with a dichloromethane/methanol (DCM/MeOH) mixed solvent, and the target product was precipitated by the addition of diethyl ether (Et_2O). The precipitate was collected and dried under vacuum for subsequent use.

7.3 Preparation of Racemic Products

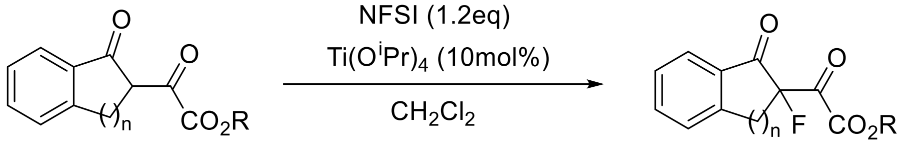

The stirring mixture of appropriate 1a-t (0.5 mmol), NFSI (0.6 mmol) in CH_2Cl_2(5.0 mL) was added $Ti(O^iPr)_4$ (0.05 mmol) and stirred at room temperature under nitrogen atmosphere. The reaction was monitored by TLC until the starting material was completed. The resulting mixture was quenched with saturated $NaHCO_3$ aqueous solution and extracted with CH_2Cl_2 three times. The organic layer was washed with brine, dried with Na_2SO_4 and concentrated under reduced pressure. The residue was purified by silica gel column chromatography to give the products 3a-v[22].

tert-butyl 2-fluoro-1-oxo-2,3-dihydro-1_H_-indene-2-carboxylate (_rac_-3a)

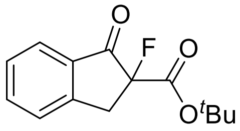

Using the typical procedure afforded racemic 3a as a colorless oil (chromatography on silica gel, _n_-hexane:ethyl acetate=60:1).

^{1}H NMR (400 MHz, Chloroform-_d_) δ 7.82 (d, _J_ = 7.7 Hz, 1H), 7.68 (td, _J_ = 7.5, 1.2 Hz, 1H), 7.52 – 7.40 (m, 2H), 3.72 (dd, _J_ = 17.5, 10.8 Hz, 1H), 3.39 (dd, _J_ = 22.9, 17.5 Hz, 1H), 1.42 (s, 9H).

^{13}C NMR (101 MHz, Chloroform-_d_) δ 195.8 (d, _J_ = 18.6 Hz), 166.2 (d, _J_ = 27.7 Hz), 151.0 (d, _J_ = 3.9 Hz), 136.4, 133.6, 128.5, 126.5, 125.4, 94.4 (d, _J_ = 201.7 Hz), 84.1, 38.3 (d, _J_ = 24.1 Hz), 27.8.

HRMS (ESI) m/z calcd for $C_{14}H_{15}FNaO_3^+$ $(M+Na)^+$ 273.0897, found 273.0891.

methyl 2-fluoro-1-oxo-2,3-dihydro-1_H_-indene-2-carboxylate (_rac_-3b)

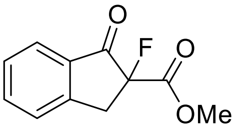

Using the typical procedure afforded racemic 3b as a yellow solid (chromatography on silica gel, _n_-hexane:ethyl acetate=60:1).

^{1}H NMR (400 MHz, Chloroform-_d_) δ 7.84 (d, _J_ = 7.7 Hz, 1H), 7.71 (td, _J_ = 7.5, 1.2 Hz, 1H), 7.55-7.43 (m, 2H), 3.81 (s, 4H), 3.44 (dd, _J_ = 23.3, 17.7 Hz, 1H).

^{13}C NMR (101 MHz, Chloroform-*d*) δ 195.1 (d, *J* = 18.1 Hz), 167.7 (d, *J* = 28.0 Hz), 150.8 (d, *J* = 3.8 Hz), 136.8, 133.2, 128.7, 126.6, 125.7, 94.6 (d, *J* = 201.6 Hz), 53.3, 38.3 (d, *J* = 23.8 Hz).

HRMS (ESI) m/z calcd for $C_{11}H_{10}FO_3^+$ $(M+H)^+$ 209.0608, found 209.0607.

ethyl 2-fluoro-1-oxo-2,3-dihydro-1*H*-indene-2-carboxylate (*rac*-3c)

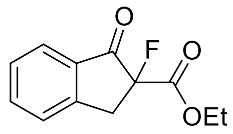

Using the typical procedure afforded racemic 3c as a yellow solid (chromatography on silica gel, *n*-hexane:ethyl acetate=60:1).

^{1}H NMR (400 MHz, Chloroform-d) δ 7.87-7.81 (m, 1H), 7.71 (td, J = 7.6, 1.3 Hz, 1H), 7.55-7.41 (m, 2H), 4.28 (q, J = 7.1 Hz, 2H), 3.97-3.68 (m, 1H), 3.63-3.30 (m, 1H), 1.26 (t, J = 7.1 Hz, 3H).

^{13}C NMR (101 MHz, Chloroform-*d*) δ 195.3 (d, *J* = 18.1 Hz), 167.3 (d, *J* = 27.7 Hz), 150.9 (d, *J* = 3.6 Hz), 136.7, 133.3, 128.6, 126.6, 125.6, 94.5 (d, *J* = 201.6 Hz), 62.6, 38.3 (d, *J* = 23.8 Hz), 14.0.

HRMS (ESI) m/z calcd for $C_{12}H_{11}FNaO_3^+$ $(M+Na)^+$ 245.0584, found 245.0581.

isopropyl 2-fluoro-1-oxo-2,3-dihydro-1*H*-indene-2-carboxylate (*rac*-3d)

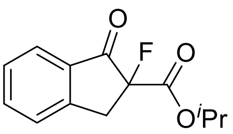

Using the typical procedure afforded racemic 3d as a yellow solid (chromatography on silica gel, *n*-hexane:ethyl acetate=60:1).

^{1}H NMR (400 MHz, Chloroform-*d*) δ 7.82 (d, *J* = 7.7 Hz, 1H), 7.69 (td, *J* = 7.5, 1.2 Hz, 1H), 7.52-7.41 (m, 2H), 5.24-5.03 (m, 1H), 3.75 (dd, *J* = 17.6, 11.8 Hz, 1H), 3.41 (dd, *J* = 23.3, 17.6 Hz, 1H), 1.23 (dd, *J* = 9.9, 6.3 Hz, 6H).

^{13}C NMR (101 MHz, Chloroform-*d*) δ 195.3, 166.9 (d, *J* = 27.5 Hz), 151.0 (d, *J* = 3.6 Hz), 136.6, 133.3, 128.6, 126.6, 125.6, 94.4 (d, *J* = 201.4 Hz), 70.7, 38.3 (d, *J* = 23.9 Hz), 21.5 (d, *J* = 11.0 Hz).

HRMS (ESI) m/z calcd for $C_{13}H_{13}FNaO_3^+$ $(M+Na)^+$ 259.0741, found 259.0736.

***tert*-butyl 2-fluoro-6-methyl-1-oxo-2,3-dihydro-1*H*-indene-2-carboxylate (*rac*-3e)**

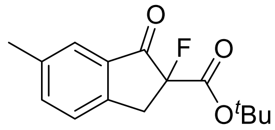

Using the typical procedure afforded racemic 3d as a yellow solid (chromatography on silica gel, *n*-hexane:ethyl acetate=60:1).

^{1}H NMR (400 MHz, Chloroform-*d*) δ 7.61 (s, 1H), 7.54-7.46 (m, 1H), 7.38 (d, *J* = 7.9 Hz, 1H), 3.67 (dd, 1H), 3.34 (dd, *J* = 22.9, 17.4 Hz, 1H), 2.42 (s, 3H), 1.43 (s, 9H).

^{13}C NMR (101 MHz, Chloroform-d) δ 195.8 (d, J = 18.4 Hz), 166.4 (d, J = 27.9 Hz), 148.4 (d, J = 3.8 Hz), 138.6, 137.7, 133.7 (d, J = 1.3 Hz), 126.1 (d, J = 1.4 Hz), 125.2 (d, J = 1.3 Hz), 94.7 (d, J = 201.5 Hz), 84.0, 38.0 (d, J = 24.1 Hz), 27.8, 21.1.

HRMS (ESI) m/z calcd for $C_{15}H_{17}FNaO_3^+$ $(M+Na)^+$ 287.1054, found 287.1048.

***tert*-butyl 2,6-difluoro-1-oxo-2,3-dihydro-1*H*-indene-2-carboxylate (*rac*-3f)**

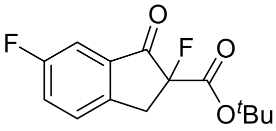

Using the typical procedure afforded racemic 3f as a colorless oil (chromatography on silica gel, *n*-hexane:ethyl acetate=60:1).

^{1}H NMR (500 MHz, Chloroform-*d*) δ 7.50-7.43 (m, 2H), 7.42-7.37 (m, 1H), 3.73-3.63 (m, 1H), 3.41-3.29 (m, 1H), 1.42 (s, 9H).

^{13}C NMR (126 MHz, Chloroform-*d*) δ 195.0 (d, *J* = 2.9 Hz), 165.8 (d, *J* = 27.7 Hz), 162.6 (d, *J* = 250.2 Hz), 146.4 (d, *J* = 4.0 Hz), 135.3 (d, *J* = 7.6 Hz), 128.0 (dd, *J* = 8.1, 1.5 Hz), 124.2 (d, *J* = 23.7 Hz), 111.2 (d, *J* = 22.3 Hz), 94.8 (d, *J* = 202.8 Hz), 84.4, 37.8 (d, *J* = 24.3 Hz), 27.8.

HRMS (ESI) m/z calcd for $C_{14}H_{14}F_2KO_3^+$ $(M+K)^+$ 307.0543, found 307.0535.

***tert*-butyl 6-chloro-2-fluoro-1-oxo-2,3-dihydro-1*H*-indene-2-carboxylate (*rac*-3g)**

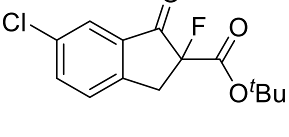

Using the typical procedure afforded racemic 3g as a colorless oil (chromatography on silica gel, *n*-hexane:ethyl acetate=60:1).

^{1}H NMR (500 MHz, Chloroform-*d*) δ 7.78 (d, *J* = 2.1 Hz, 1H), 7.64 (dd, *J* = 8.2, 2.1 Hz, 1H), 7.44 (d, *J* = 8.2 Hz, 1H), 3.69 (dd, *J* = 17.6, 10.4 Hz, 1H), 3.41-3.29 (m, 1H), 1.43 (s, 9H).

^{13}C NMR (126 MHz, Chloroform-*d*) δ 194.6 (d, *J* = 18.6 Hz), 165.8 (d, *J* = 27.7 Hz), 149.0 (d, *J* = 4.0 Hz), 136.4, 135.0, 134.9, 127.7 (d, *J* = 1.2 Hz), 125.1, 94.5 (d, *J* = 202.9 Hz), 84.5, 37.9 (d, *J* = 24.3 Hz), 27.8.

HRMS (ESI) m/z calcd for $C_{14}H_{14}ClFNaO_3^+$ $(M+Na)^+$ 307.0508, found 307.0502.

***tert*-butyl 6-bromo-2-fluoro-1-oxo-2,3-dihydro-1*H*-indene-2-carboxylate (*rac*-3h)[22]**

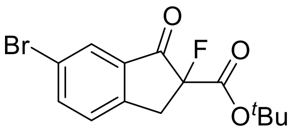

Using the typical procedure afforded racemic 3h as a white solid (chromatography on silica gel, *n*-hexane:ethyl acetate=60:1).

^{1}H NMR (400 MHz, Chloroform-*d*) δ 7.93 (d, *J* = 1.9 Hz, 1H), 7.77 (dd, *J* = 8.2, 2.0 Hz, 1H), 7.38 (d, *J* = 8.4 Hz, 1H), 3.66 (dd, *J* = 17.7, 10.5 Hz, 1H), 3.32 (m, 1H), 1.42 (s, 9H).

^{13}C NMR (101 MHz, Chloroform-*d*) δ 194.4 (d, *J* = 18.7 Hz), 165.8 (d, *J* = 27.7 Hz), 149.5 (d, *J* = 4.0 Hz), 139.2, 135.3, 128.2, 128.0 (d, *J* = 1.4 Hz), 122.6, 94.3 (d, *J* = 203.1 Hz), 84.5, 38.0 (d, *J* = 24.3 Hz), 27.8.

***tert*-butyl-2-fluoro-1-oxo-6-(trifluoromethyl)-2,3-dihydro-1*H*-indene-2-carboxylate (*rac*-3i)**

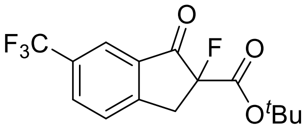

Using the typical procedure afforded racemic 3i as a yellow solid (chromatography on silica gel, *n*-hexane:ethyl acetate=60:1).

^{1}H NMR (500 MHz, Chloroform-*d*) δ 8.08 (s, 1H), 7.93 (d, *J* = 8.1 Hz, 1H), 7.65 (d, *J* = 8.1 Hz, 1H), 3.79 (dd, *J* = 17.9, 10.6 Hz, 1H), 3.46 (dd, *J* = 22.5, 17.9 Hz, 1H), 1.43 (s, 9H).

^{13}C NMR (126 MHz, Chloroform-*d*) δ 194.7 (d, *J* = 18.5 Hz), 165.6 (d, *J* = 27.6 Hz), 154.0 (d, *J* = 3.9 Hz), 134.0, 132.8 (q, *J* = 3.4 Hz), 131.4 (q, *J* = 33.4 Hz), 127.3 (d, *J* = 1.4 Hz), 122.6 (q, *J* = 4.1 Hz), 94.2 (d, *J* = 203.4 Hz), 84.7, 38.3 (d, *J* = 24.7 Hz), 27.8.

HRMS (ESI) m/z calcd for $C_{15}H_{14}F_4KO_3^+$ $(M+K)^+$ 357.0511, found 357.0505.

***tert*-butyl 2-fluoro-5-methyl-1-oxo-2,3-dihydro-1*H*-indene-2-carboxylate (*rac*-3j)**

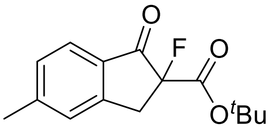

Using the typical procedure afforded racemic 3j as a yellow solid (chromatography on silica gel, *n*-hexane:ethyl acetate=60:1).

^{1}H NMR (400 MHz, Chloroform-*d*) δ 7.70 (d, *J* = 7.8 Hz, 1H), 7.30-7.21 (m, 2H), 3.66 (dd, *J* = 17.5, 10.9 Hz, 1H), 3.32 (dd, *J* = 22.9, 17.5 Hz, 1H), 2.46 (s, 3H), 1.42 (s, 9H).

^{13}C NMR (101 MHz, Chloroform-*d*) δ 195.1 (d, *J* = 18.3 Hz), 166.4 (d, *J* = 27.4 Hz), 151.5 (d, *J* = 3.9 Hz), 148.1, 131.3, 129.7, 126.8, 125.3, 94.7 (d, *J* = 201.3 Hz), 84.0, 38.2 (d, *J* = 24.1 Hz), 27.8, 22.3.

HRMS (ESI) m/z calcd for $C_{15}H_{17}FNaO_3^+$ $(M+Na)^+$ 287.1054, found 287.1046.

***tert*-butyl 5-chloro-2-fluoro-1-oxo-2,3-dihydro-1*H*-indene-2-carboxylate (*rac*-3k)**

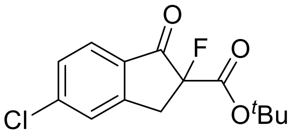

Using the typical procedure afforded racemic 3k as a white solid (chromatography on silica gel, *n*-hexane:ethyl acetate=60:1).

^{1}H NMR (500 MHz, Chloroform-*d*) δ 7.74 (d, *J* = 8.2 Hz, 1H), 7.48 (s, 1H), 7.44-7.38 (m, 1H), 3.69 (dd, *J* = 17.7, 10.7 Hz, 1H), 3.36 (dd, *J* = 22.6, 17.7 Hz, 1H), 1.42 (s, 9H).

^{13}C NMR (126 MHz, Chloroform-*d*) δ 194.3 (d, *J* = 18.5 Hz), 165.8 (d, *J* = 27.7 Hz), 152.3 (d, *J* = 4.0 Hz), 143.1, 132.0, 129.4, 126.8 (d, *J* = 1.2 Hz), 126.5, 94.2 (d, *J* = 202.8 Hz), 84.4, 38.0 (d, *J* = 24.4 Hz), 27.8.

HRMS (ESI) m/z calcd for $C_{14}H_{14}ClFNaO_3^+$ $(M+Na)^+$ 307.0508, found 307.0502.

***tert*-butyl 5-bromo-2-fluoro-1-oxo-2,3-dihydro-1*H*-indene-2-carboxylate (*rac*-3l)[23]**

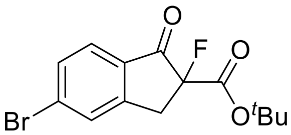

Using the typical procedure afforded racemic 3l as a yellow solid (chromatography on silica gel, *n*-hexane:ethyl acetate=60:1).

^{1}H NMR (500 MHz, Chloroform-*d*) δ 7.71-7.65 (m, 2H), 7.62-7.56 (m, 1H), 3.70 (dd, *J* = 17.7, 10.7 Hz, 1H), 3.37 (dd, *J* = 22.6, 17.8 Hz, 1H), 1.43 (s, 9H).

^{13}C NMR (126 MHz, Chloroform-*d*) δ 194.6 (d, *J* = 18.5 Hz), 165.8 (d, *J* = 27.7 Hz),

152.3 (d, J = 4.0 Hz), 132.4, 132.2, 132.0, 129.8 (d, J = 1.6 Hz), 126.5, 94.1 (d, J = 202.9 Hz), 84.5, 37.9 (d, J = 24.7 Hz), 27.8.

Ethyl 5-bromo-2-fluoro-1-oxo-2,3-dihydro-1*H*-indene-2-carboxylate (*rac*-3m)[24]

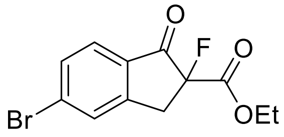

Using the typical procedure afforded racemic 3m as a yellow solid (chromatography on silica gel, *n*-hexane:ethyl acetate=60:1).

^{1}H NMR (400 MHz, Chloroform-*d*) δ 7.72-7.68 (m, 2H), 7.64-7.60 (m, 1H), 4.29 (q, J = 7.1 Hz, 2H), 3.78 (m, 1H), 3.42 (m, 1H), 1.27 (t, J = 7.1 Hz, 3H).

^{13}C NMR (101 MHz, Chloroform-*d*) δ 194.1 (d, J = 18.5 Hz), 166.9 (d, J = 27.5 Hz), 152.3 (d, J = 3.6 Hz), 132.4, 132.4, 132.1, 130.0, 126.7, 94.3 (d, J = 202.7 Hz), 62.8, 37.9 (d, J = 24.3 Hz), 14.0.

***tert*-butyl 2-fluoro-5-methoxy-1-oxo-2,3-dihydro-1*H*-indene-2-carboxylate (*rac*-3n)**

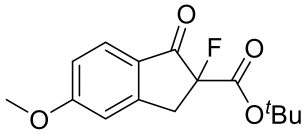

Using the typical procedure afforded racemic 3n as a white solid (chromatography on silica gel, *n*-hexane:ethyl acetate=60:1).

^{1}H NMR (400 MHz, Chloroform-d) δ 7.75 (d, J = 8.6 Hz, 1H), 6.95 (dd, J = 8.6, 2.2 Hz, 1H), 6.89 (d, J = 2.1 Hz, 1H), 3.91 (s, 3H), 3.66 (dd, J = 17.5, 10.8 Hz, 1H), 3.32 (dd, J = 22.7, 17.6 Hz, 1H), 1.43 (s, 9H).

^{13}C NMR (101 MHz, Chloroform-*d*) δ 193.6 (d, J = 18.5 Hz), 166.6, 154.1 (d, J = 4.0 Hz), 127.3, 126.7, 116.5, 109.7, 94.8 (d, J = 201.2 Hz), 84.0, 55.9, 38.3 (d, J = 24.4 Hz), 27.9.

HRMS (ESI) m/z calcd for $C_{15}H_{17}FNaO_4^+$ $(M+Na)^+$ 303.1003, found 303.0995.

***tert*-butyl 2-fluoro-5,6-dimethoxy-1-oxo-2,3-dihydro-1*H*-indene-2-carboxylate (*rac*-3o)**

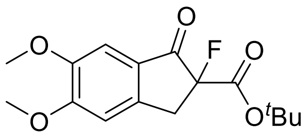

Using the typical procedure afforded racemic 3o as a yellow oil (chromatography on silica gel, *n*-hexane:ethyl acetate=50:1).

^{1}H NMR (400 MHz, Chloroform-*d*) δ 7.20 (s, 1H), 6.88 (s, 1H), 3.99 (s, 3H), 3.91 (s, 3H), 3.62 (dd, *J* = 17.2, 10.2 Hz, 1H), 3.36-3.21 (m, 1H), 1.44 (s, 9H).

^{13}C NMR (101 MHz, Chloroform-d) δ 194.1 (d, J = 18.7 Hz), 166.6 (d, J = 27.7 Hz), 156.9, 150.2, 147.0 (d, J = 4.1 Hz), 126.3, 107.2, 105.4, 94.8 (d, J = 201.6 Hz), 83.9, 56.4, 56.2, 38.1 (d, J = 24.4 Hz), 27.9.

HRMS (ESI) m/z calcd for $C_{16}H_{19}FNaO_5^+$ $(M+Na)^+$ 333.1109, found 333.1102.

***tert*-butyl 4-bromo-2-fluoro-1-oxo-2,3-dihydro-1*H*-indene-2-carboxylate (*rac*-3p)[25]**

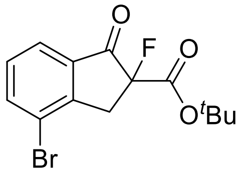

Using the typical procedure afforded racemic 3p as a yellow oil (chromatography on silica gel, *n*-hexane:ethyl acetate=60:1).

^{1}H NMR (500 MHz, Chloroform-*d*) δ 7.85 (dd, *J* = 7.8, 1.0 Hz, 1H), 7.78 (d, *J* = 7.6 Hz, 1H), 7.37 (t, *J* = 7.7 Hz, 1H), 3.66 (dd, *J* = 18.0, 11.2 Hz, 1H), 3.32 (dd, *J* = 22.7, 18.0 Hz, 1H), 1.45 (s, 9H).

^{13}C NMR (126 MHz, Chloroform-*d*) δ 195.2 (d, *J* = 18.5 Hz), 165.8 (d, *J* = 27.2 Hz), 150.8 (d, *J* = 4.0 Hz), 139.1, 135.5, 130.2, 124.2, 121.7, 93.9 (d, *J* = 203.1 Hz), 84.6, 39.4 (d, *J* = 24.9 Hz), 27.8.

***tert*-butyl 7-bromo-2-fluoro-1-oxo-2,3-dihydro-1*H*-indene-2-carboxylate (*rac*-3q)[26]**

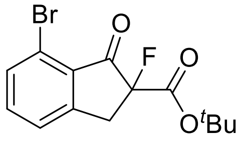

Using the typical procedure afforded racemic 3q as a yellow oil (chromatography on silica gel, *n*-hexane:ethyl acetate=60:1).

^{1}H NMR (400 MHz, Chloroform-*d*) δ 7.60 (d, *J* = 7.6 Hz, 1H), 7.49 (t, *J* = 7.6 Hz, 1H), 7.43 (dd, *J* = 7.5, 1.0 Hz, 1H), 3.68 (m, 1H), 3.35 (m, 1H), 1.43 (s, 9H).

^{13}C NMR (101 MHz, Chloroform-*d*) δ 193.0 (d, J = 19.1 Hz), 165.8 (d, J = 27.6 Hz), 153.4 (d, J = 3.8 Hz), 136.6, 133.4, 131.4 (d, J = 1.4 Hz), 125.3 (d, J = 1.4 Hz), 121.3, 94.5 (d, J = 202.5 Hz), 84.5, 37.4 (d, J = 24.1 Hz), 27.8.

***tert*-butyl 2-fluoro-1-oxo-1,2,3,4-tetrahydronaphthalene-2-carboxylate (*rac*-3r)**

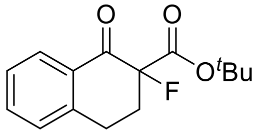

Using the typical procedure afforded racemic 3r as a white soild (chromatography on silica gel, *n*-hexane:ethyl acetate=50:1).

^{1}H NMR (400 MHz, Chloroform-d) δ 8.06 (d, J = 7.9 Hz, 1H), 7.53 (td, J = 7.5, 1.4 Hz, 1H), 7.35 (t, J = 7.6 Hz, 1H), 7.27 (d, J = 7.6 Hz, 1H), 3.21-3.02 (m, 2H), 2.75-2.62 (m, 1H), 2.56-2.44 (m, 1H), 1.43 (s, 9H).

^{13}C NMR (101 MHz, Chloroform-*d*) δ 189.3 (d, J = 18.5 Hz), 166.3 (d, J = 26.3 Hz), 142.9, 134.3, 131.1, 128.7, 128.2, 127.2, 93.1 (d, J = 193.9 Hz), 84.0, 31.9 (d, J = 22.3 Hz), 27.8, 25.2 (d, J = 7.8 Hz).

HRMS (ESI) m/z calcd for $C_{15}H_{17}FNaO_3^+$ $(M+Na)^+$ 287.1054, found 287.1048.

methyl 2-fluoro-1-oxo-1,2,3,4-tetrahydronaphthalene-2-carboxylate (*rac*-3s)

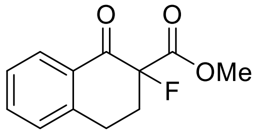

Using the typical procedure afforded racemic 3s as a yellow soild (chromatography on silica gel, *n*-hexane:ethyl acetate=60:1).

^{1}H NMR (400 MHz, Chloroform-*d*) δ 8.07 (dd, J = 7.9, 1.4 Hz, 1H), 7.55 (td, J = 7.5, 1.5 Hz, 1H), 7.43-7.33 (m, 1H), 7.30-7.26 (m, 1H), 3.82 (s, 3H), 3.28-3.13 (m, 1H), 3.13-3.01 (m, 1H), 2.83-2.63 (m, 1H), 2.62-2.46 (m, 1H).

^{13}C NMR (101 MHz, Chloroform-*d*) δ 188.5 (d, J = 18.6 Hz), 167.8 (d, J = 25.9 Hz), 143.2, 134.6, 130.5, 128.8, 128.5, 127.3, 93.3 (d, J = 193.9 Hz), 53.1, 31.9 (d, J = 22.2 Hz), 24.9 (d, J = 7.2 Hz).

HRMS (ESI) m/z calcd for $C_{12}H_{11}FNaO_3^+$ $(M+Na)^+$ 245.0584, found 245.0581.

tert-butyl 6-fluoro-5-oxo-6,7,8,9-tetrahydro-5H-benzo[7]annulene-6-carboxylate (rac-3t)

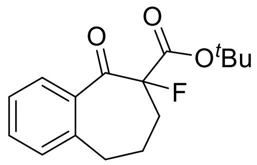

Using the typical procedure afforded racemic 3t as a colorless oil (chromatography on silica gel, *n*-hexane:ethyl acetate=60:1).

^{1}H NMR (500 MHz, Chloroform-*d*) δ 7.52 (dd, *J* = 7.7, 1.5 Hz, 1H), 7.42 (td, *J* = 7.5, 1.5 Hz, 1H), 7.30 (t, *J* = 7.3 Hz, 1H), 7.19 (d, *J* = 7.6 Hz, 1H), 3.10-3.00 (m, 1H), 2.98-2.88 (m, 1H), 2.62-2.47 (m, 1H), 2.29-2.16 (m, 1H), 2.15-2.02 (m, 1H), 1.99-1.87 (m, 1H), 1.41 (s, 9H).

^{13}C NMR (126 MHz, Chloroform-*d*) δ 199.2 (d, *J* = 24.2 Hz), 166.0 (d, *J* = 25.2 Hz), 140.2, 137.1, 132.1, 129.5, 129.2, 126.6, 98.4 (d, *J* = 194.9 Hz), 83.6, 33.3, 32.6 (d, *J* = 22.4 Hz), 27.7, 22.3 (d, *J* = 4.0 Hz).

HRMS (ESI) m/z calcd for $C_{16}H_{19}FNaO_3^+$ $(M+Na)^+$ 301.1210, found 301.1207.

tert-butyl 3-fluoro-3-methyl-2-oxoindoline-1-carboxylate (rac-3u)[27]

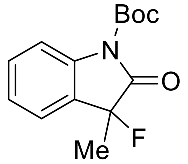

Using the typical procedure afforded racemic 3u as a colorless oil (chromatography on silica gel, *n*-hexane:ethyl acetate=40:1).

^{1}H NMR (500 MHz, Chloroform-*d*) δ 7.92 (d, *J* = 8.2 Hz, 1H), 7.52 – 7.42 (m, 2H), 7.31 – 7.22 (m, 1H), 1.81 (d, *J* = 21.6 Hz, 3H), 1.67 (s, 9H).

^{13}C NMR (126 MHz, Chloroform-*d*) δ 171.3 (d, *J* = 21.6 Hz), 148.8, 139.7 (d, *J* = 4.9 Hz), 131.4 (d, *J* = 3.0 Hz), 126.2 (d, *J* = 18.6 Hz), 125.1 (d, *J* = 2.8 Hz), 124.1, 115.6, 90.3 (d, *J* = 184.1 Hz), 85.0, 28.1, 21.9 (d, *J* = 30.1 Hz).

tert-butyl 2-fluoro-3-oxo-3-phenylpropanoate (rac-3v)[28]

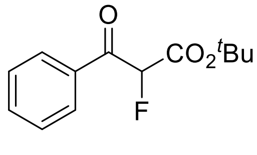

Using the typical procedure afforded racemic 3v as a colorless oil (chromatography on silica gel, *n*-hexane:ethyl acetate=60:1).

^{1}H NMR (400 MHz, Chloroform-*d*) δ 8.03 (m, *J* = 8.5, 1.2 Hz, 2H), 7.66 – 7.60 (m, 1H), 7.53 – 7.47 (m, 2H), 5.75 (d, *J* = 49.1 Hz, 1H), 1.43 (s, 9H).

^{13}C NMR (101 MHz, Chloroform-*d*) δ 189.9 (d, *J* = 20.0 Hz), 163.8 (d, *J* = 24.4 Hz), 134.3, 133.6 (d, *J* = 2.0 Hz), 129.4 (d, *J* = 3.2 Hz), 128.7, 90.1 (d, *J* = 196.8 Hz), 84.5, 27.8.

7.4 NMR and HPLC Spectra

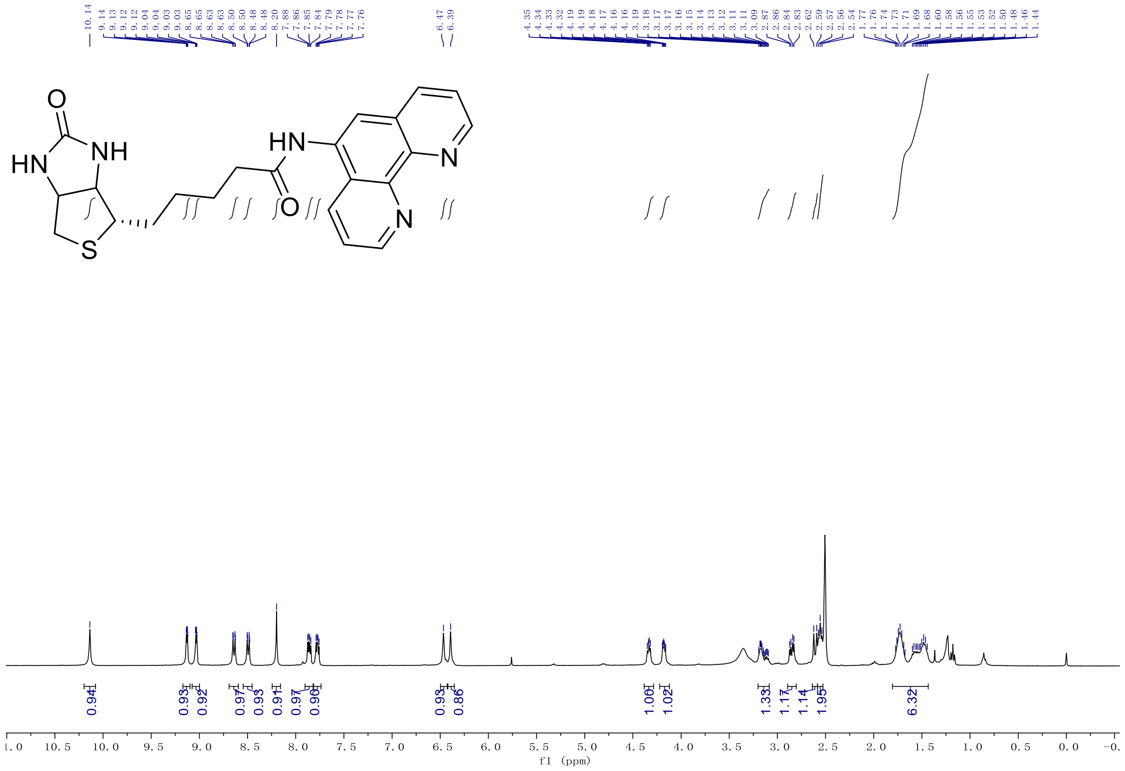

^{1}H NMR (DMSO-d_6) of **L1**

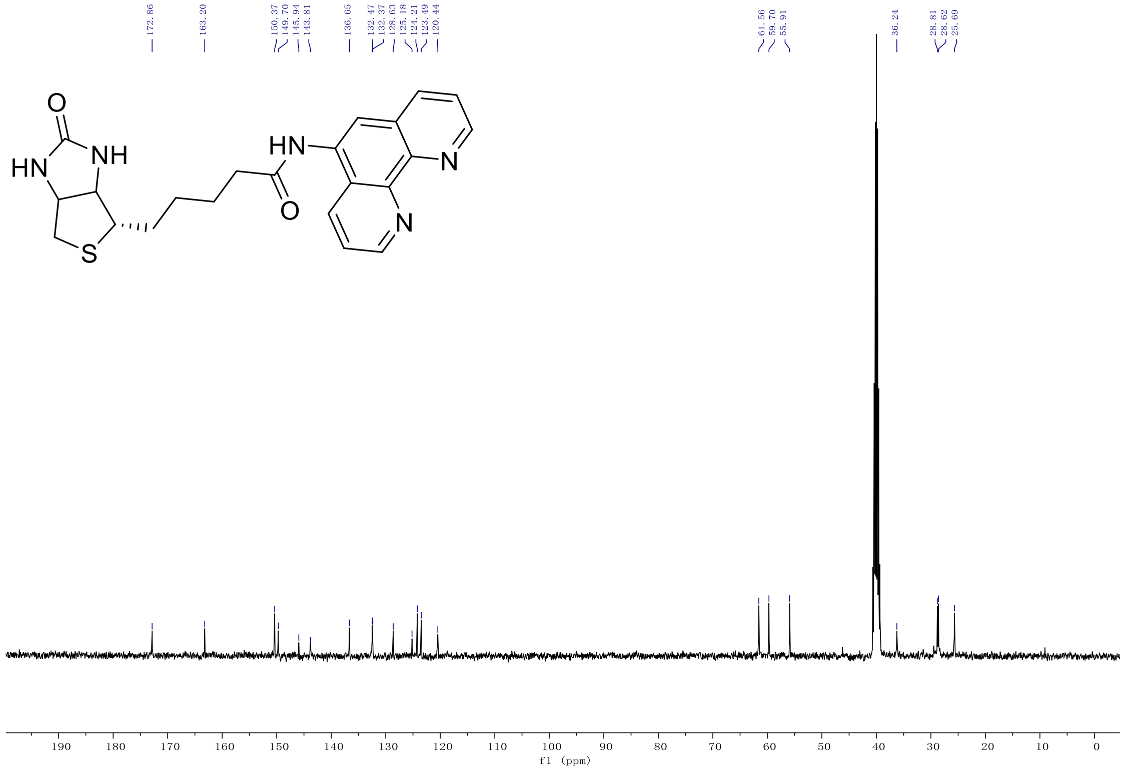

^{13}C NMR (DMSO-d_6) of **L1**

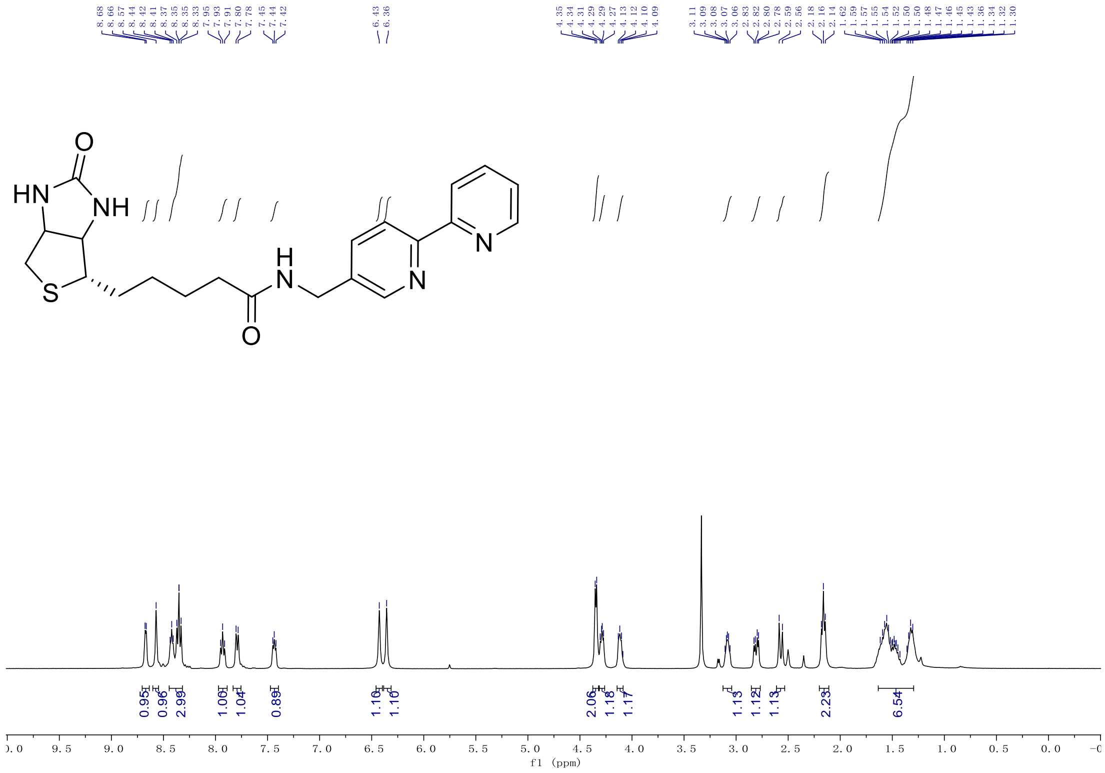

^{1}H NMR (DMSO-d_6) of **L2**

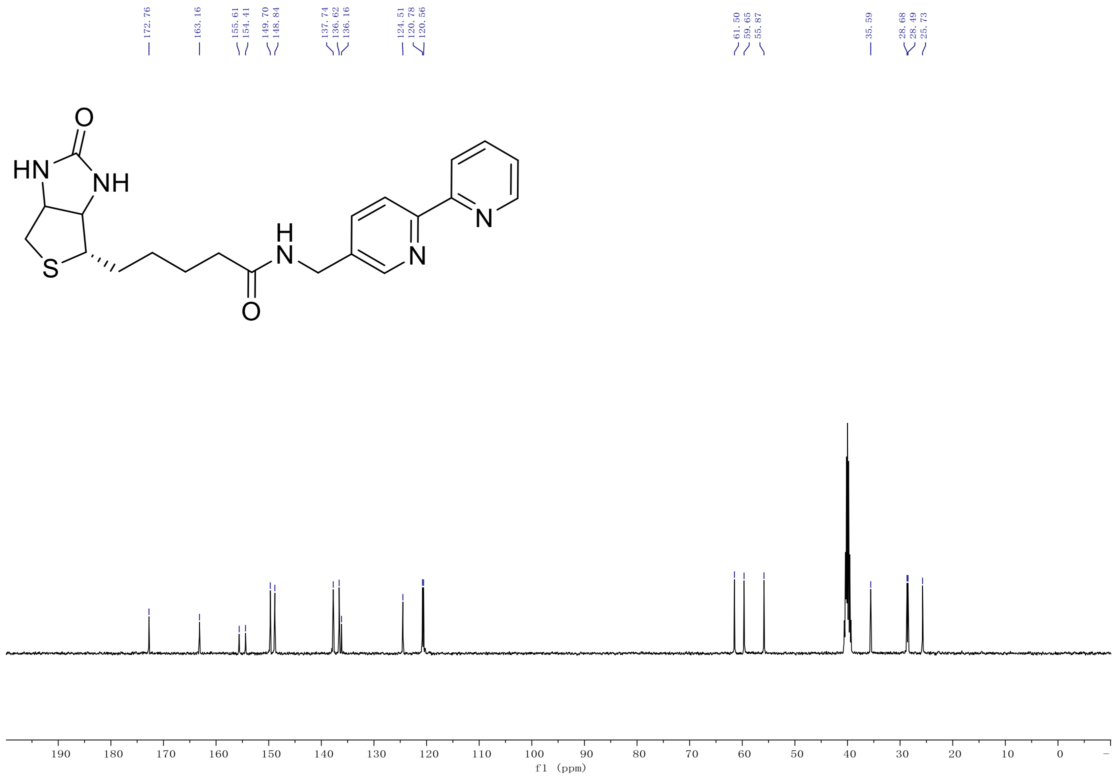

^{1}H NMR (DMSO-d_6) of **L2**

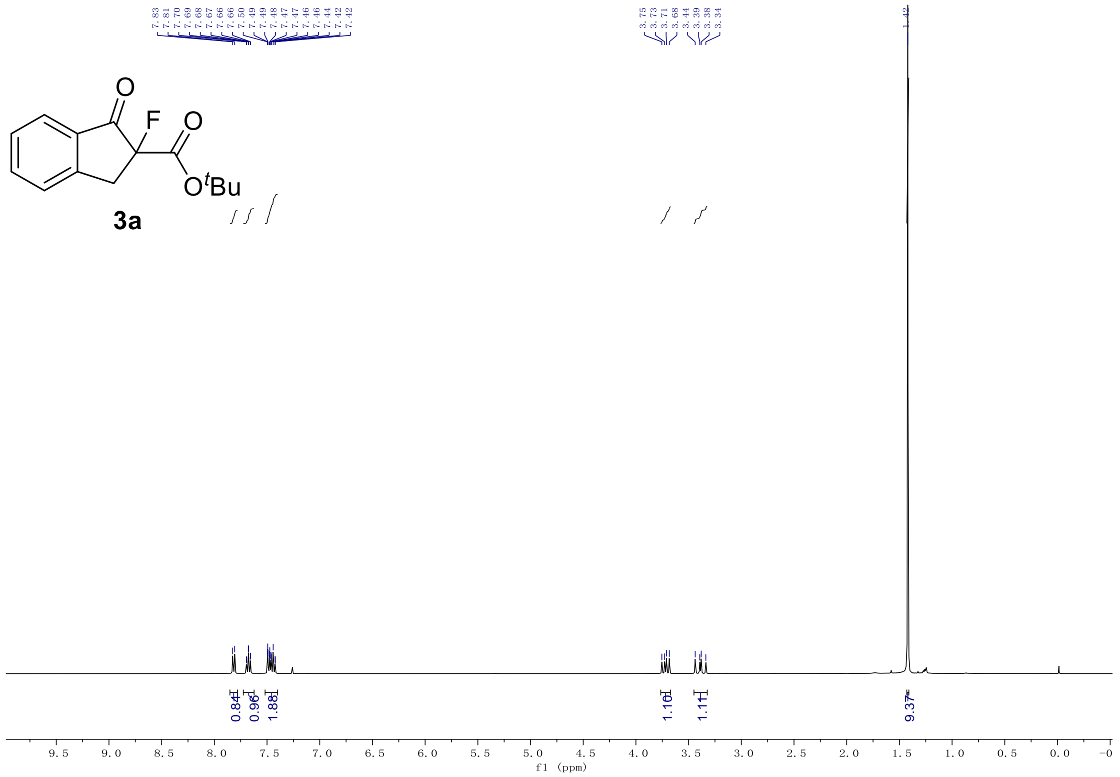

^{1}H NMR ($CDCl_3$) of **3a**

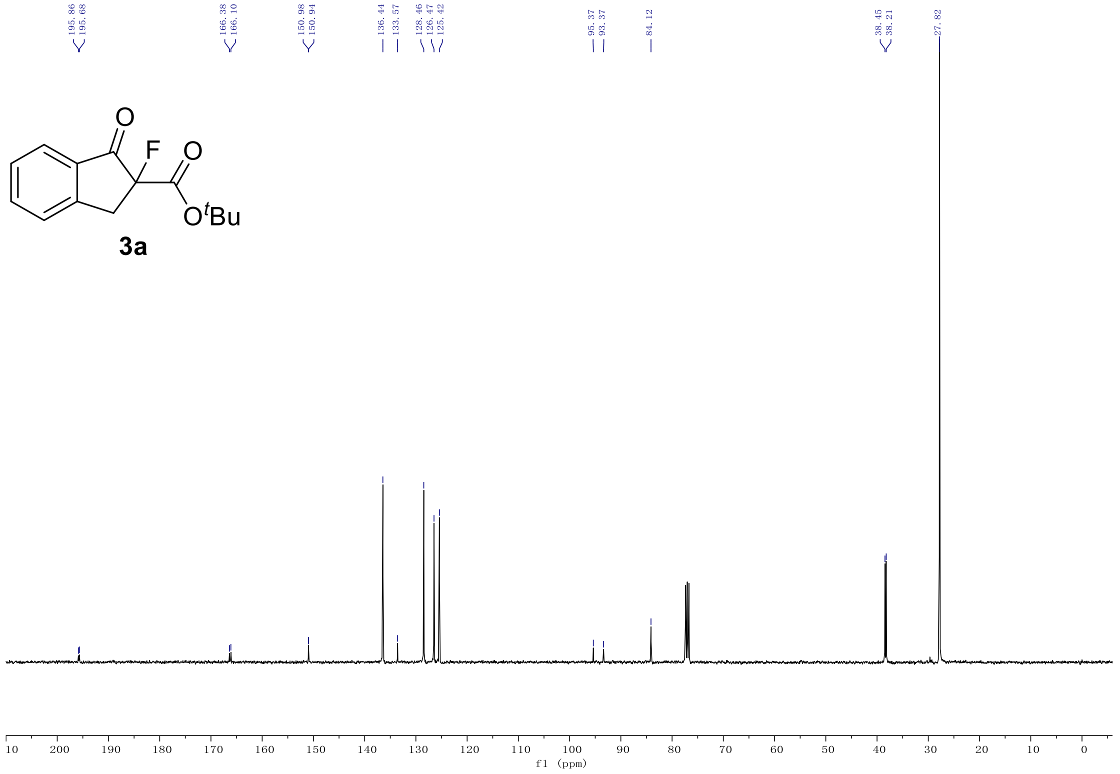

^{13}C NMR ($CDCl_3$) of **3a**

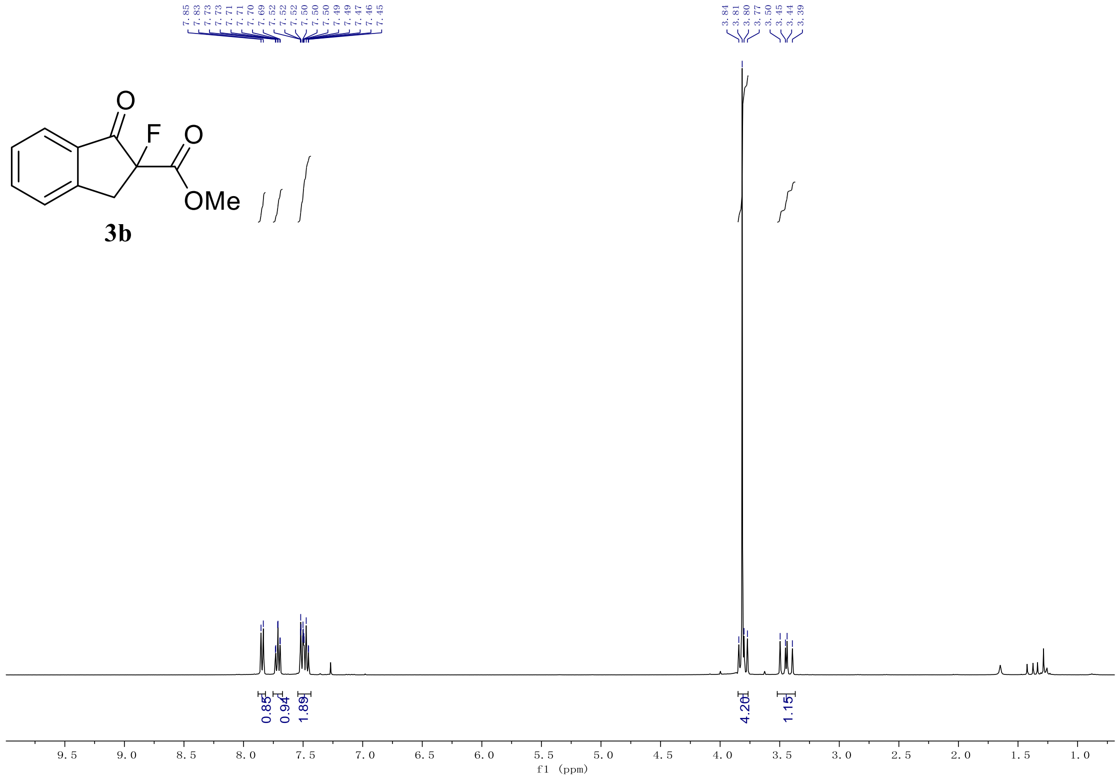

^{1}H NMR (CDCl$_3$) of **3b**

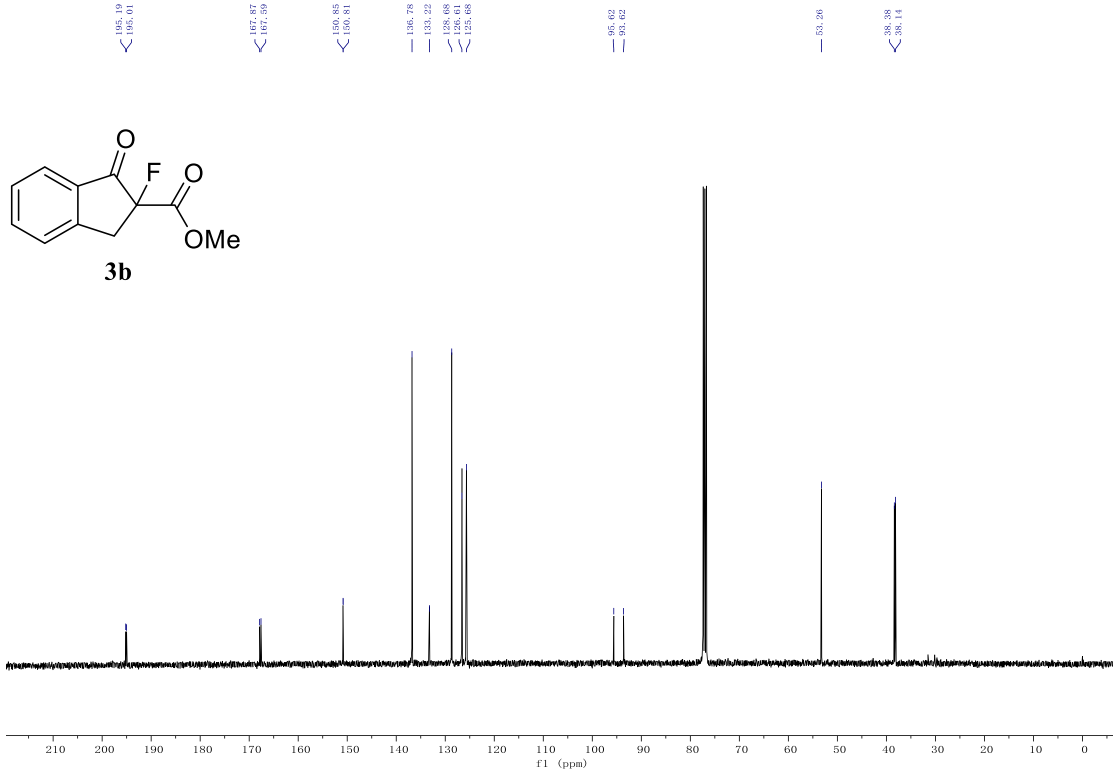

^{13}C NMR (CDCl$_3$) of **3b**

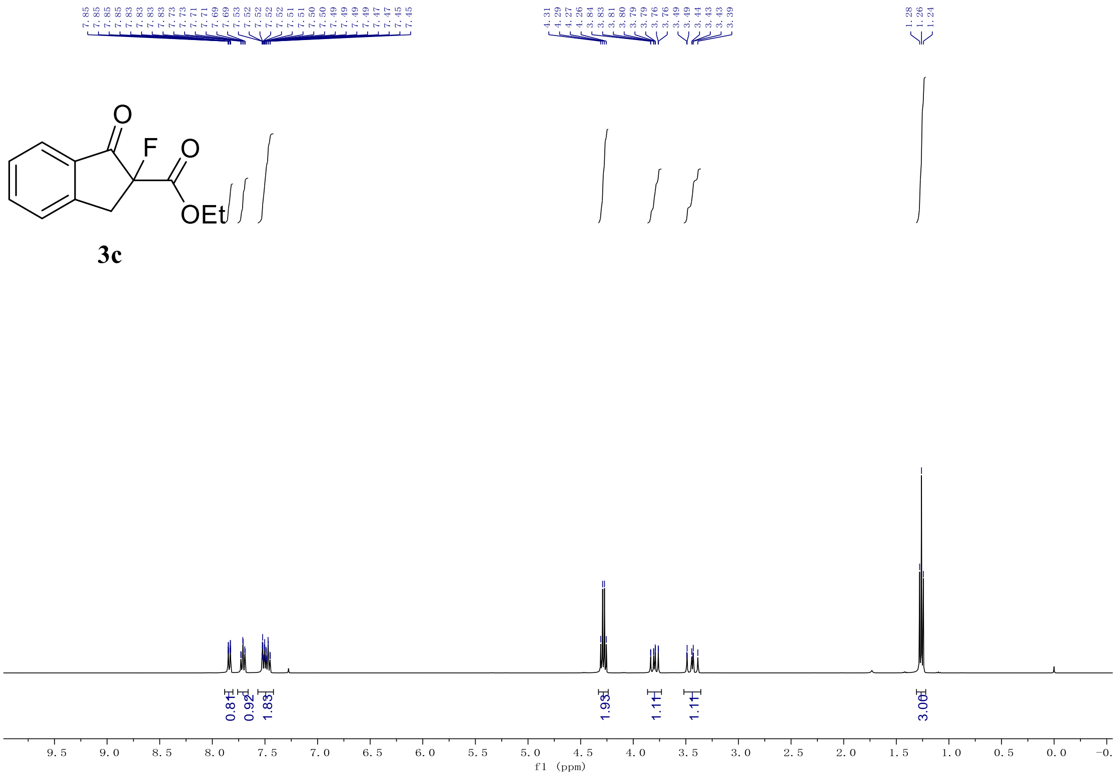

^{1}H NMR ($CDCl_3$) of **3c**

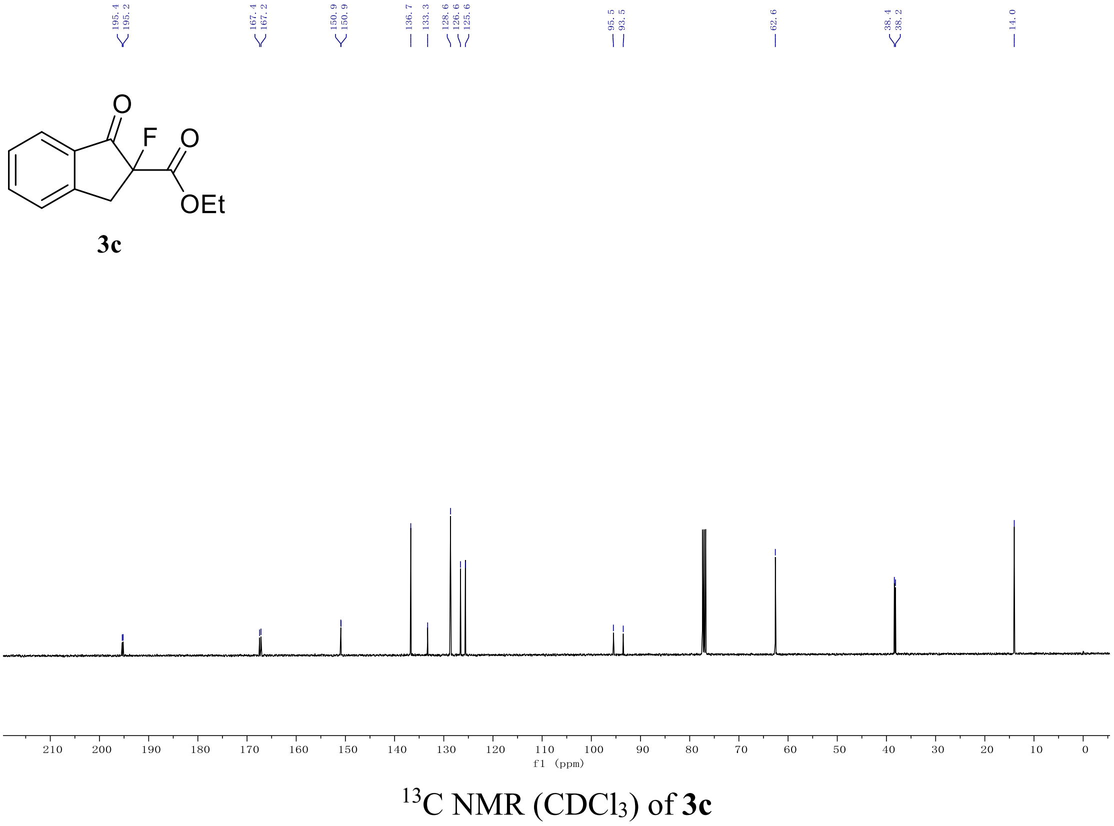

^{13}C NMR ($CDCl_3$) of **3c**

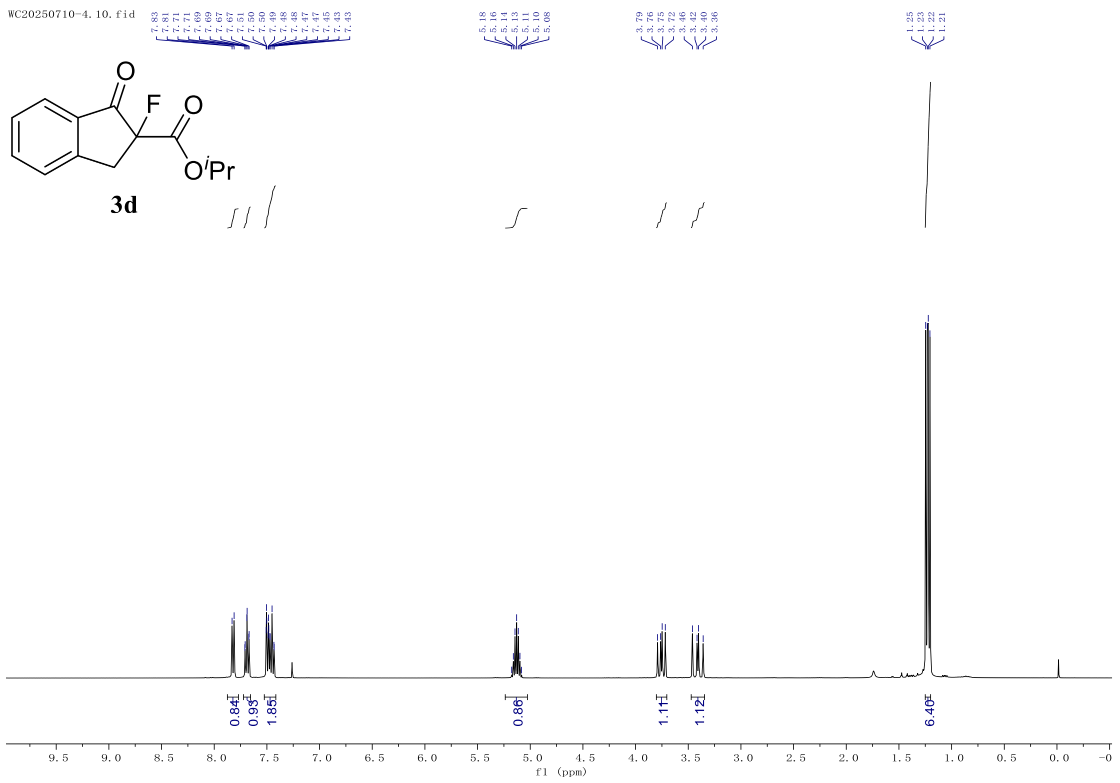

^{1}H NMR ($CDCl_3$) of **3d**

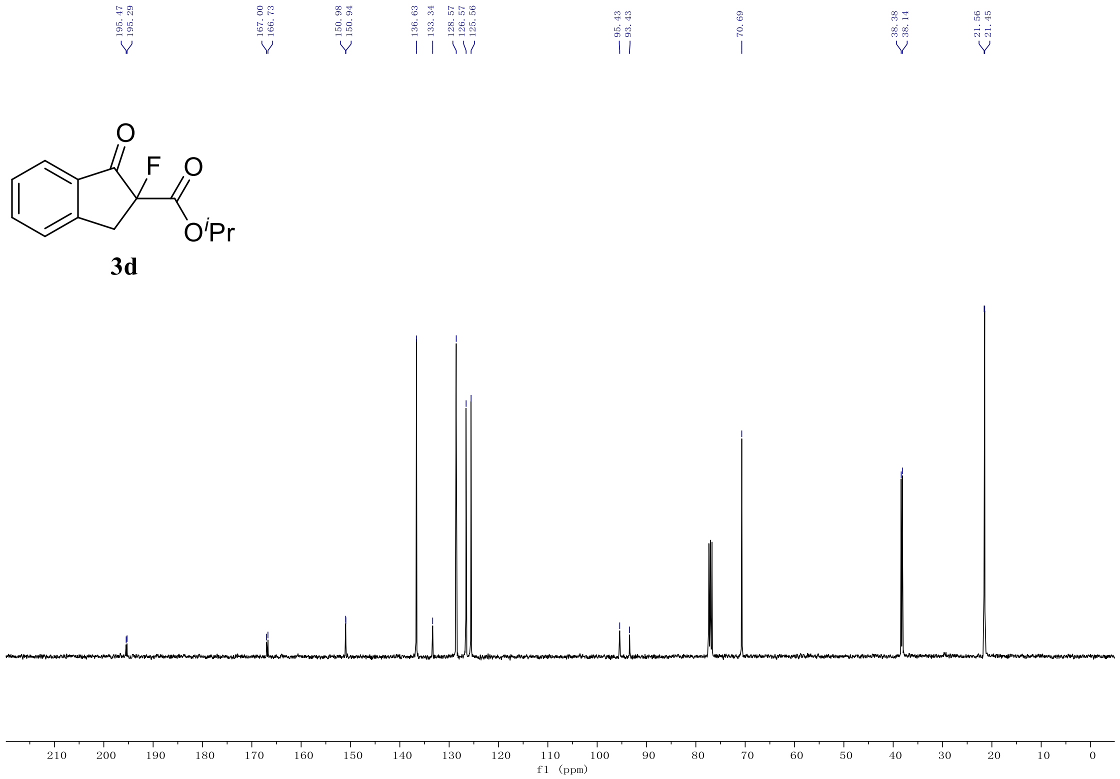

^{13}C NMR ($CDCl_3$) of **3d**

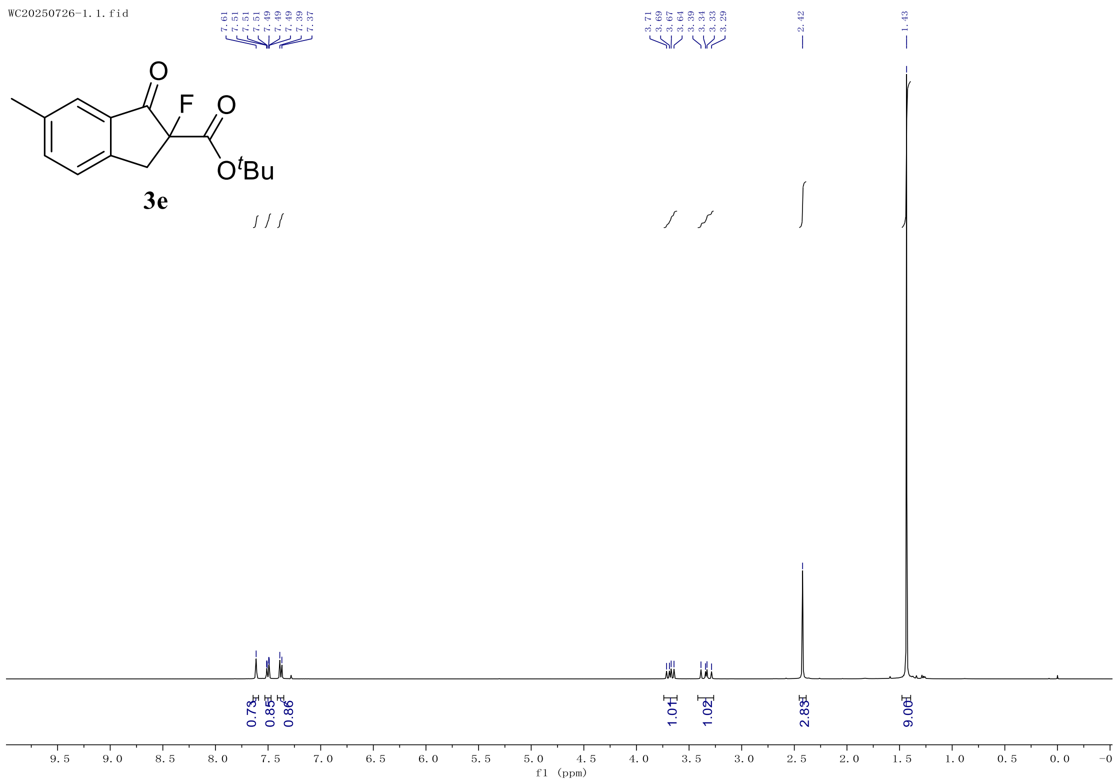

^{1}H NMR ($CDCl_3$) of **3e**

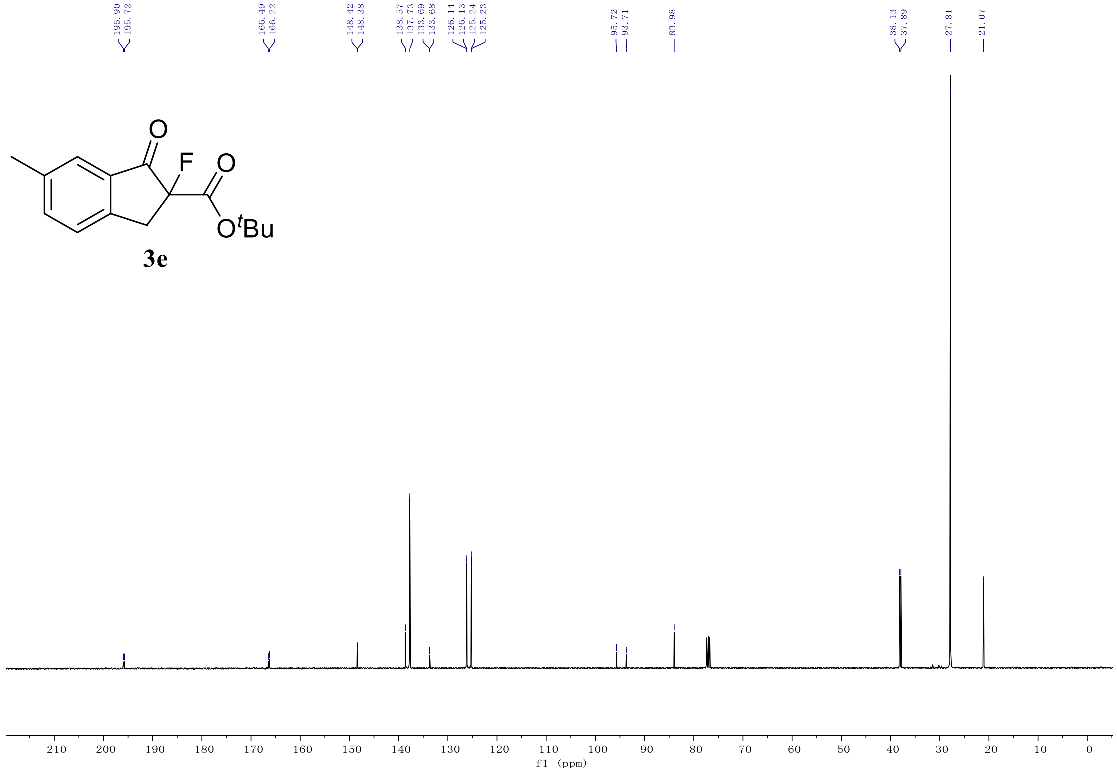

^{13}C NMR ($CDCl_3$) of **3e**

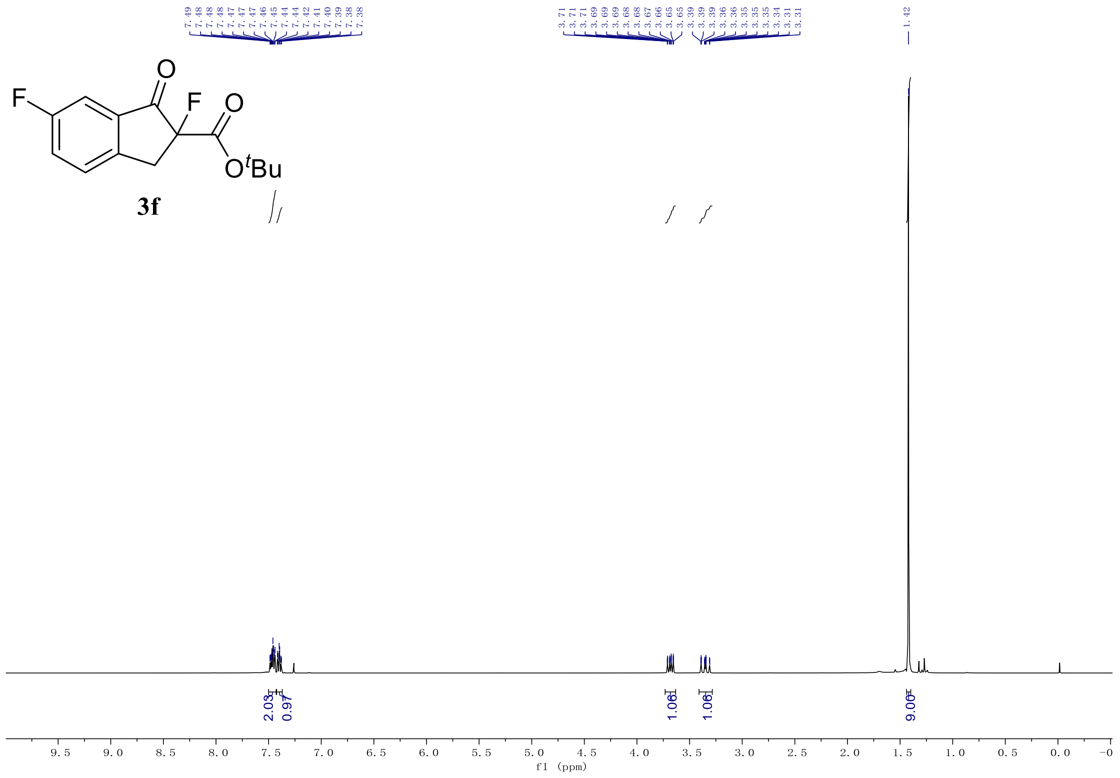

^{1}H NMR (CDCl$_3$) of **3f**

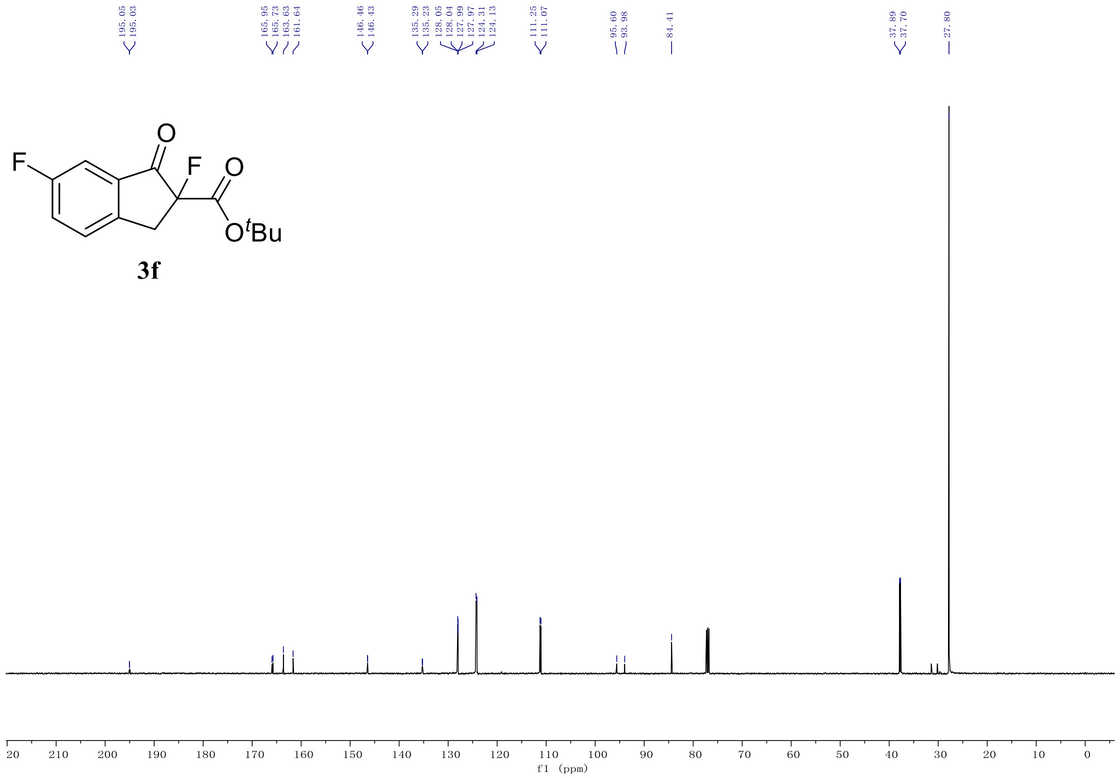

^{13}C NMR (CDCl$_3$) of **3f**

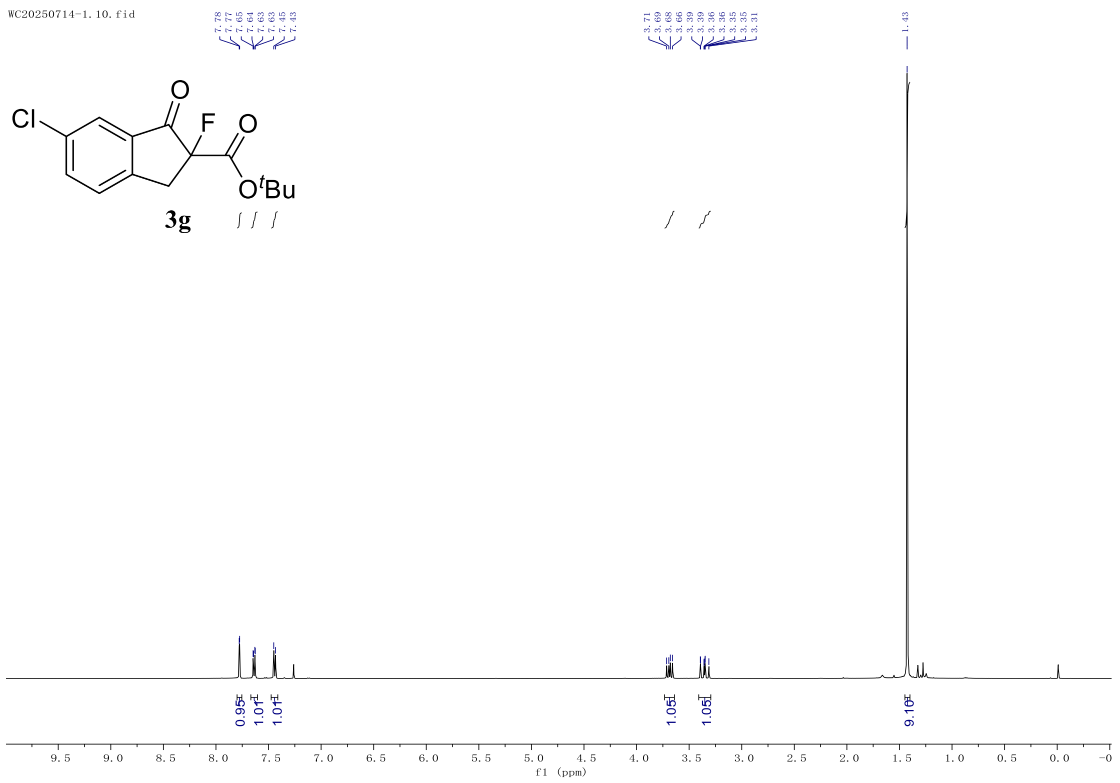

^{1}H NMR ($CDCl_3$) of **3g**

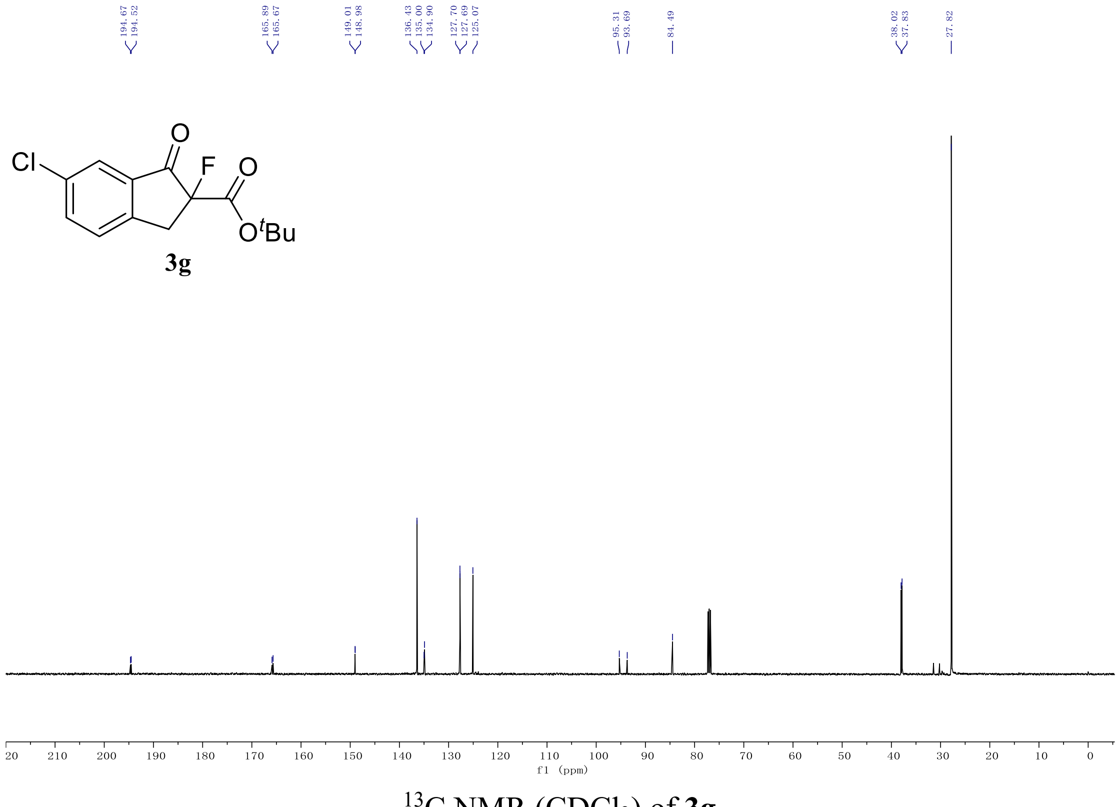

^{13}C NMR ($CDCl_3$) of **3g**

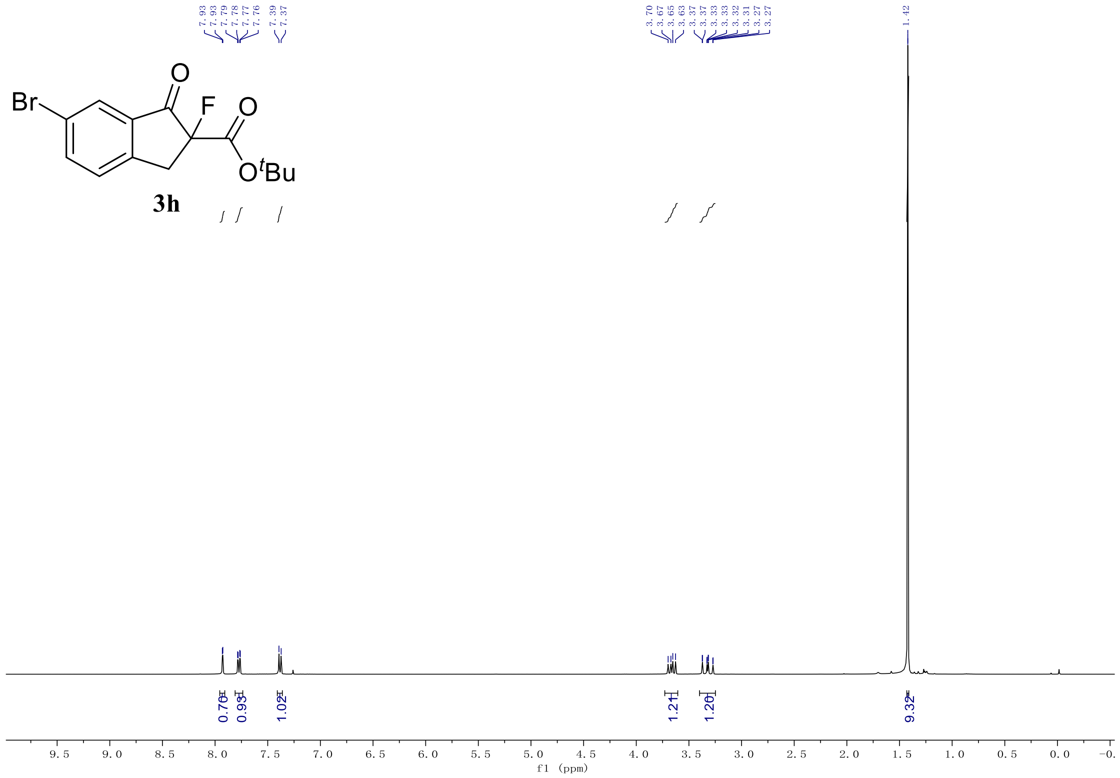

^{1}H NMR (CDCl$_3$) of **3h**

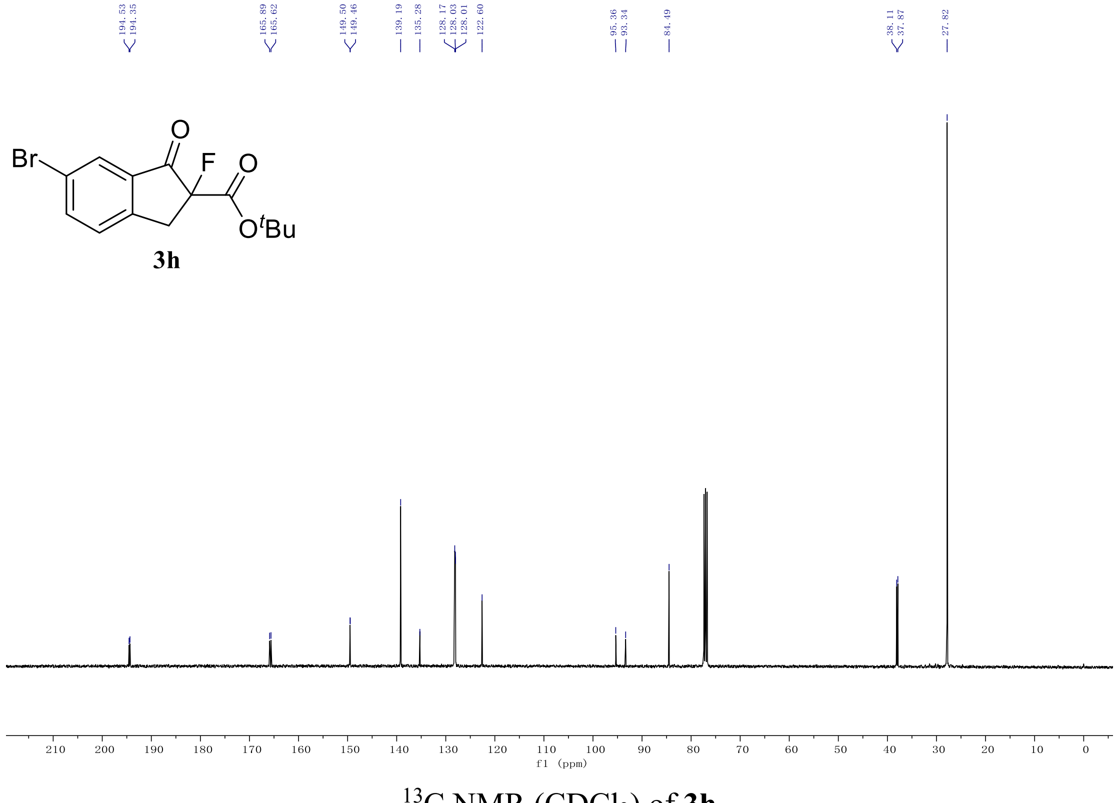

^{13}C NMR (CDCl$_3$) of **3h**

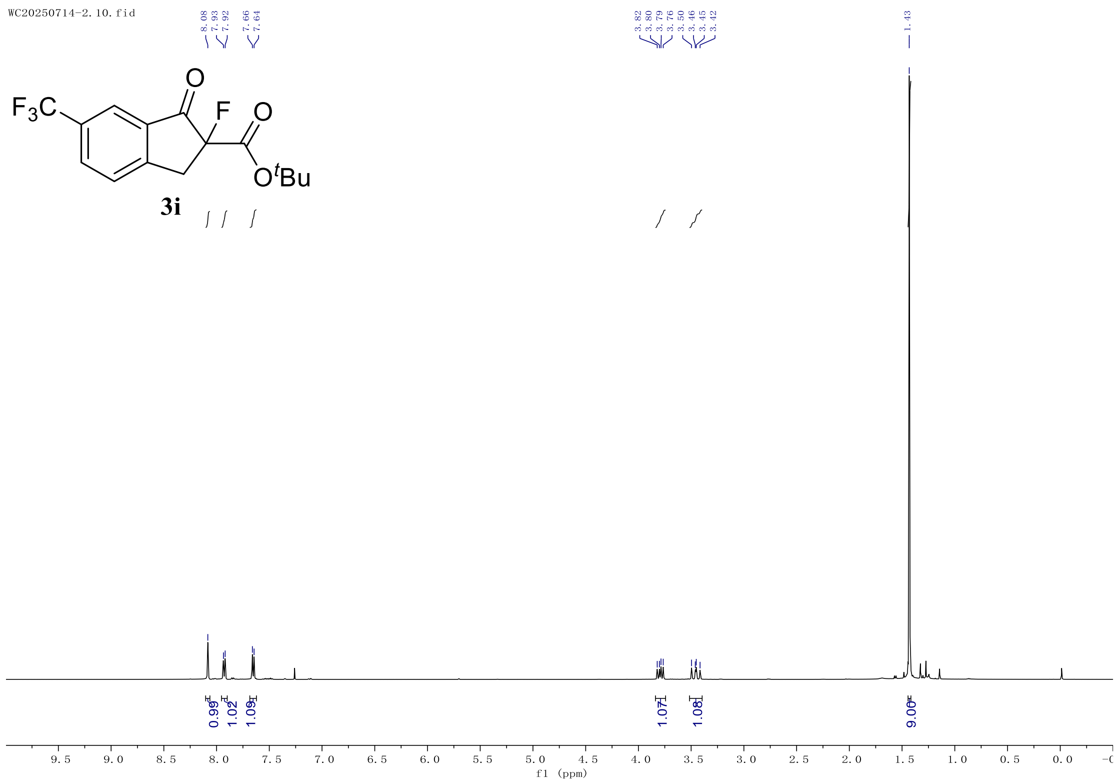

^{1}H NMR ($CDCl_3$) of **3i**

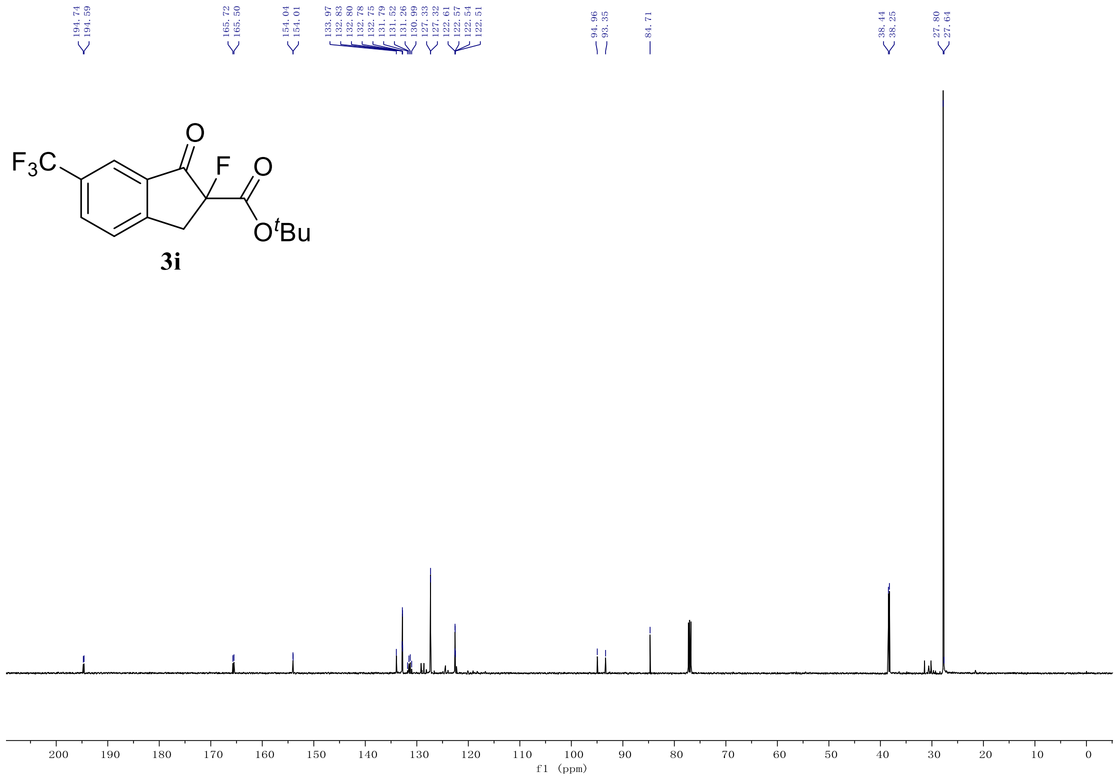

^{13}C NMR ($CDCl_3$) of **3i**

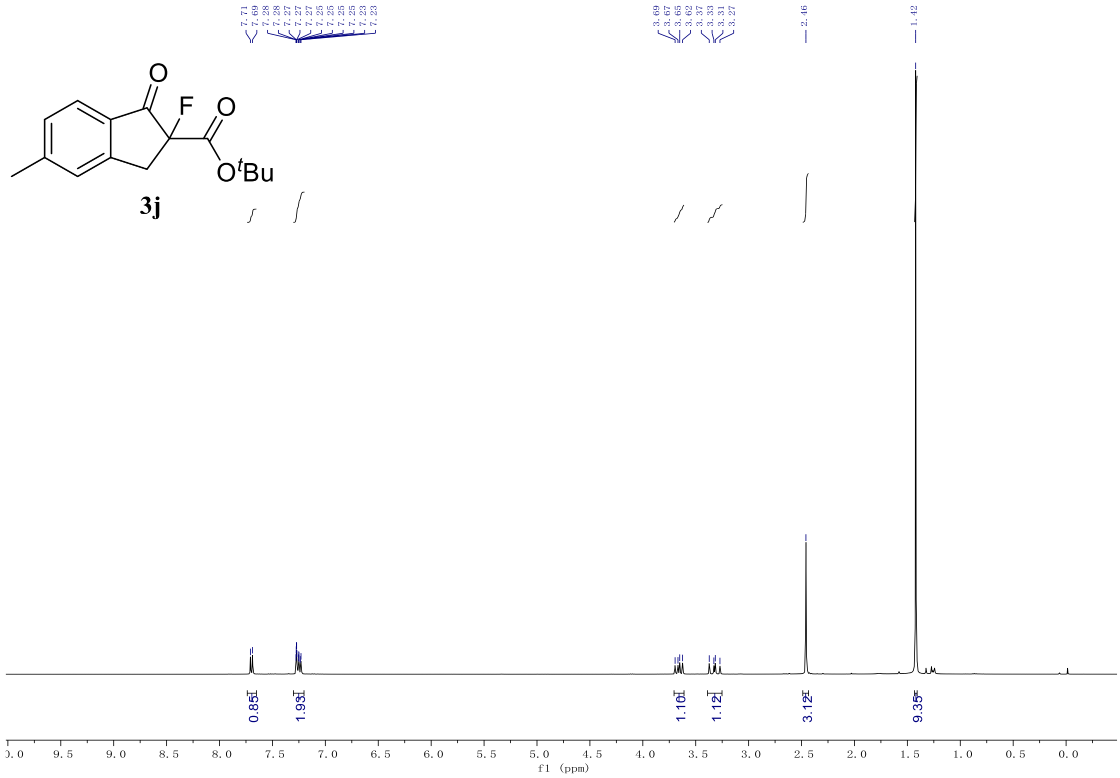

^{1}H NMR (CDCl$_3$) of **3j**

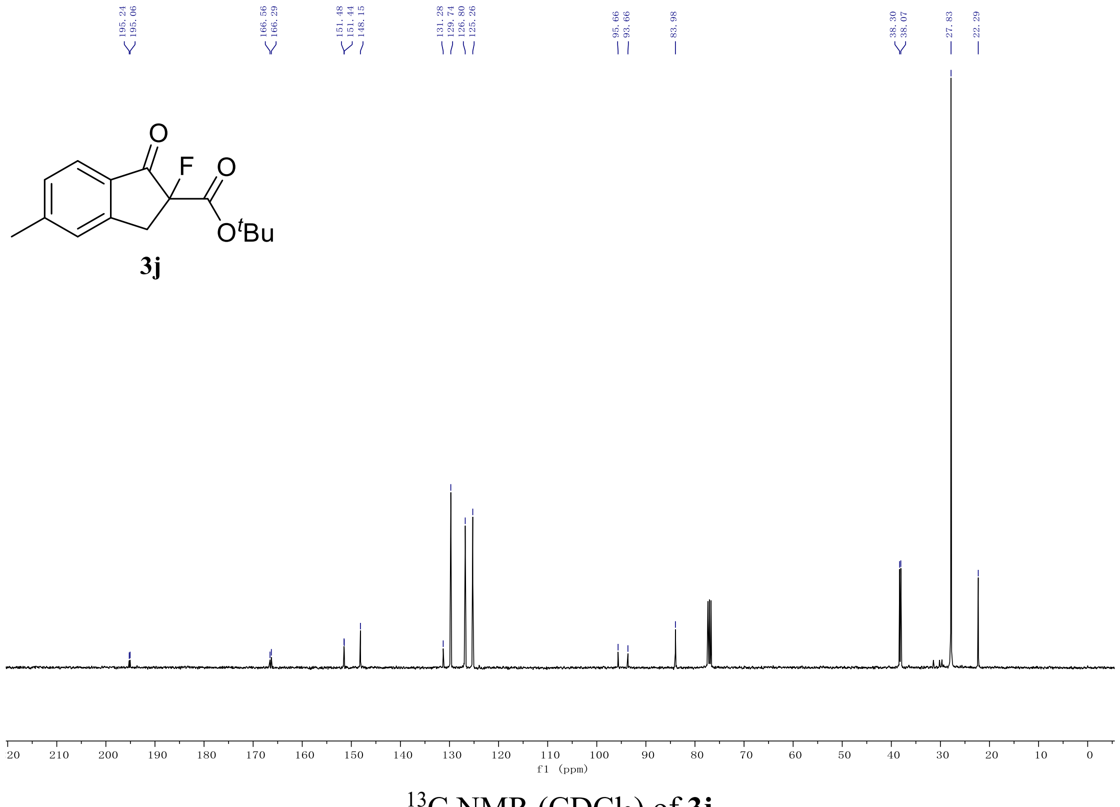

^{13}C NMR (CDCl$_3$) of **3j**

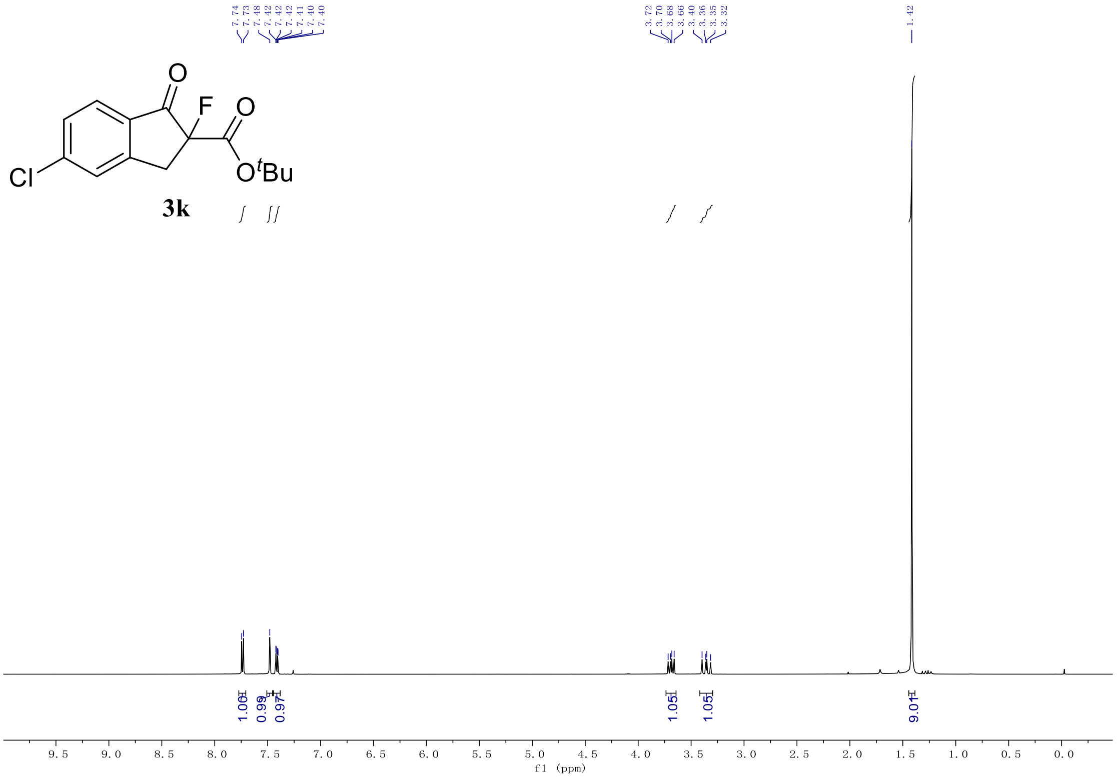

^{1}H NMR ($CDCl_3$) of **3k**

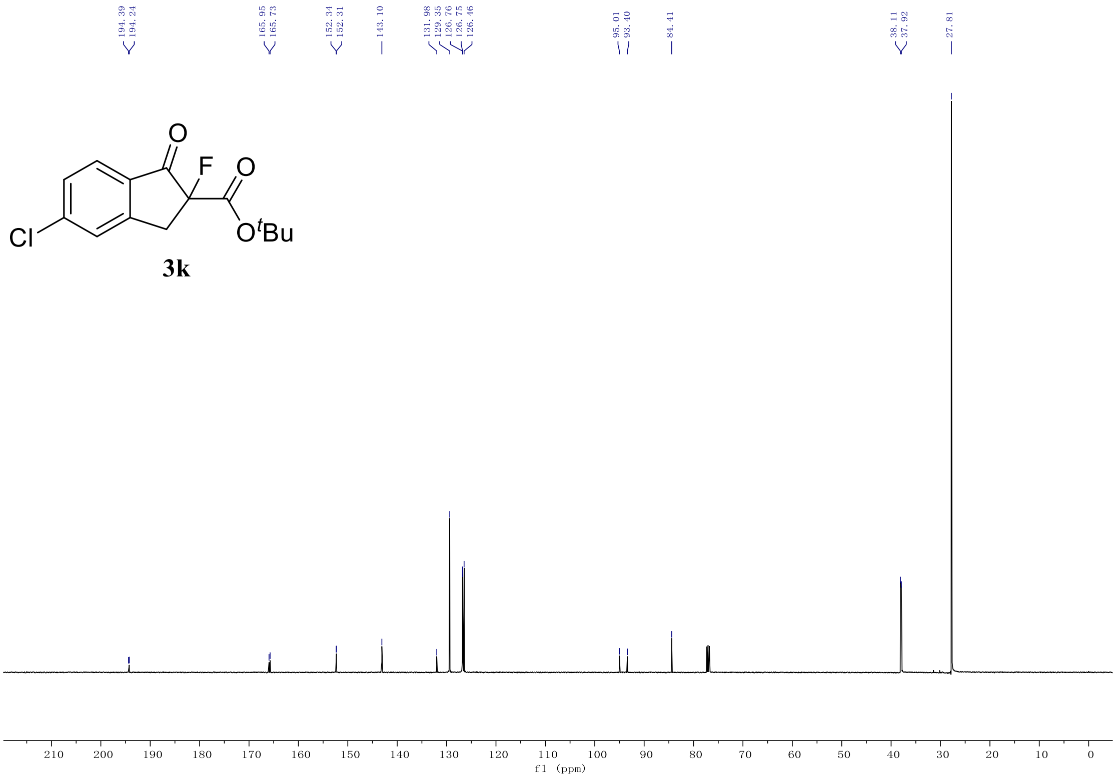

^{13}C NMR ($CDCl_3$) of **3k**

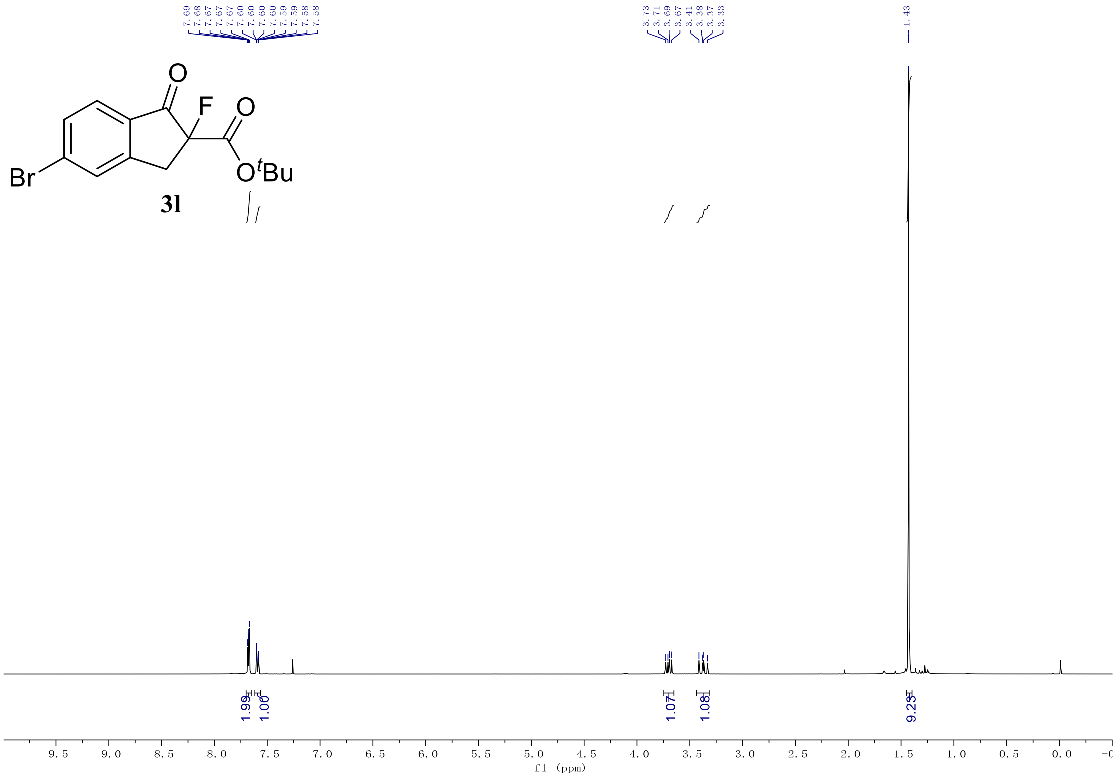

^{1}H NMR ($CDCl_3$) of **3l**

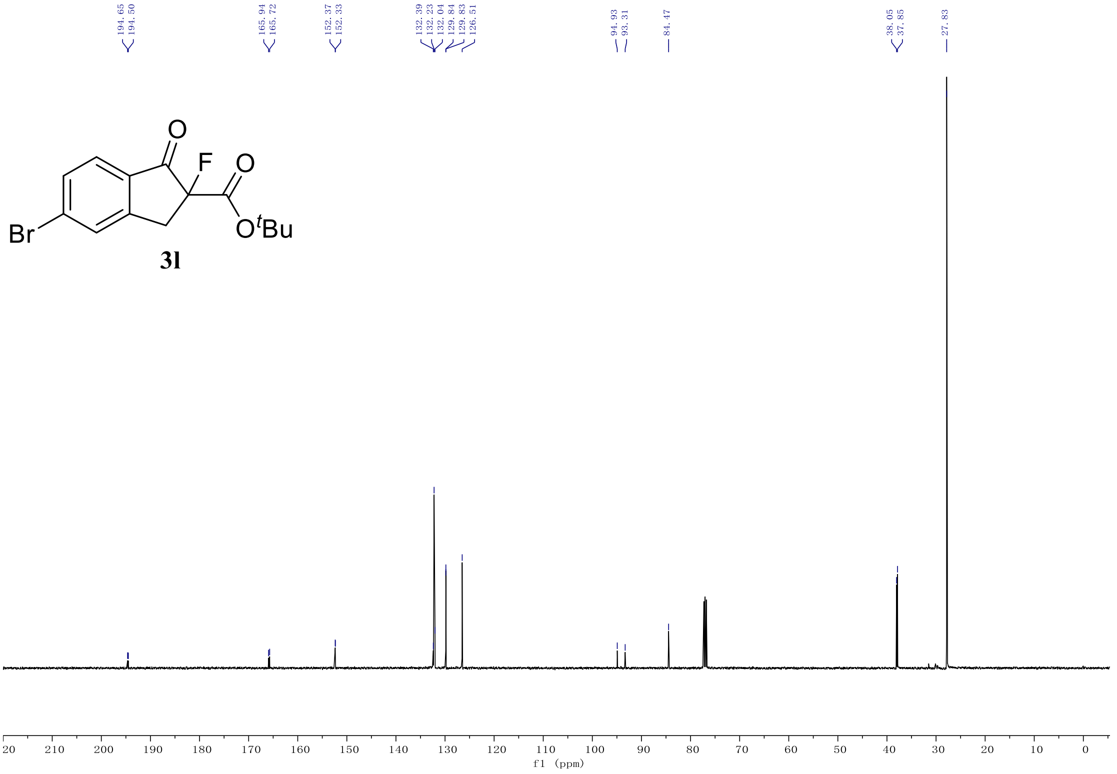

^{13}C NMR ($CDCl_3$) of **3l**

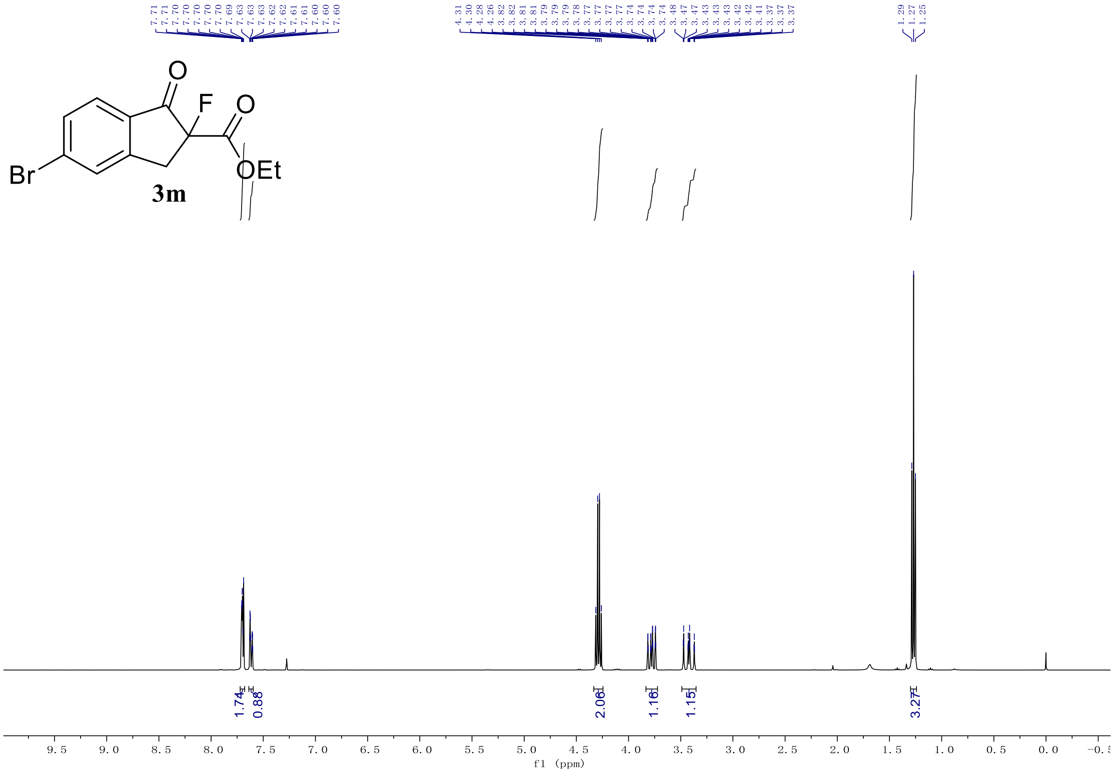

^{1}H NMR (CDCl$_3$) of **3m**

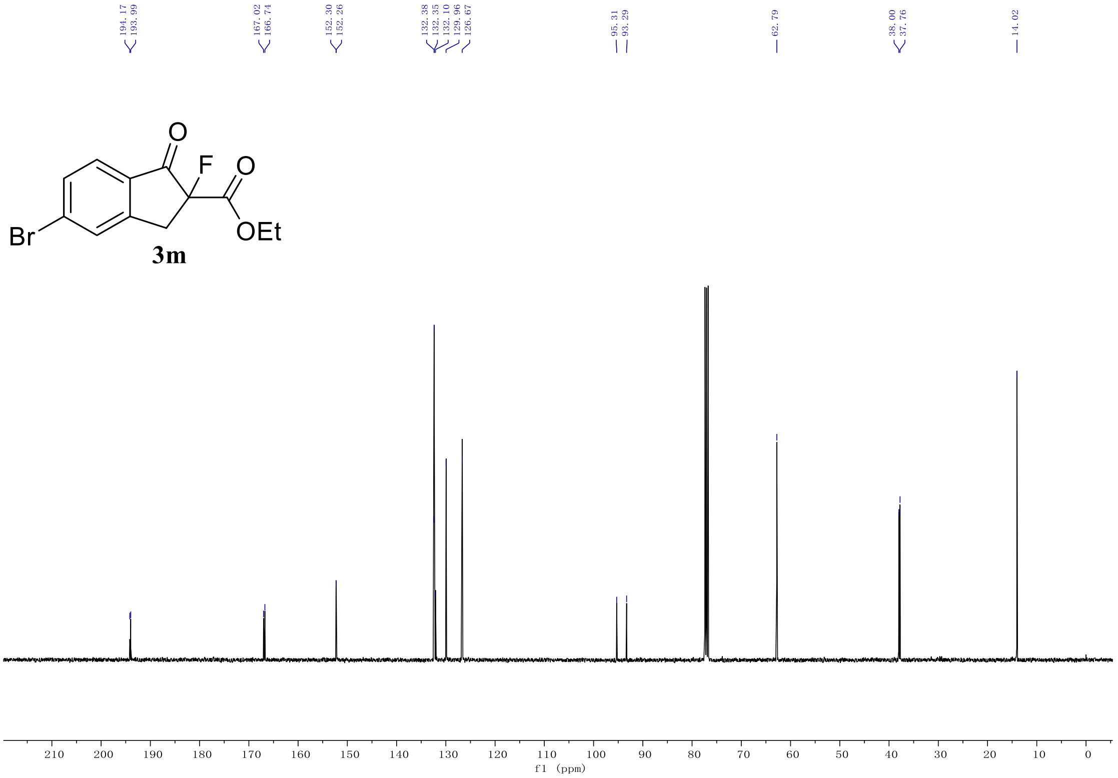

^{13}C NMR (CDCl$_3$) of **3m**

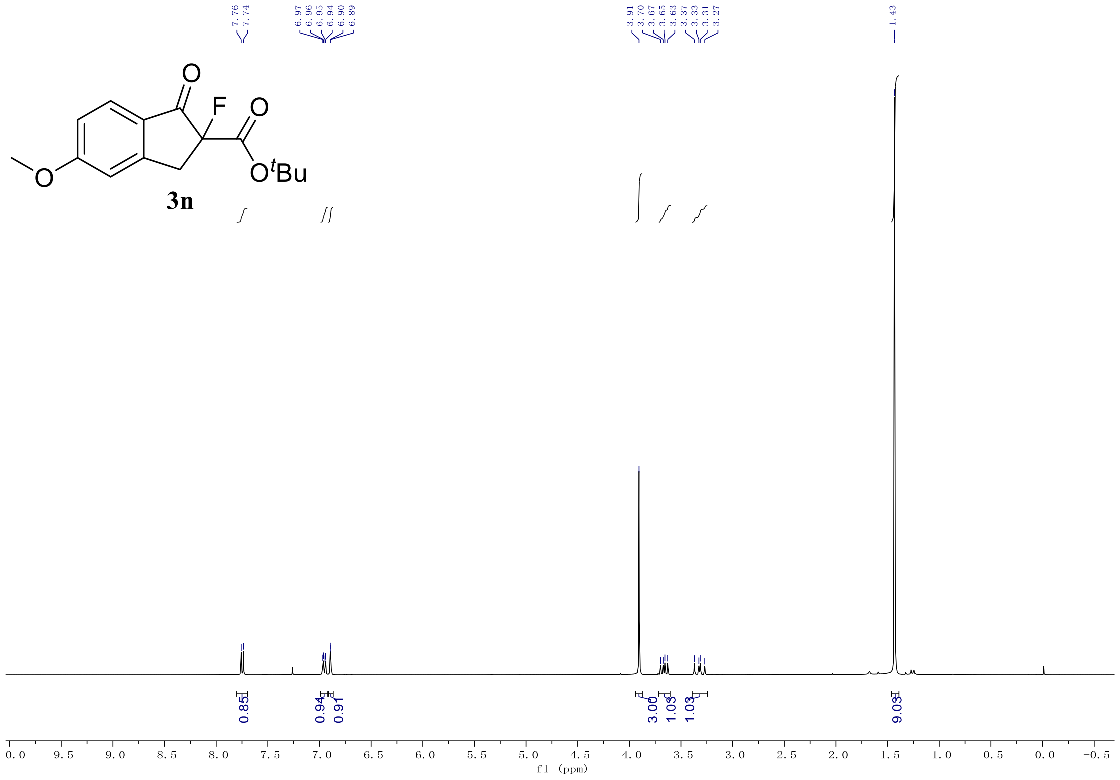

^{1}H NMR (CDCl$_3$) of **3n**

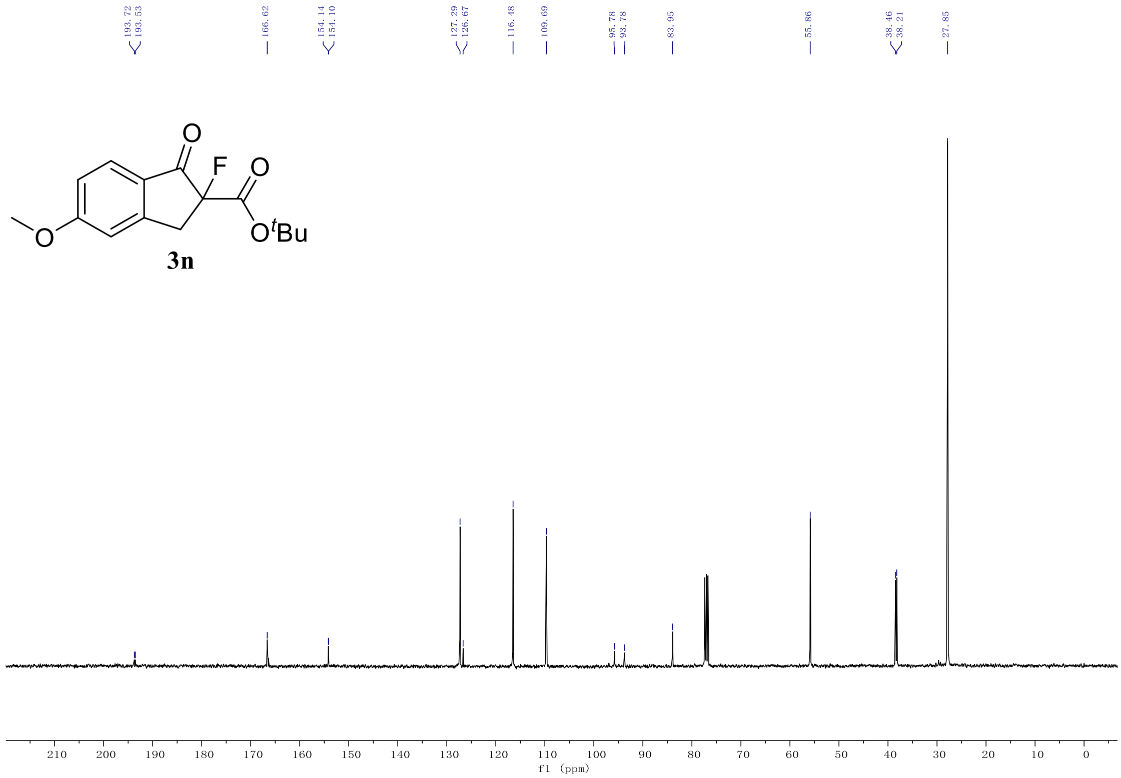

^{13}C NMR (CDCl$_3$) of **3n**

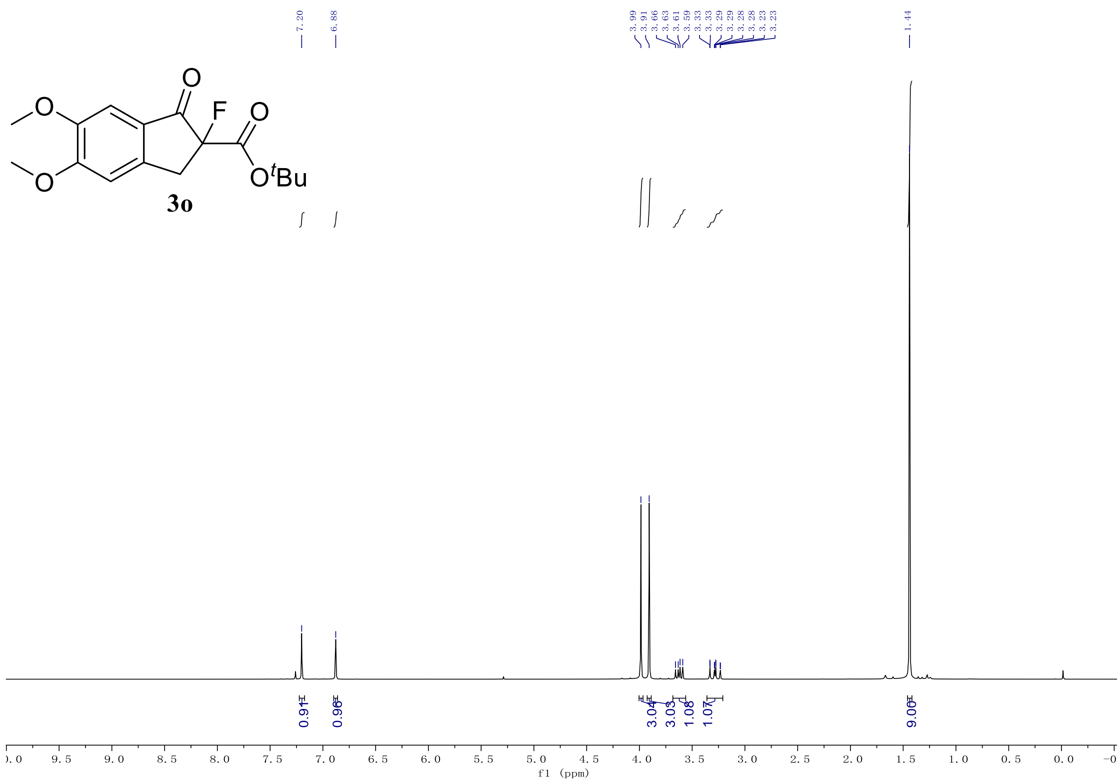

^{1}H NMR (CDCl$_3$) of **3o**

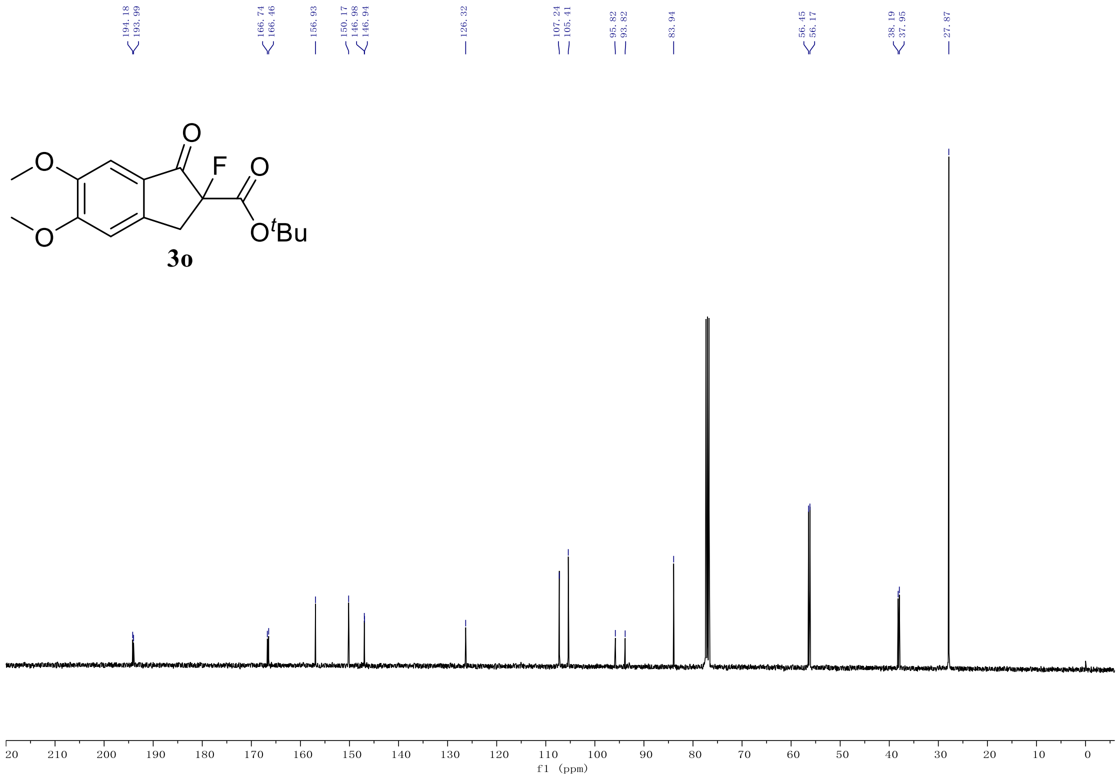

^{13}C NMR (CDCl$_3$) of **3o**

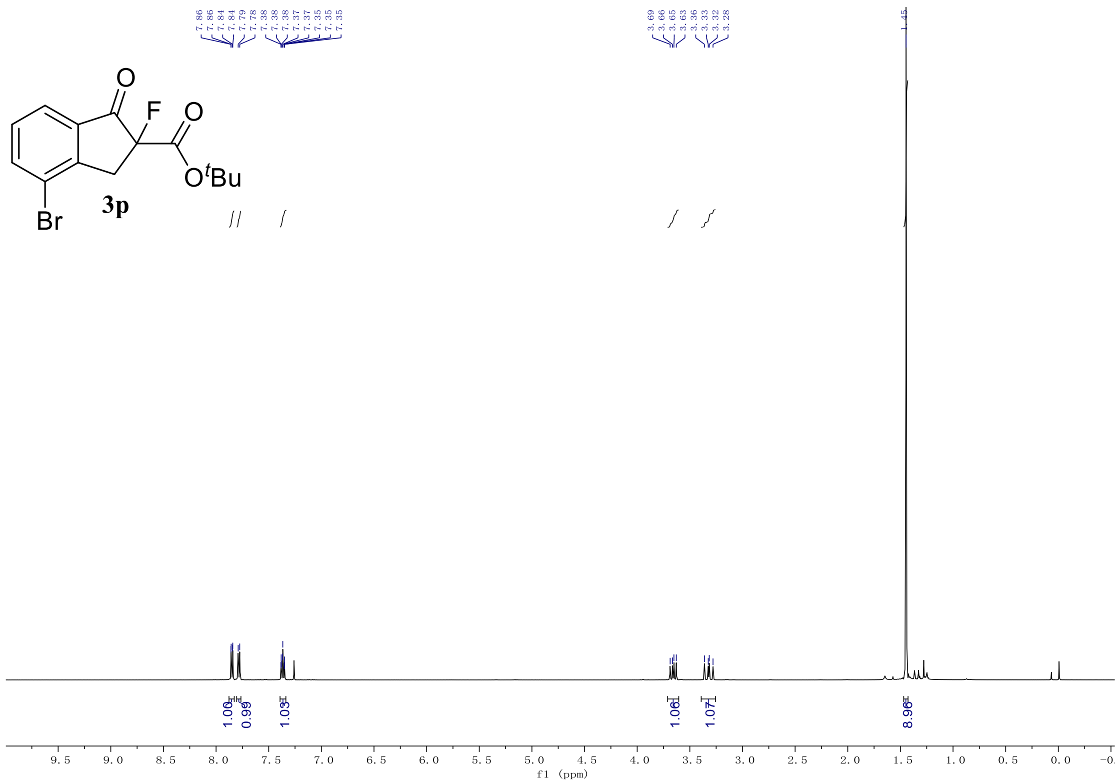

^{1}H NMR ($CDCl_3$) of **3p**

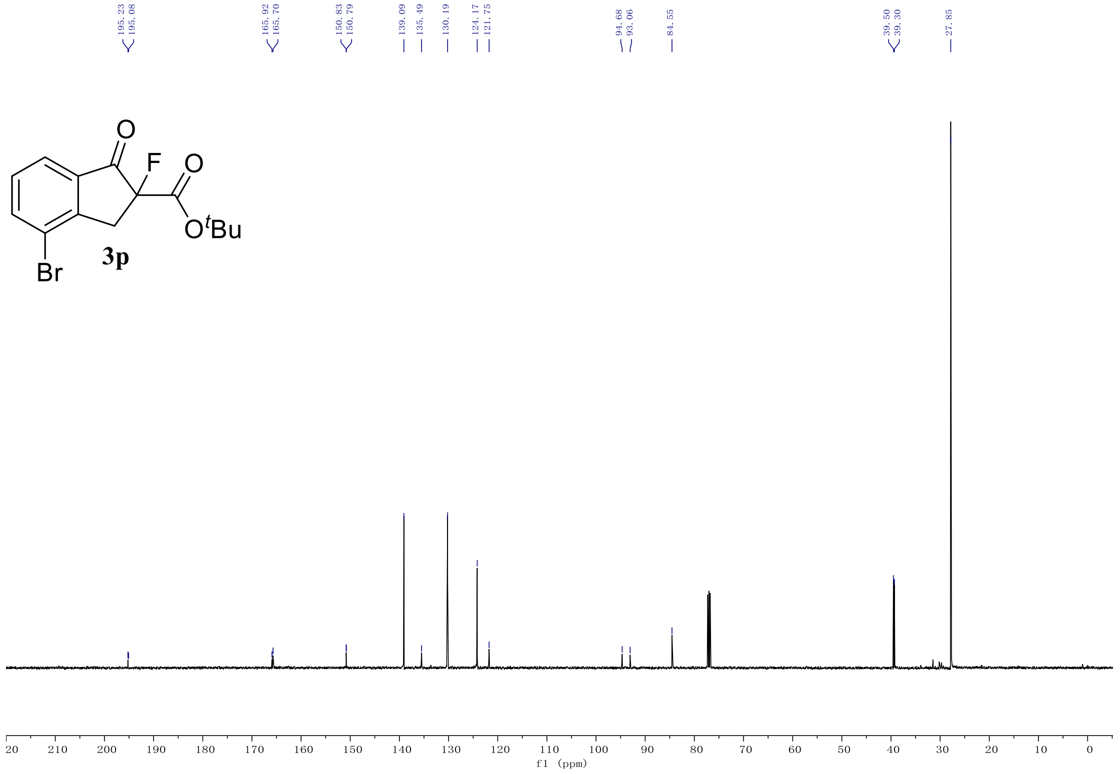

^{13}C NMR ($CDCl_3$) of **3p**

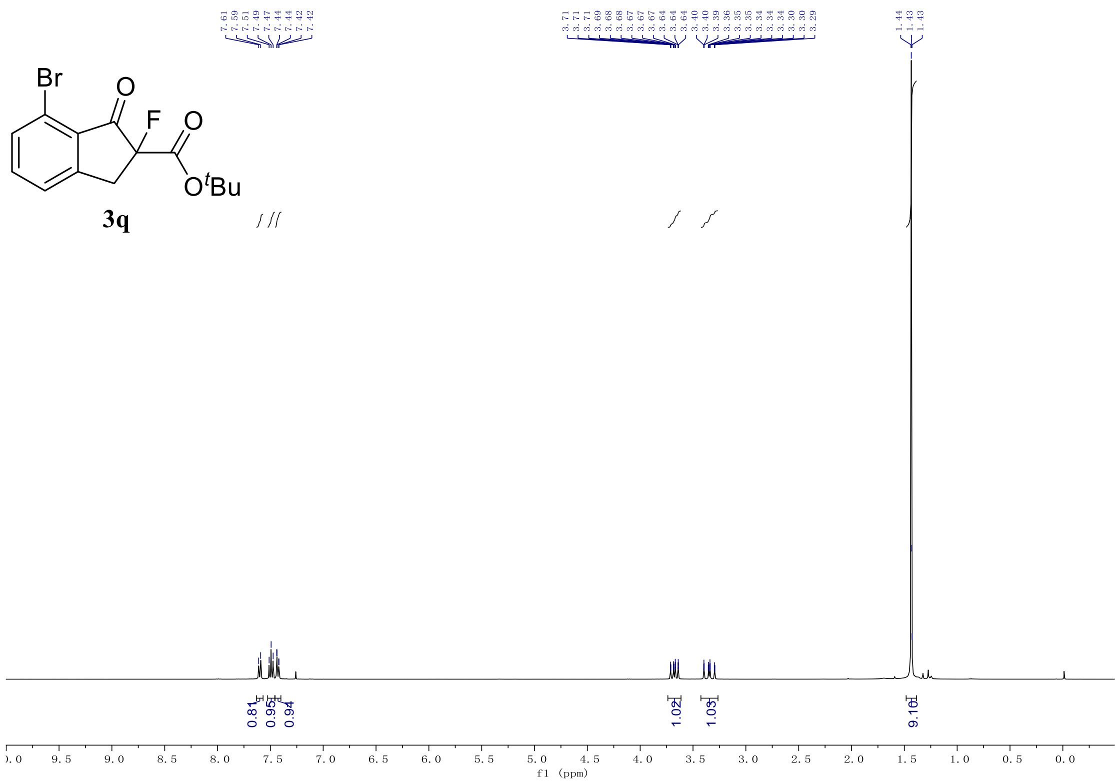

^{1}H NMR ($CDCl_3$) of **3q**

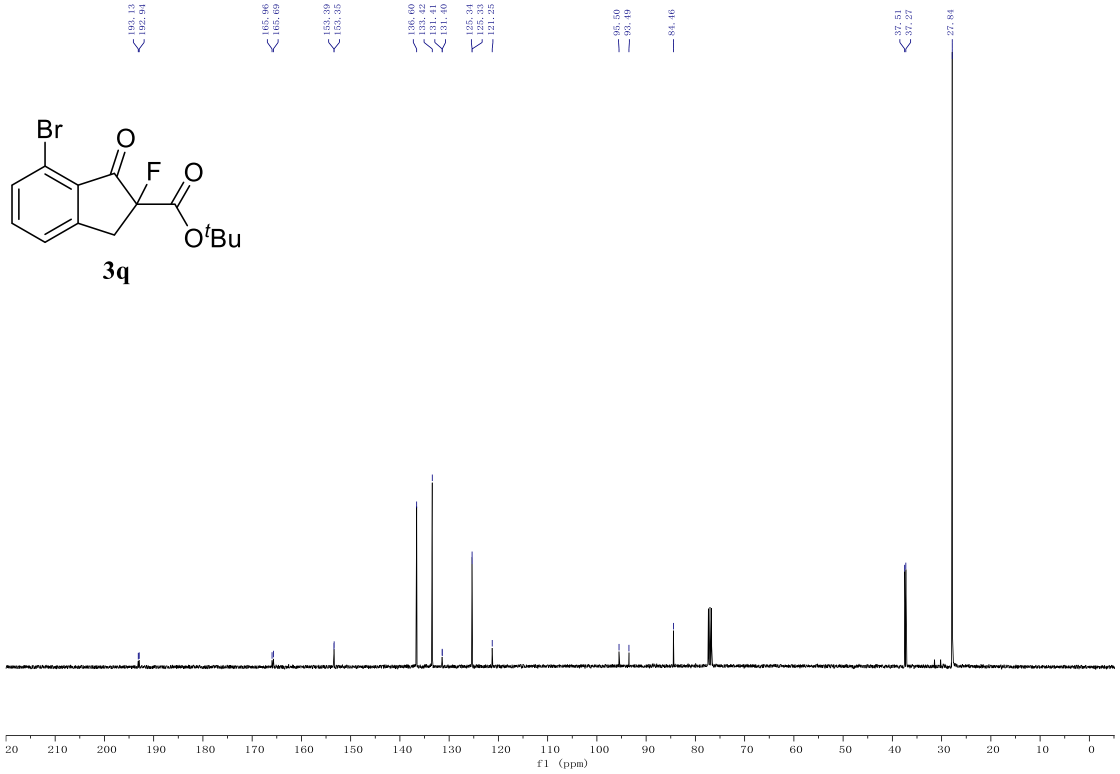

^{13}C NMR ($CDCl_3$) of **3q**

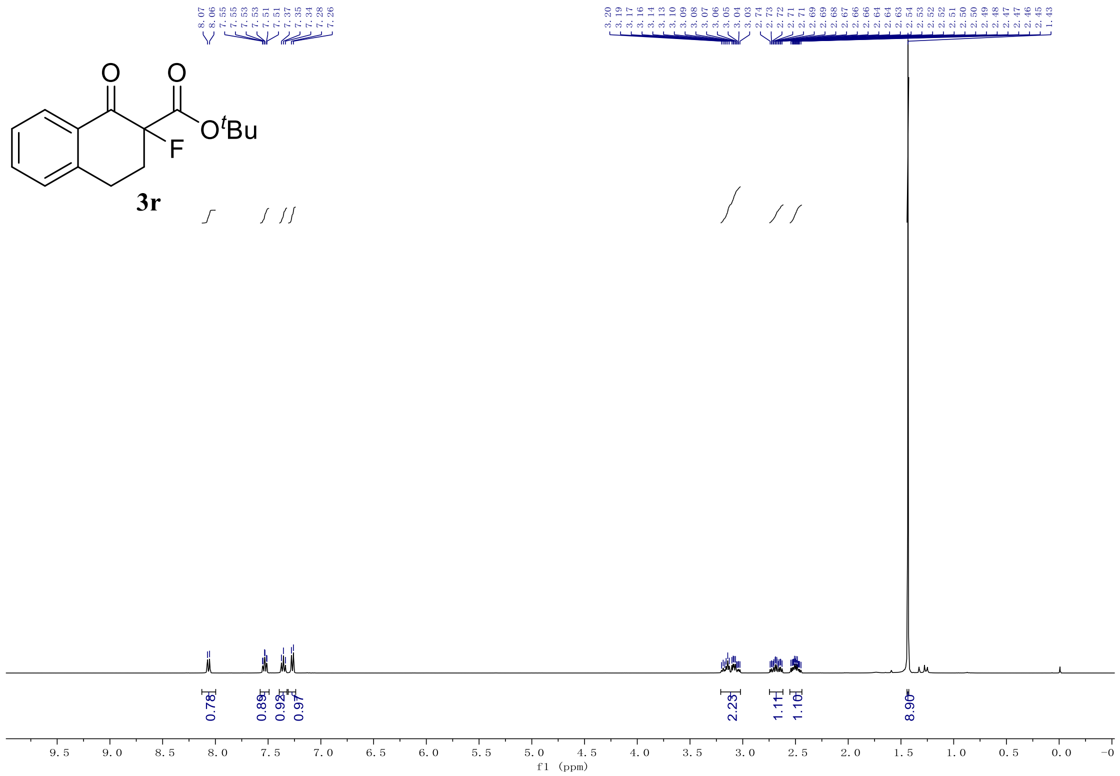

^{1}H NMR ($CDCl_3$) of **3r**

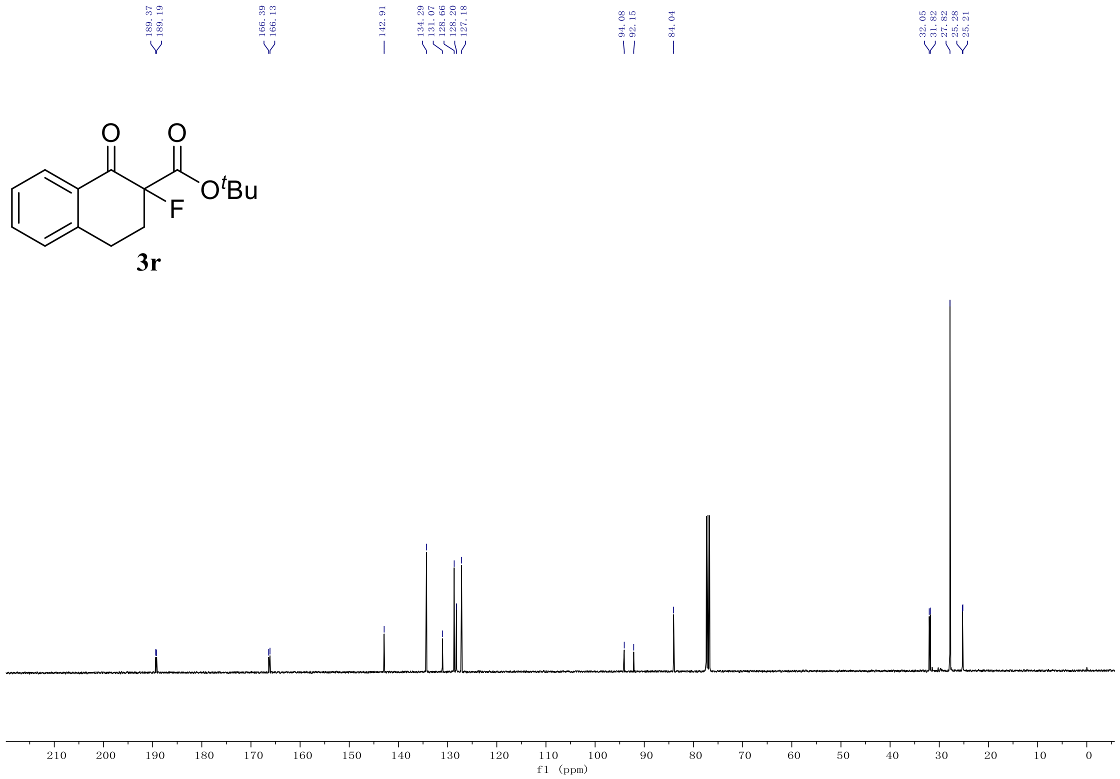

^{13}C NMR ($CDCl_3$) of **3r**

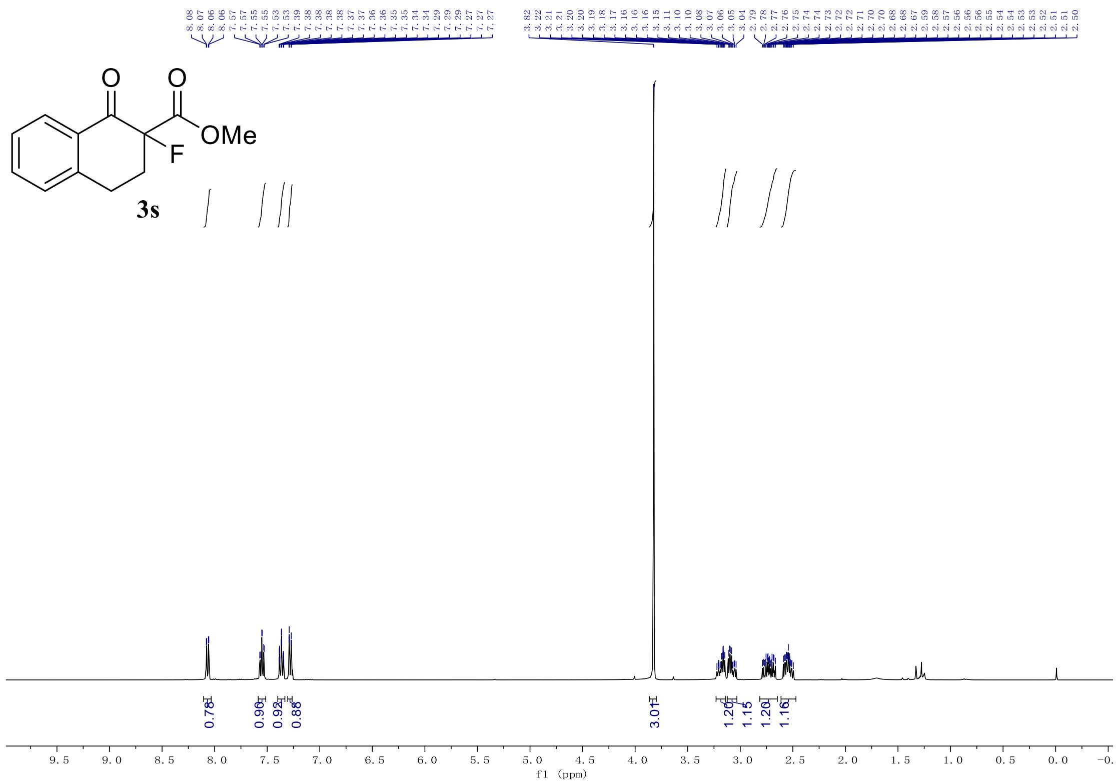

^{1}H NMR ($CDCl_3$) of **3s**

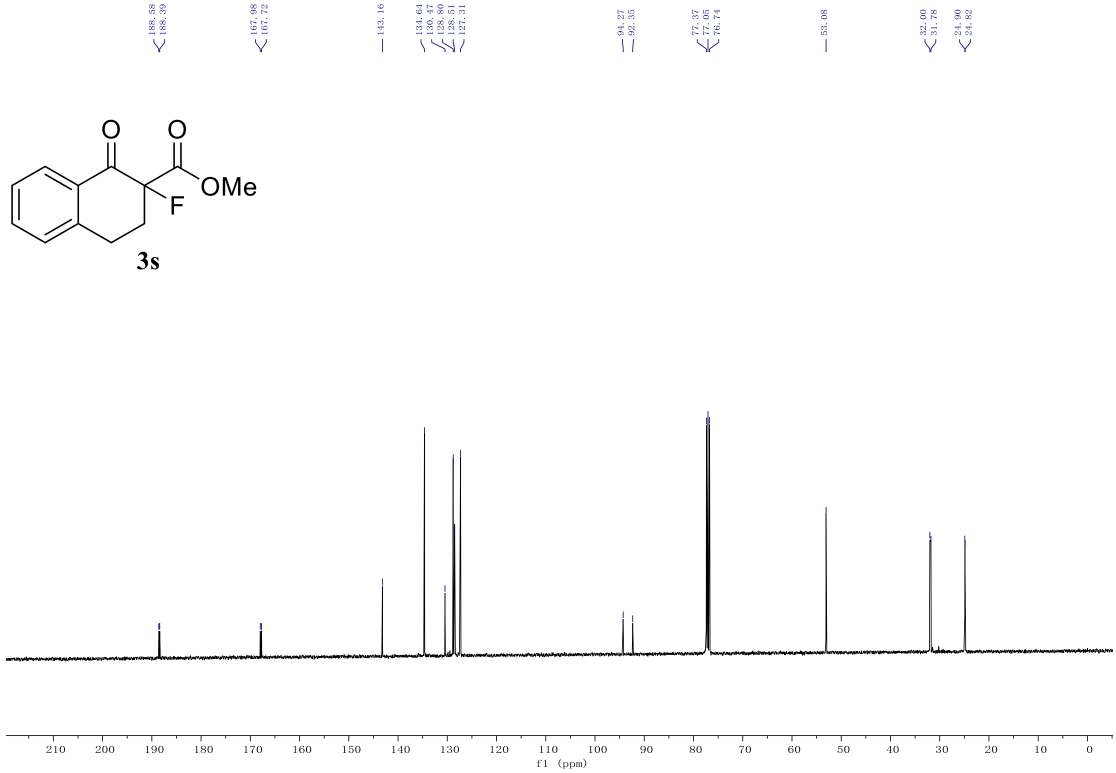

^{13}C NMR ($CDCl_3$) of **3s**

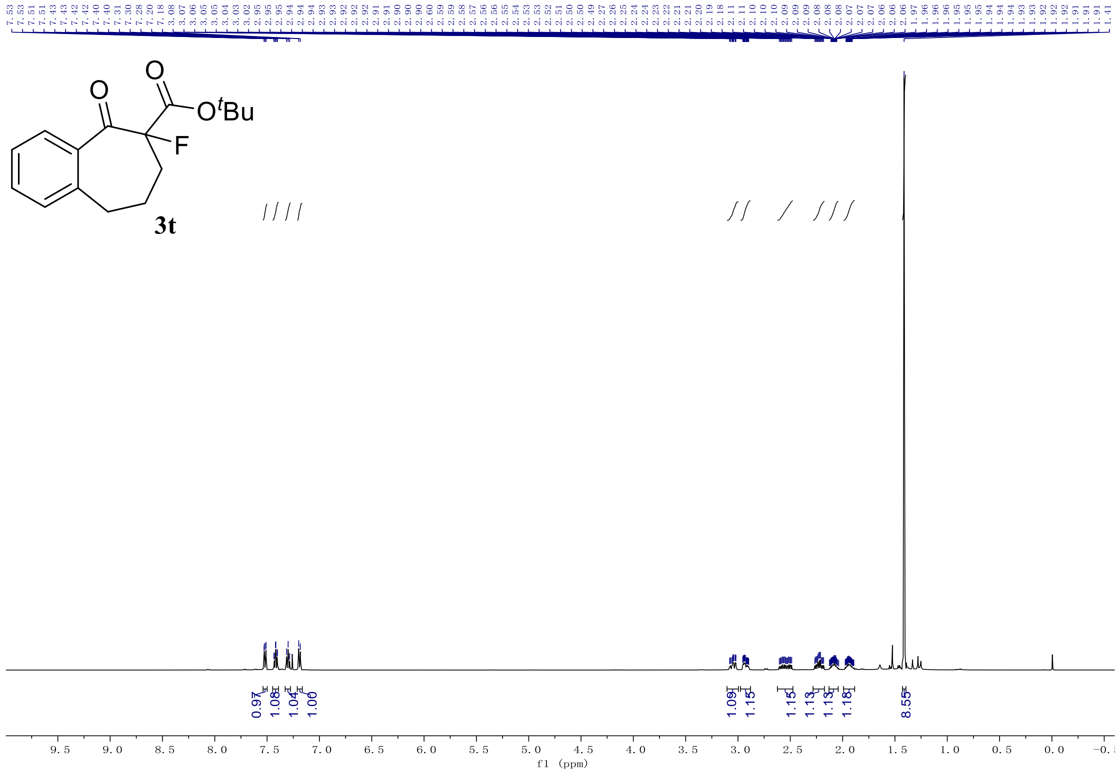

^{1}H NMR ($CDCl_3$) of **3t**

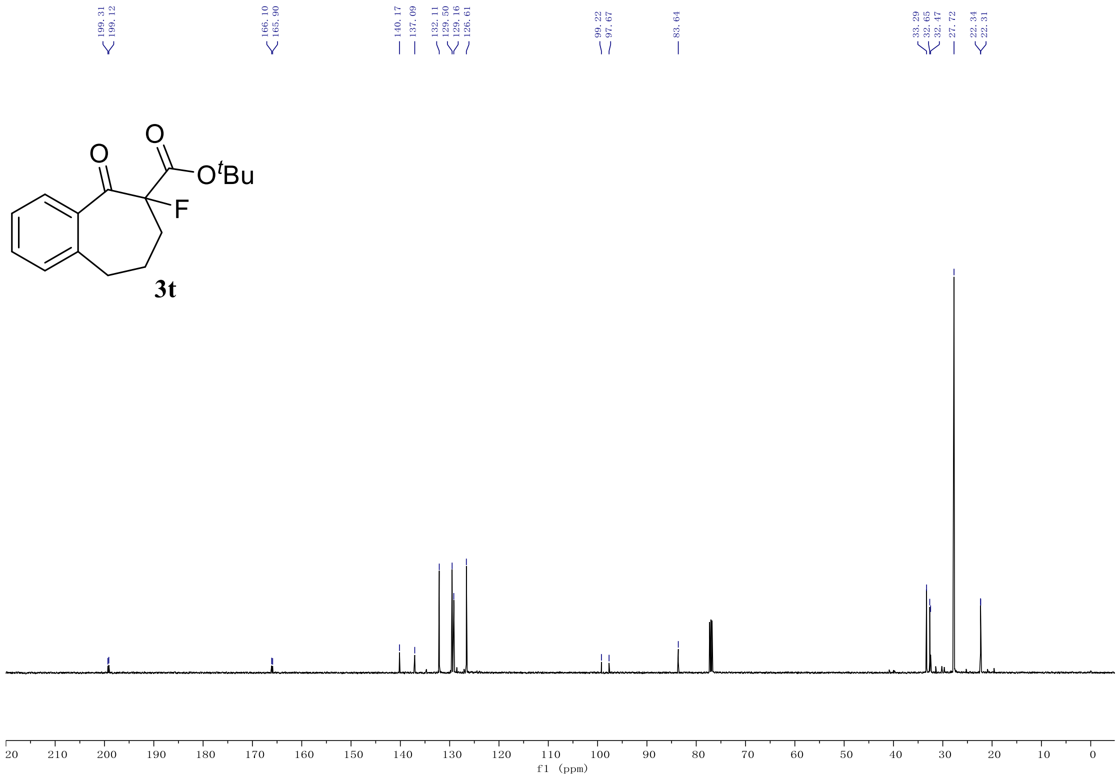

^{13}C NMR ($CDCl_3$) of **3t**

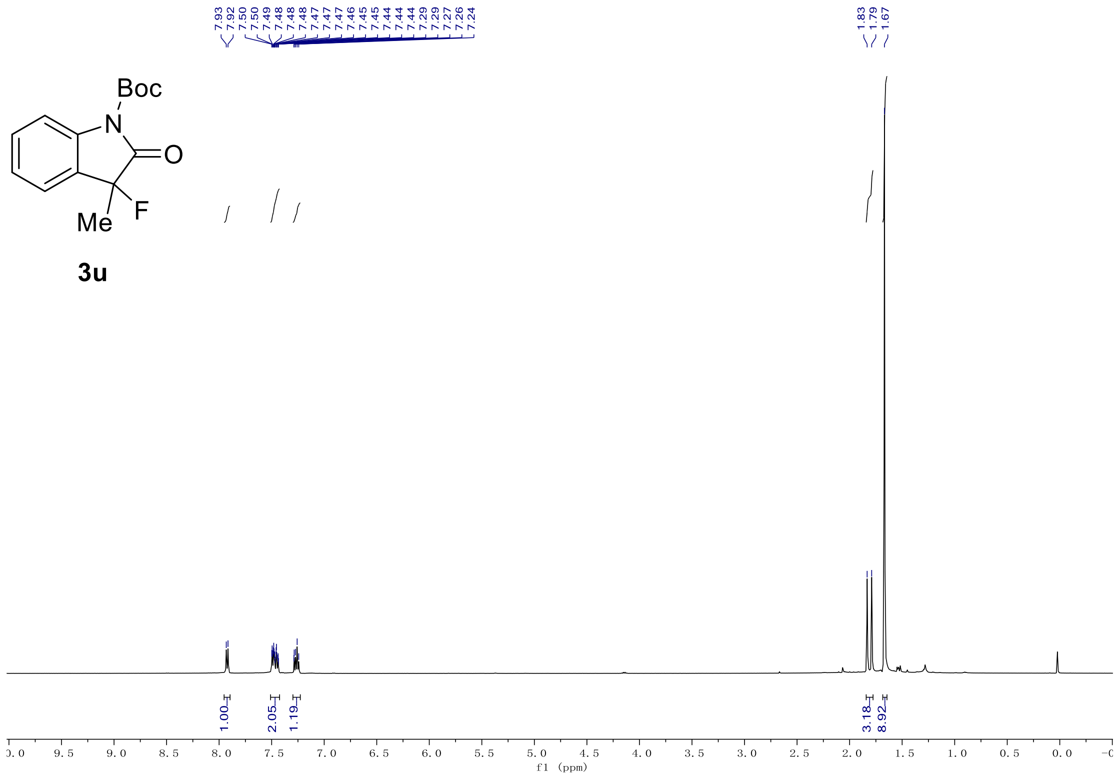

^{1}H NMR ($CDCl_3$) of **3u**

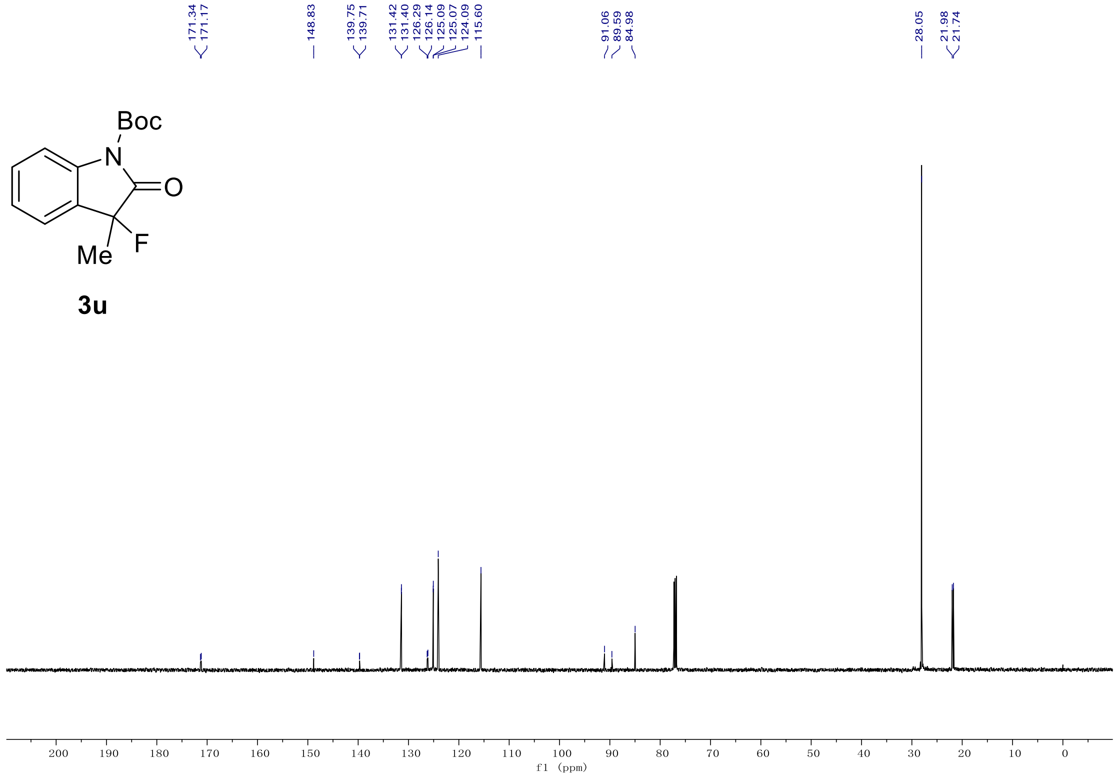

^{13}C NMR ($CDCl_3$) of **3u**

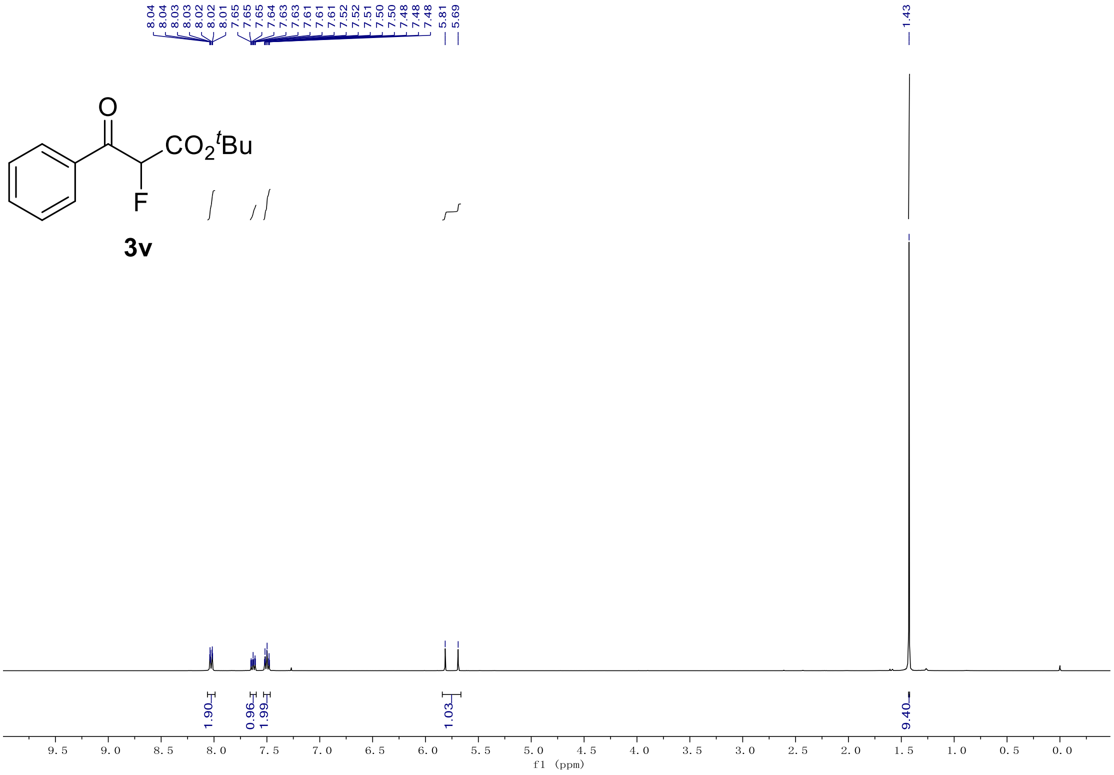

^{1}H NMR ($CDCl_3$) of **3v**

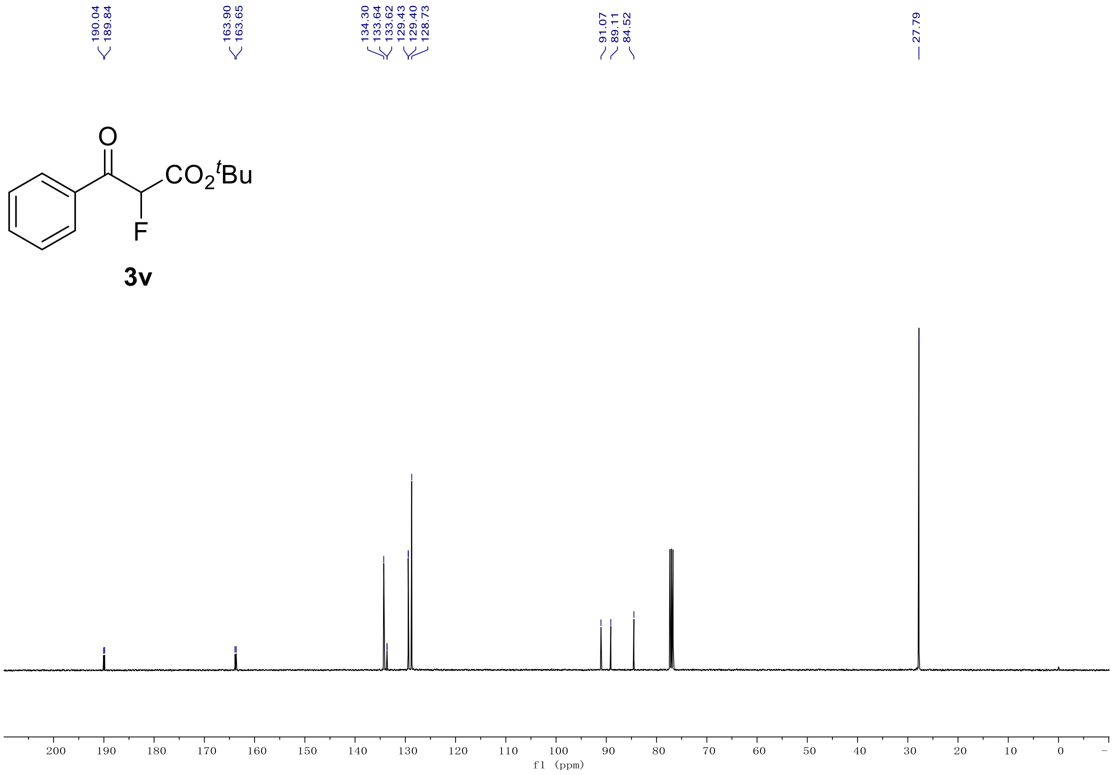

^{13}C NMR ($CDCl_3$) of **3v**

8. HPLC Spectra of the products

Standard Curves for yield determination and HPLC traces for ee determination

Standard Curve for **3a** is displayed below:

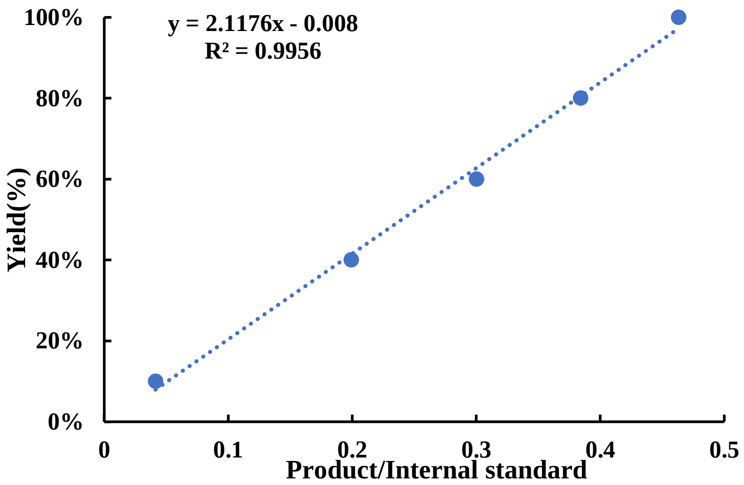

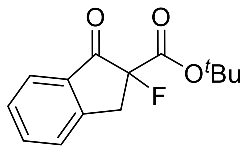

3a

Enantiomeric excess was established by HPLC: AD-H, n-hexane/isopropanol = 98:2, flow rate 0.5 mL/min, t major = 16.094 min, t minor = 18.109 min.

HPLC trace of racemate **3a**:

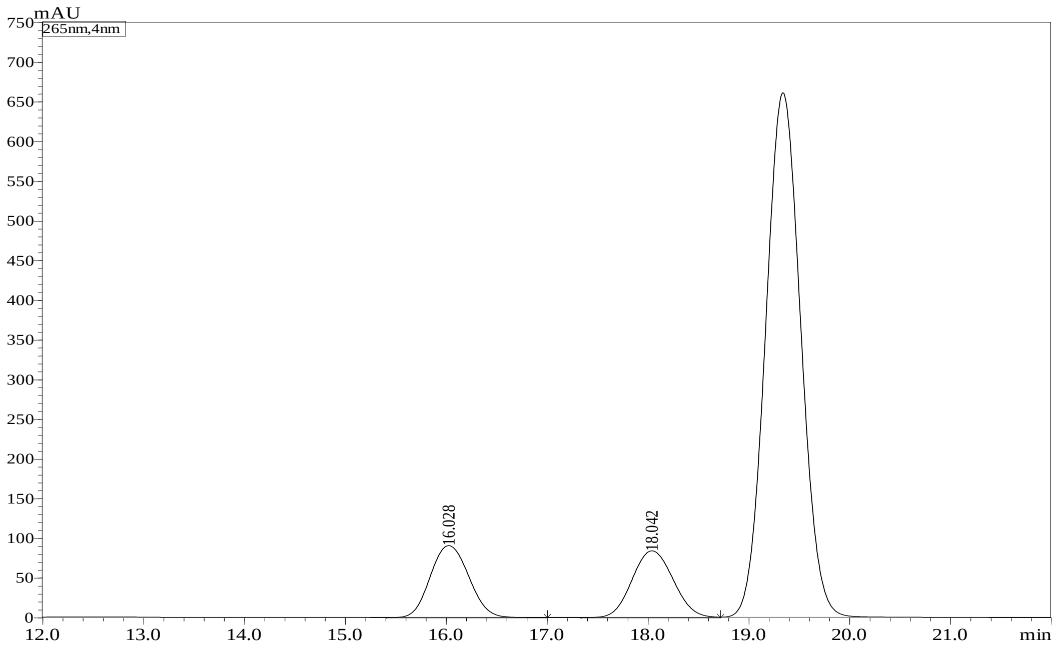

Peak No.	Retention time	Area	Height	Area%
1	16.028	2398888	90541	50.187
2	18.042	2380999	83974	49.813
Total		4779887	174515	100.000

HPLC trace of enantiomerically enriched **3a**:

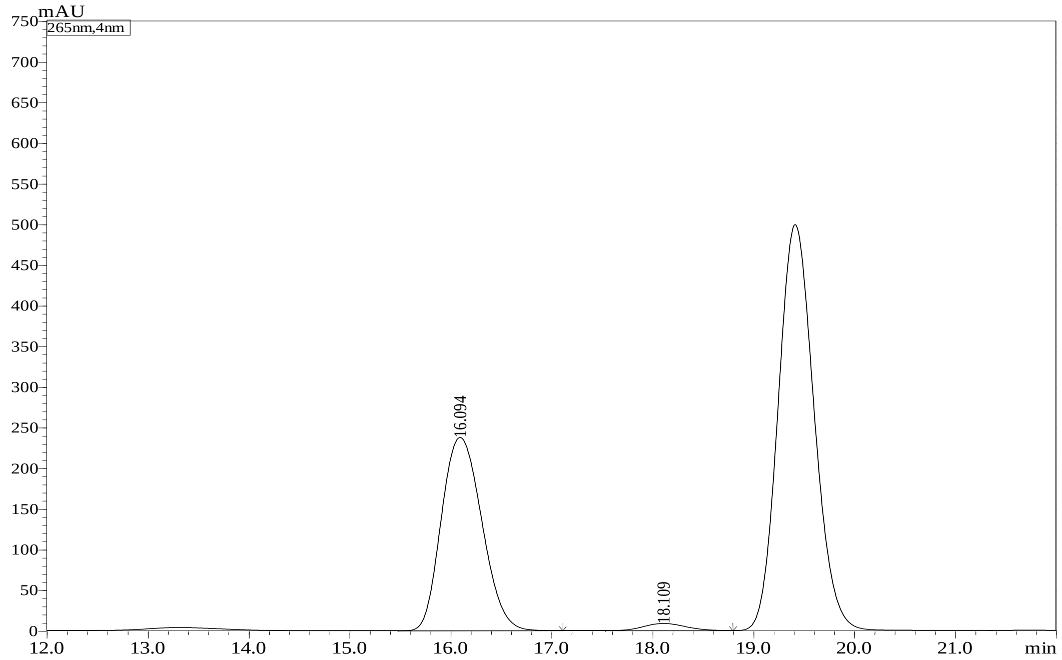

Peak No.	Retention time	Area	Height	Area%
1	16.094	6629842	237662	96.404
2	18.109	247277	8663	3.596
Total		6877120	246326	100.000

Standard Curve for **3b** is displayed below:

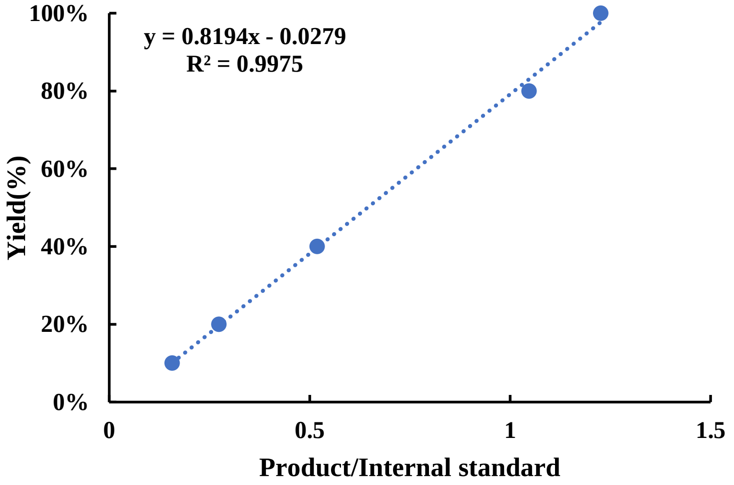

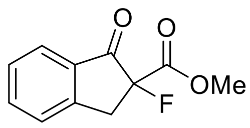

3b

Enantiomeric excess was established by HPLC: AD-H, n-hexane/isopropanol = 98:2, flow rate 0.4 mL/min, t major = 34.551 min, t minor = 36.243 min.

HPLC trace of racemate **3b**:

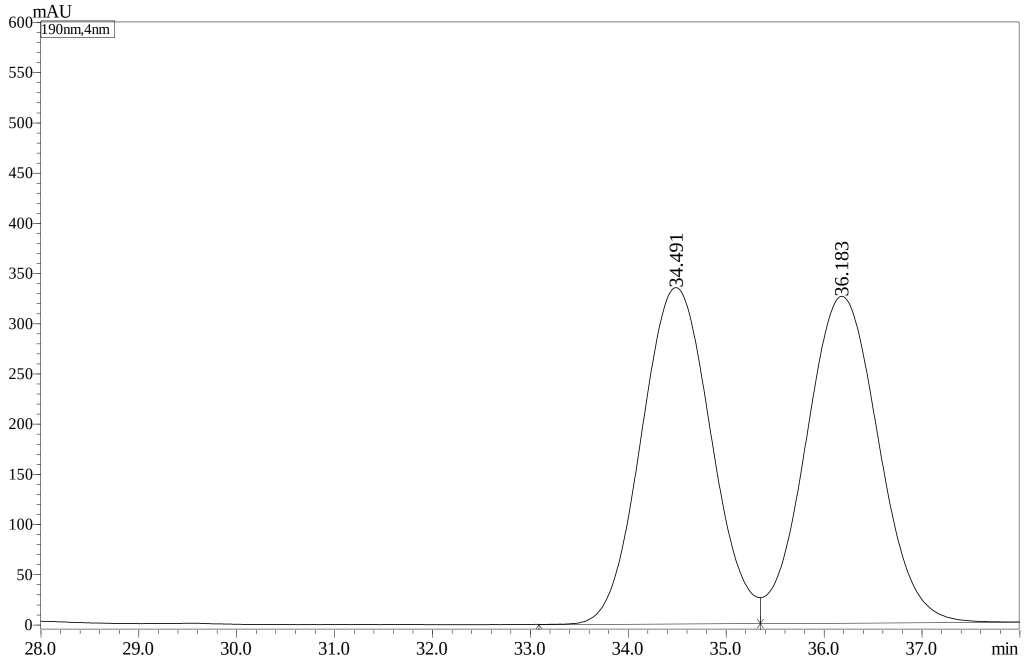

Peak No.	Retention time	Area	Height	Area%
1	34.491	16488473	334719	49.600
2	36.183	16754743	325371	50.400
Total		33243216	660091	100.000

HPLC trace of enantiomerically enriched **3b**:

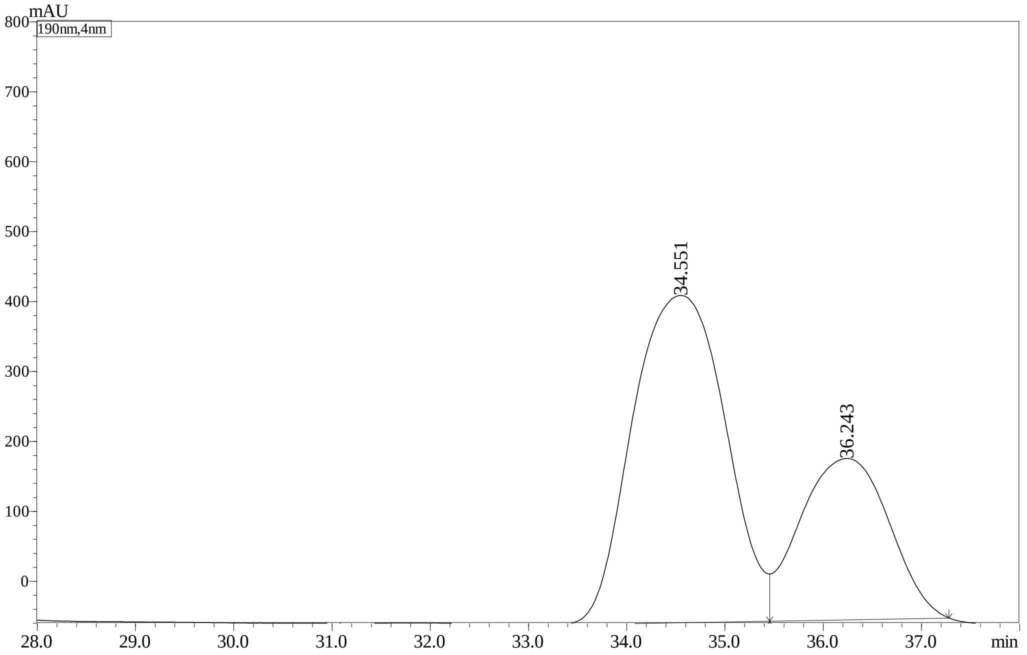

Peak No.	Retention time	Area	Height	Area%
1	34.551	30716867	467630	67.893
2	36.243	14526448	230427	32.107
Total		45243314	698057	100.000

Standard Curve for **3c** is displayed below:

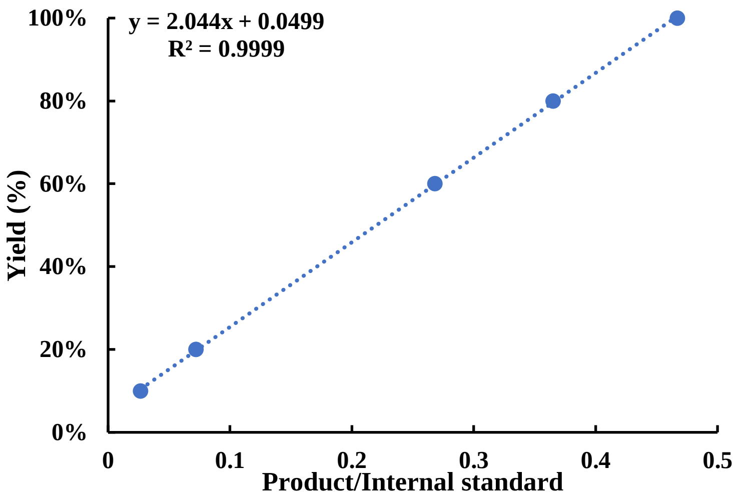

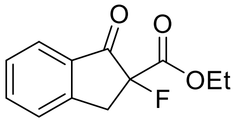

3c

Enantiomeric excess was established by HPLC: AD-H, n-hexane/isopropanol = 98:2, flow rate 0.5mL/min, t major = 22.740 min, t minor = 24.029 min.

HPLC trace of racemate **3c**:

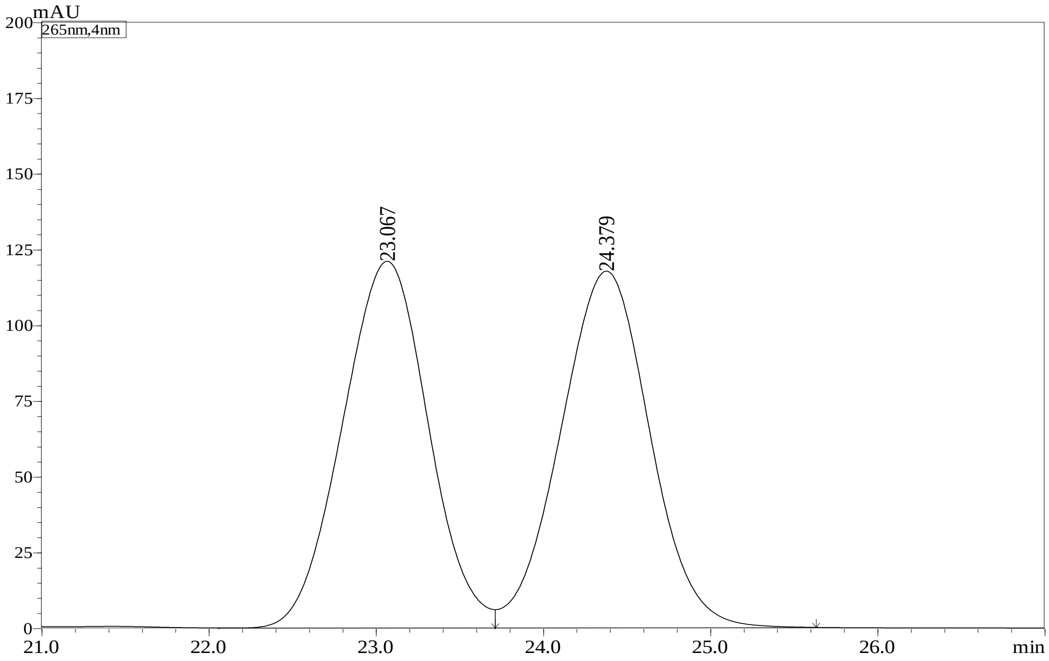

Peak No.	Retention time	Area	Height	Area%
1	23.067	4301403	120924	49.777
2	24.379	4339923	117519	50.223
Total		8641326	238443	100.000

HPLC trace of enantiomerically enriched **3c**:

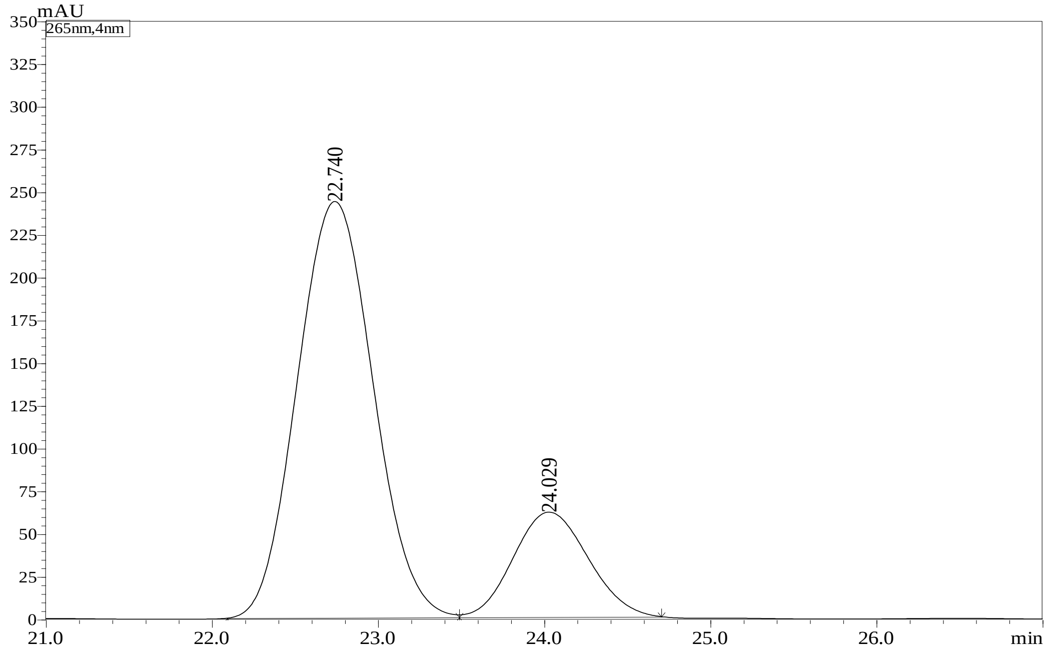

Peak No.	Retention time	Area	Height	Area%
1	22.740	7641711	242520	80.578
2	24.029	1841935	60175	19.422
Total		9664034	305074	100.000

Standard Curve for **3d** is displayed below:

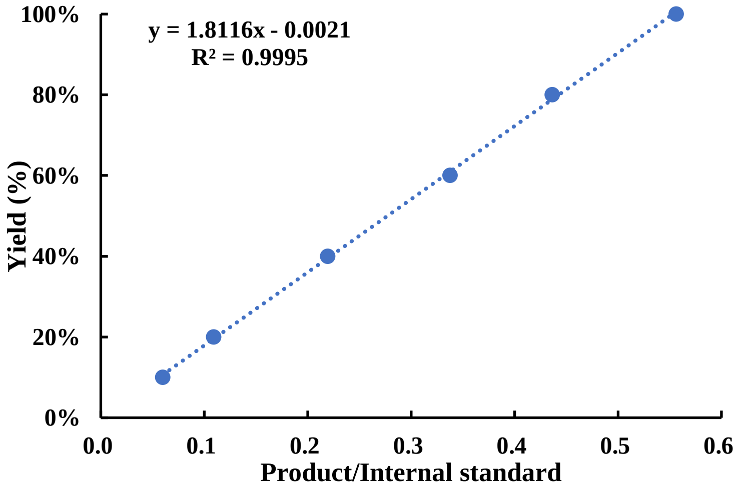

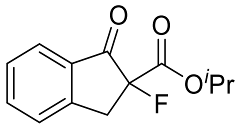

3d

Enantiomeric excess was established by HPLC: IK-3, n-hexane/isopropanol = 93:7, flow rate 1 mL/min, t major = 12.143 min, t minor = 13.022 min.

HPLC trace of racemate **3d**:

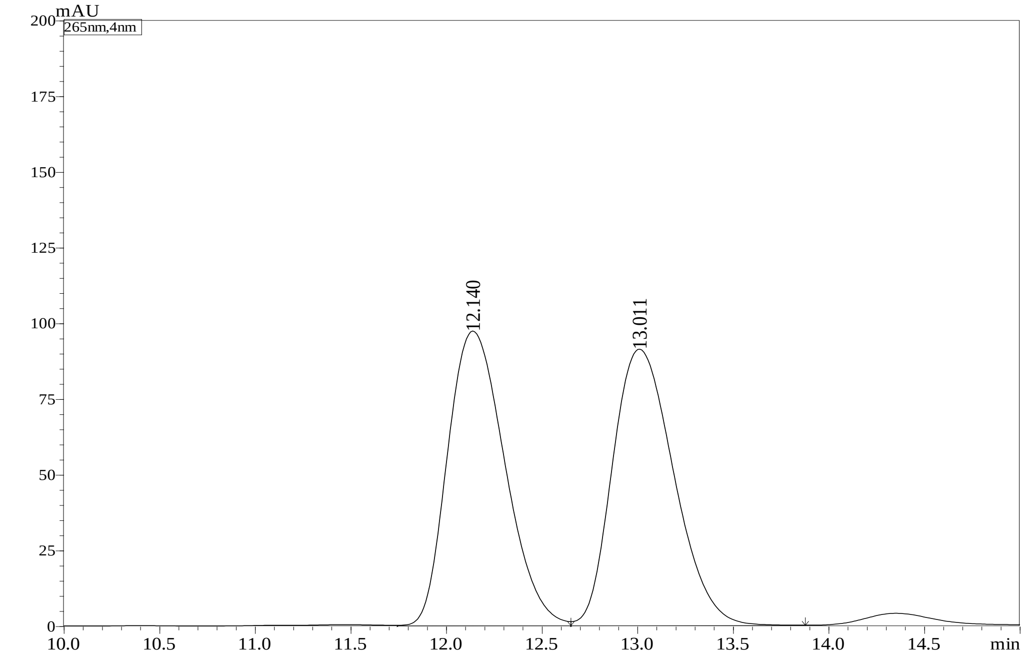

Peak No.	Retention time	Area	Height	Area%
1	12.140	2022631	97251	49.899
2	13.011	2030788	91215	50.101
Total		4053419	188467	100.000

HPLC trace of enantiomerically enriched **3d**:

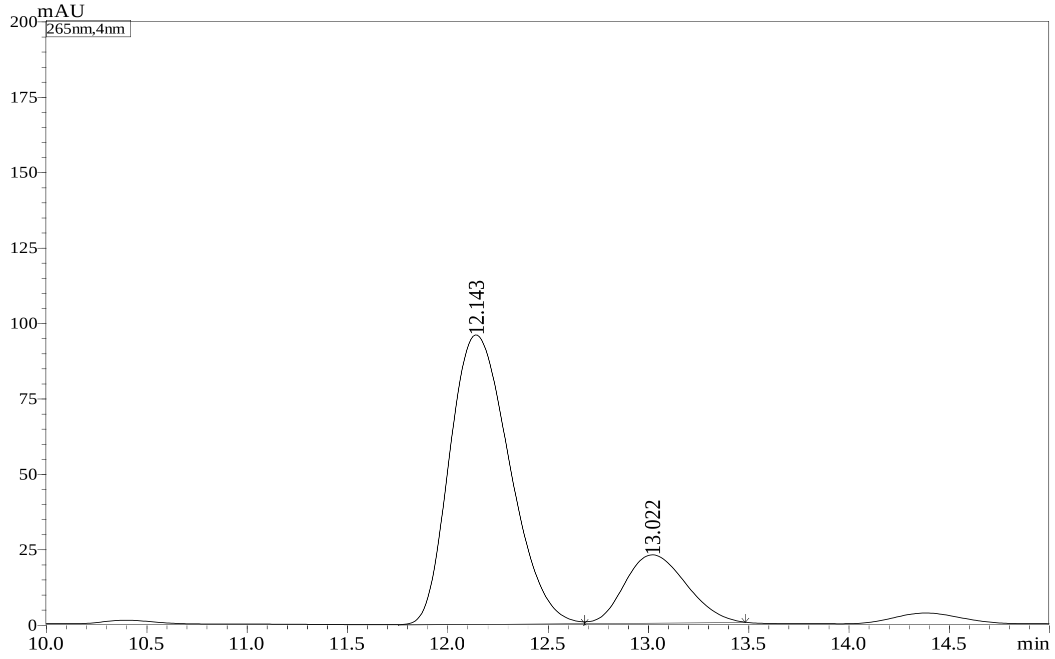

Peak No.	Retention time	Area	Height	Area%
1	12.143	1986909	95823	80.572
2	13.022	479081	22516	19.428
Total		2465990	118339	100.000

Standard Curve for **3e** is displayed below:

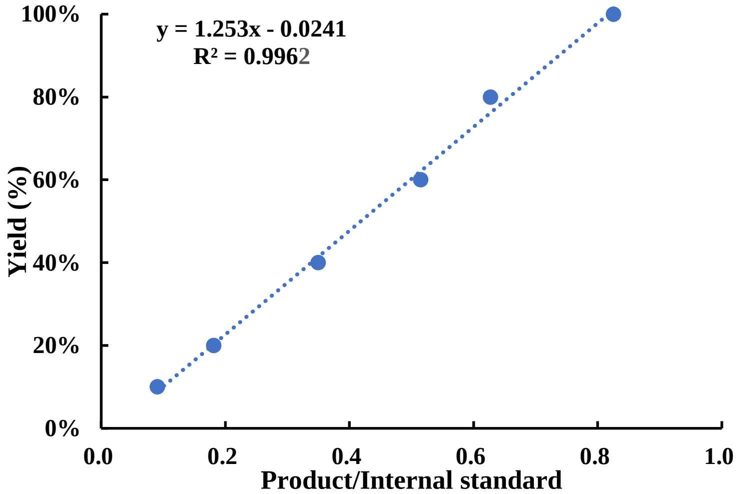

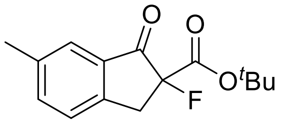

3e

Enantiomeric excess was established by HPLC: IE-3, n-hexane/isopropanol = 90:10, flow rate 1 mL/min, t major = 12.299 min, t minor = 14.076 min.

HPLC trace of racemate **3e**:

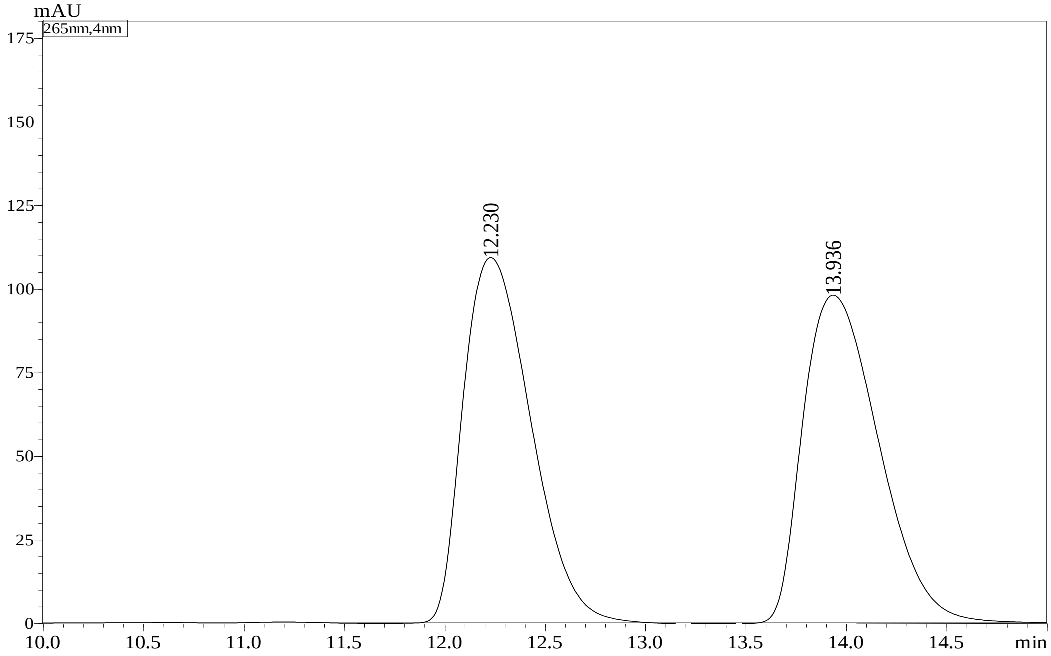

Peak No.	Retention time	Area	Height	Area%
1	12.230	2577041	109445	49.864
2	13.936	2591086	98162	50.136
Total		5168127	207608	100.000

HPLC trace of enantiomerically enriched **3e**:

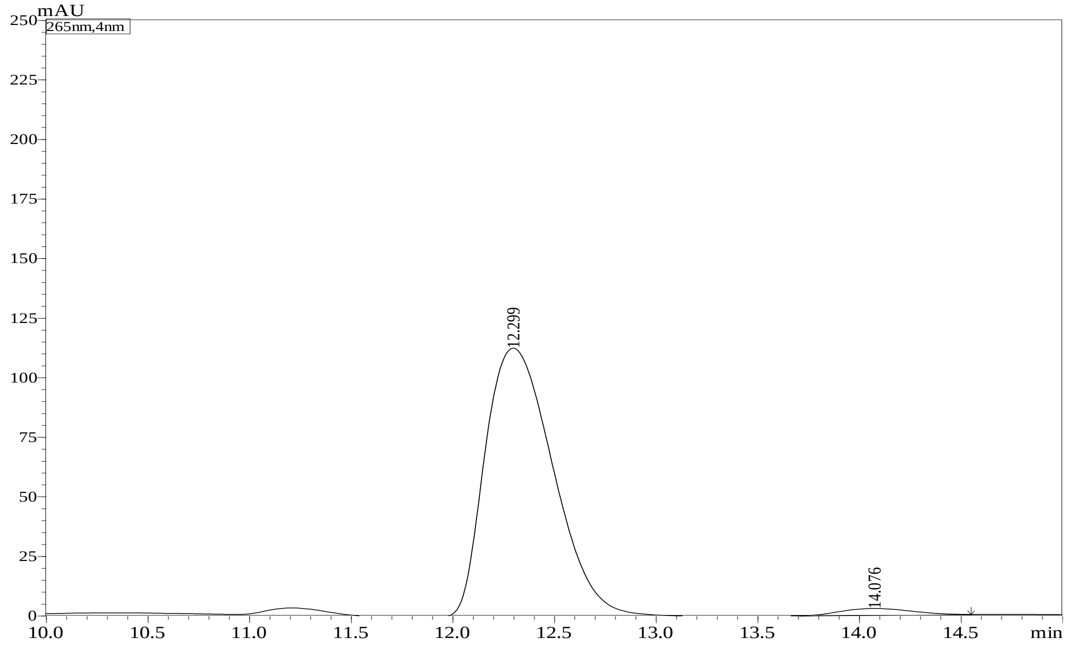

Peak No.	Retention time	Area	Height	Area%
1	12.299	2628541	112831	97.543
2	14.076	66201	2784	2.457
Total		2694742	115615	100.000

Standard Curve for **3f** is displayed below:

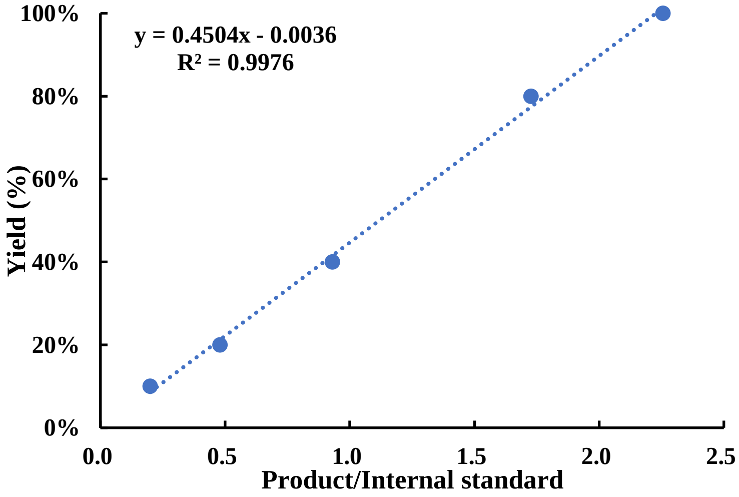

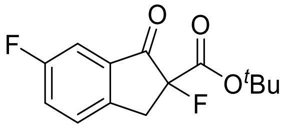

3f

Enantiomeric excess was established by HPLC: IK-3, n-hexane/isopropanol = 90:10, flow rate1mL/min, t major = 7.197 min, t minor = 8.208 min.

HPLC trace of racemate **3f**:

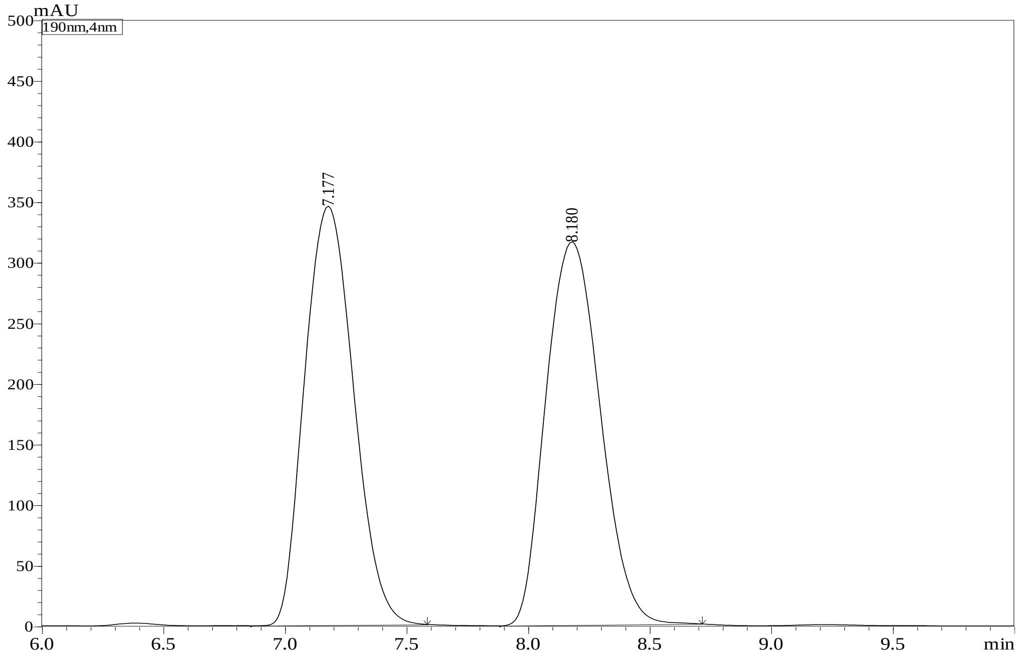

Peak No.	Retention time	Area	Height	Area%
1	7.177	4850222	345617	49.640
2	8.180	4920597	316236	50.360
Total		9770819	661854	100.000

HPLC trace of enantiomerically enriched **3f**:

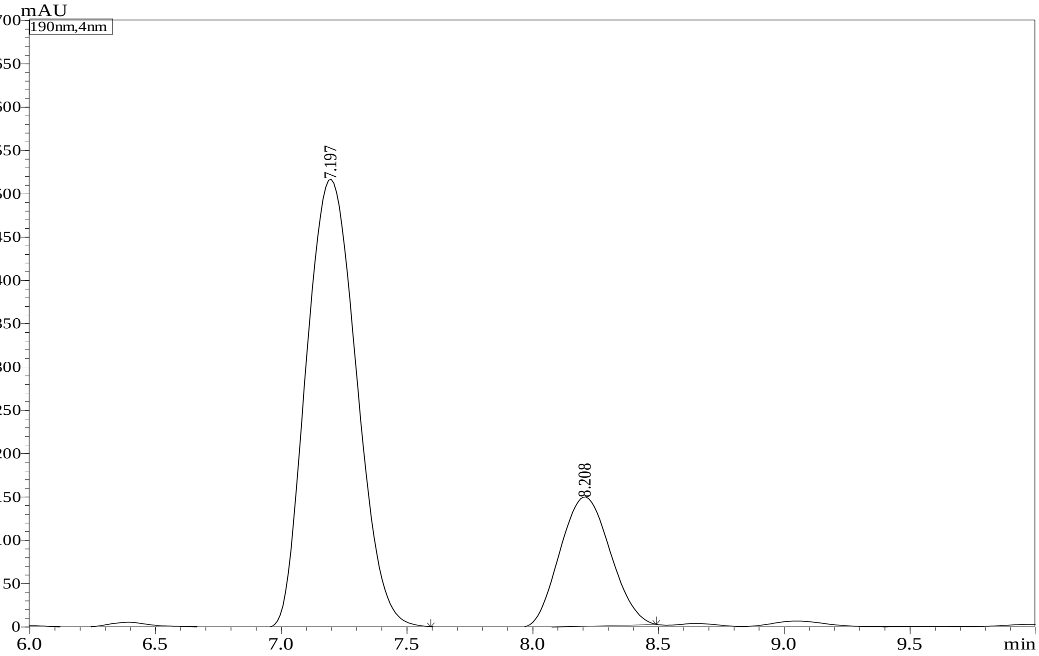

Peak No.	Retention time	Area	Height	Area%
1	7.197	7177890	517478	77.555
2	8.208	2077378	148818	22.445
Total		9255268	666297	100.000

Standard Curve for **3g** is displayed below:

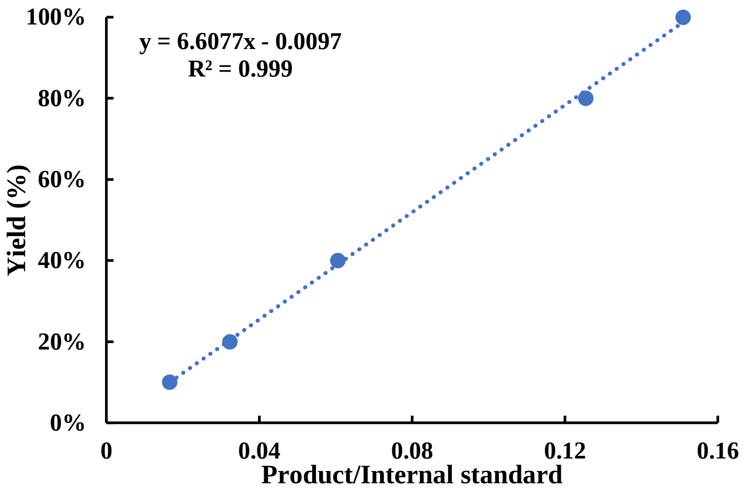

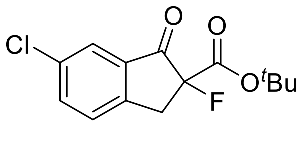

3g

Enantiomeric excess was established by HPLC: IK-3, n-hexane/isopropanol = 90:10, flow rate1mL/min, t major = 7.446 min, t minor = 8.566 min.

HPLC trace of racemate **3g**:

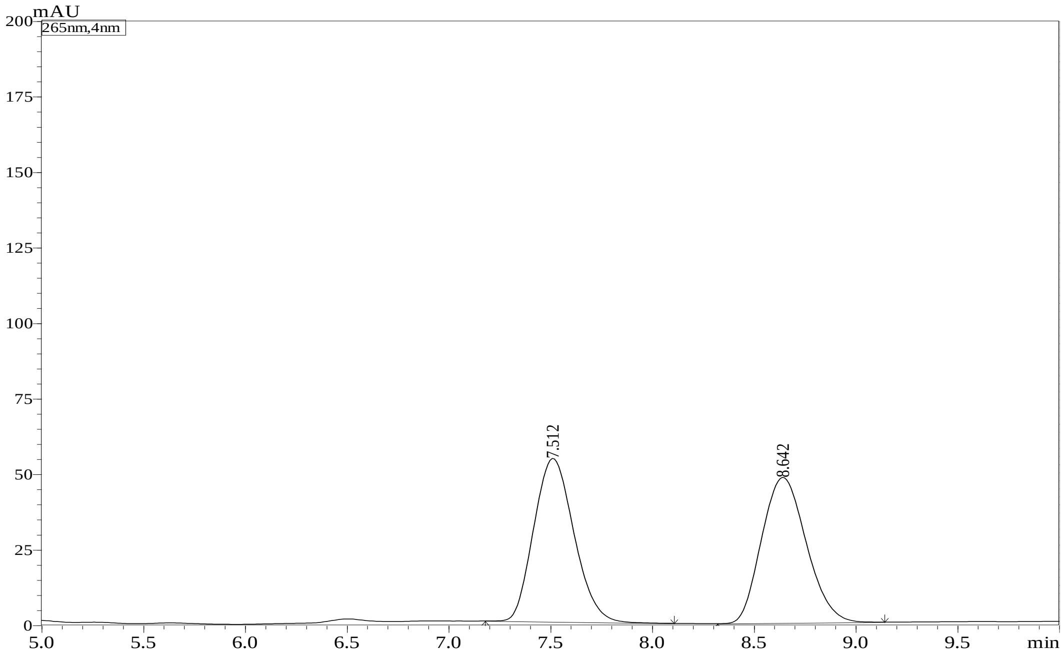

Peak No.	Retention time	Area	Height	Area%
1	7.512	742542	54096	50.245
2	8.642	735302	48198	49.755
Total		1477843	102294	100.000

HPLC trace of enantiomerically enriched **3g**:

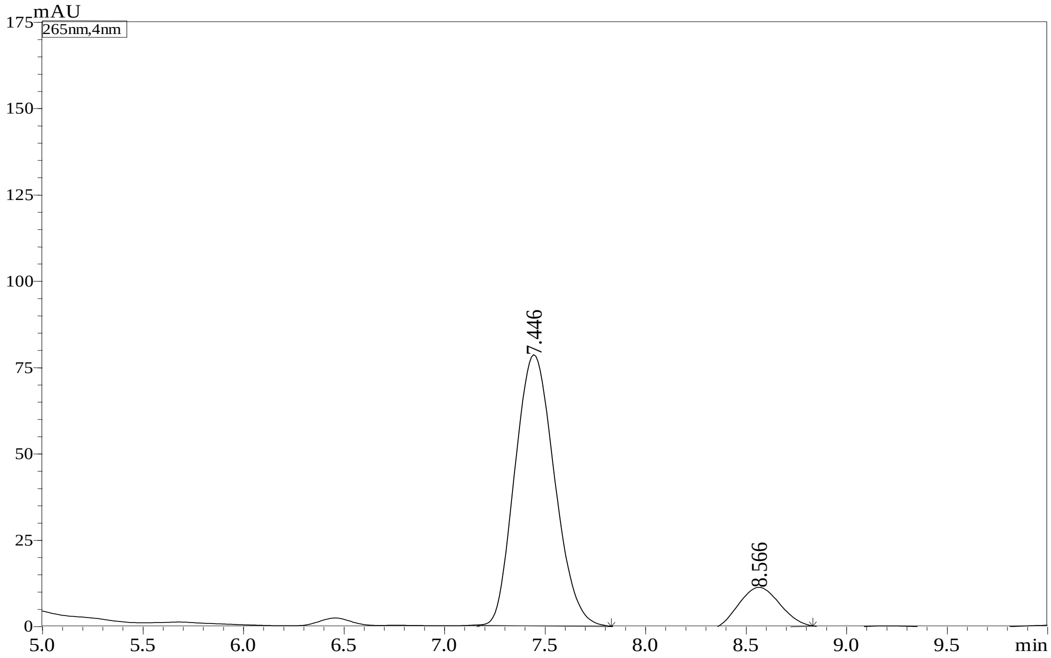

Peak No.	Retention time	Area	Height	Area%
1	7.446	1070811	78423	86.627
2	8.566	165310	11577	13.373
Total		1236121	90000	100.000

Standard Curve for **3h** is displayed below:

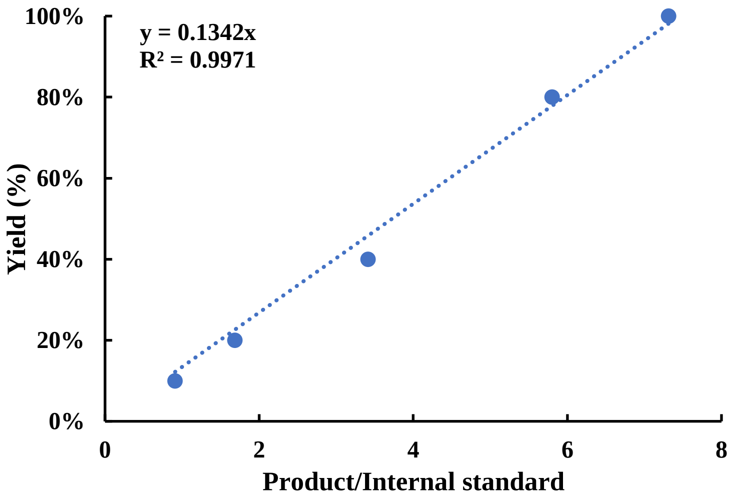

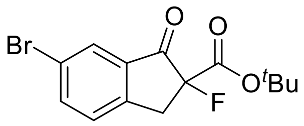

3h

Enantiomeric excess was established by HPLC: OD-H, n-hexane/isopropanol = 95:5, flow rate 0.75 mL/min, t major = 9.221 min, t minor = 9.854 min.

HPLC trace of racemate **3h**:

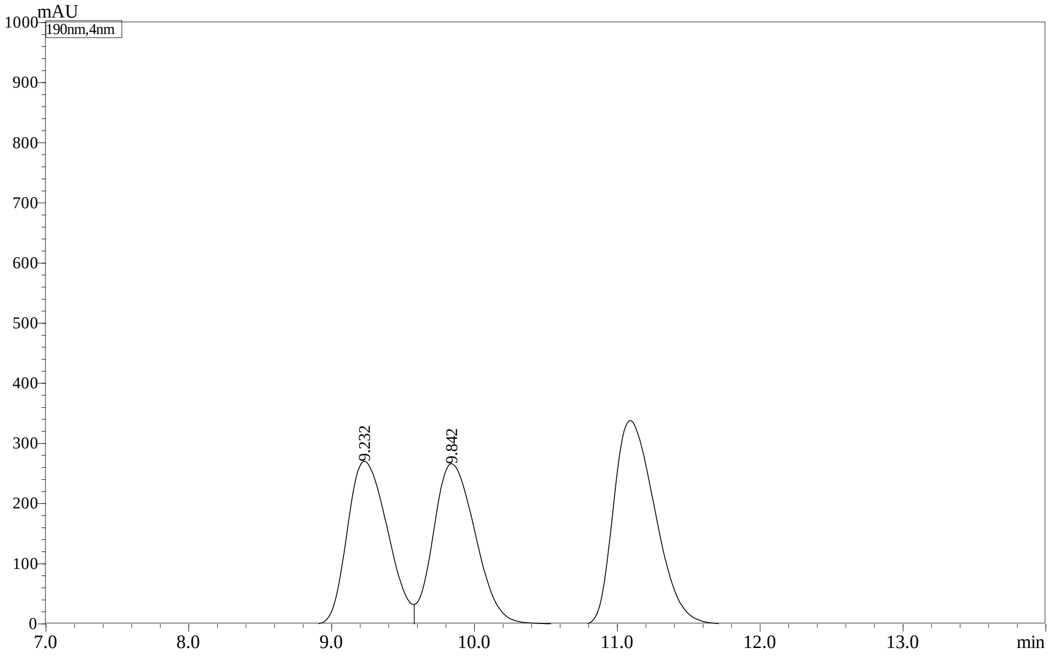

Peak No.	Retention time	Area	Height	Area%
1	9.232	5364001	271890	49.228
2	9.842	5532156	267956	50.772
Total		10896157	539846	100.000

HPLC trace of enantiomerically enriched **3h**:

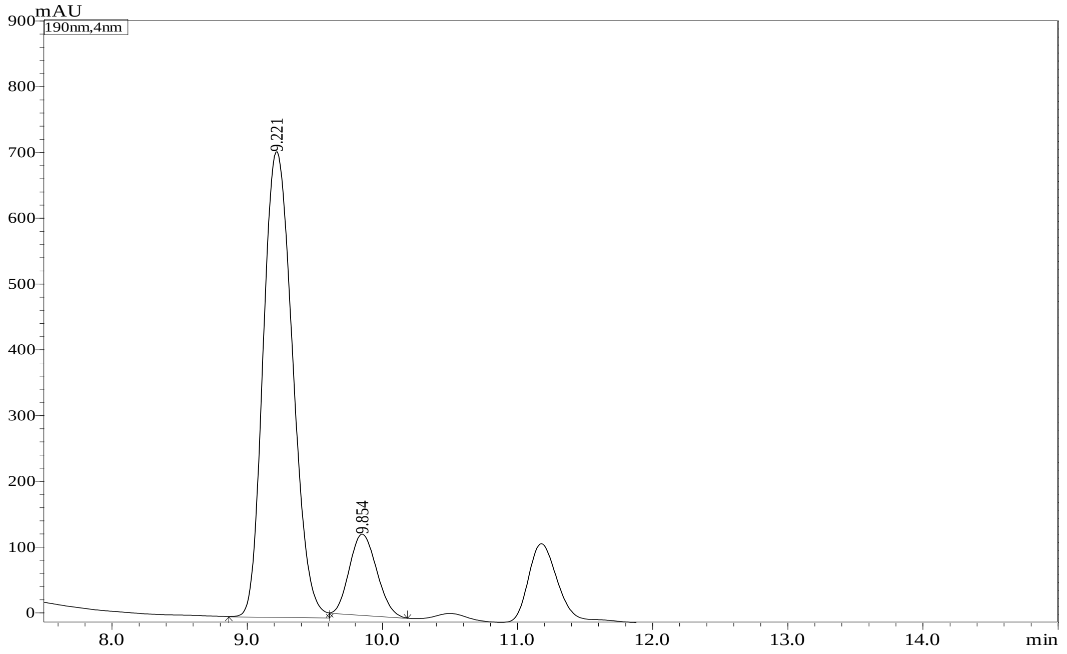

Peak No.	Retention time	Area	Height	Area%
1	9.221	10428624	701095	87.486
2	9.854	1491759	115018	12.514
Total		11920383	816113	100.000

Standard Curve for **3i** is displayed below:

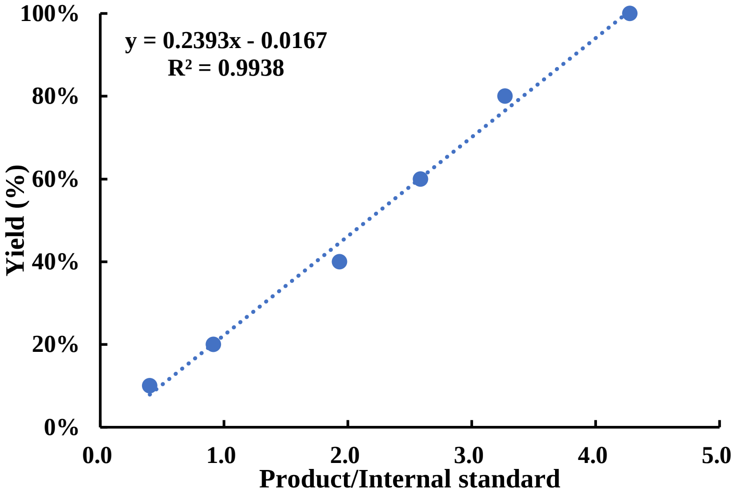

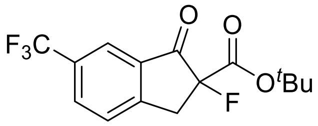

3i

Enantiomeric excess was established by HPLC: IK-3, n-hexane/isopropanol = 90:10, flow rate1mL/min, t major = 5.899 min, t minor = 7.010 min.

HPLC trace of racemate **3i**:

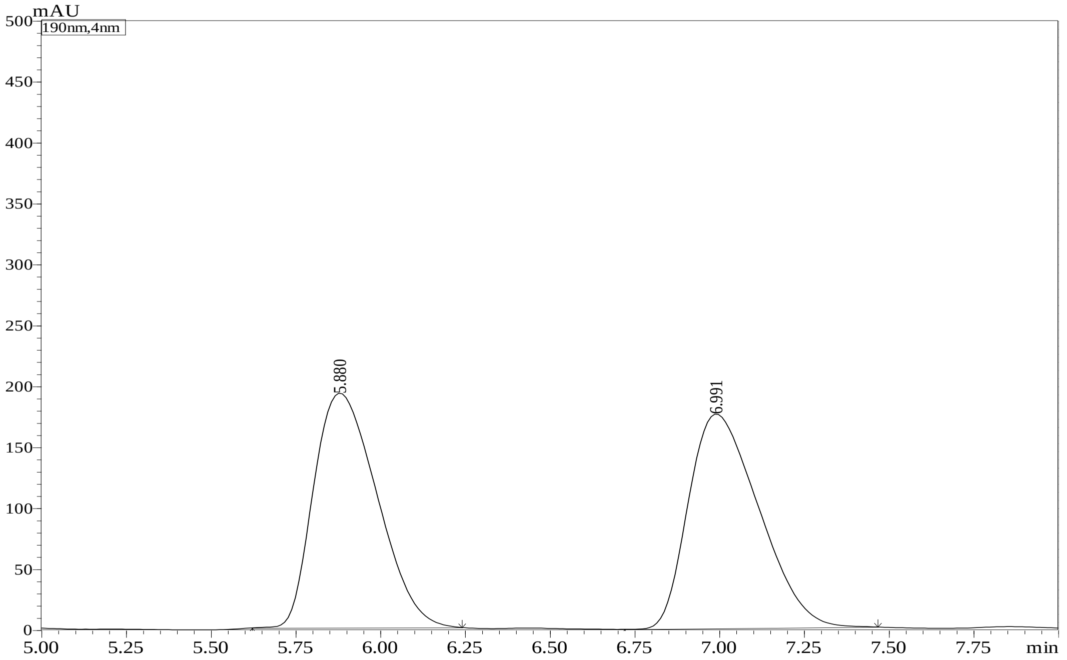

Peak No.	Retention time	Area	Height	Area%
1	5.880	2507708	192470	49.652
2	6.991	2542846	175801	50.348
Total		5050555	368270	100.000

HPLC trace of enantiomerically enriched **3i**:

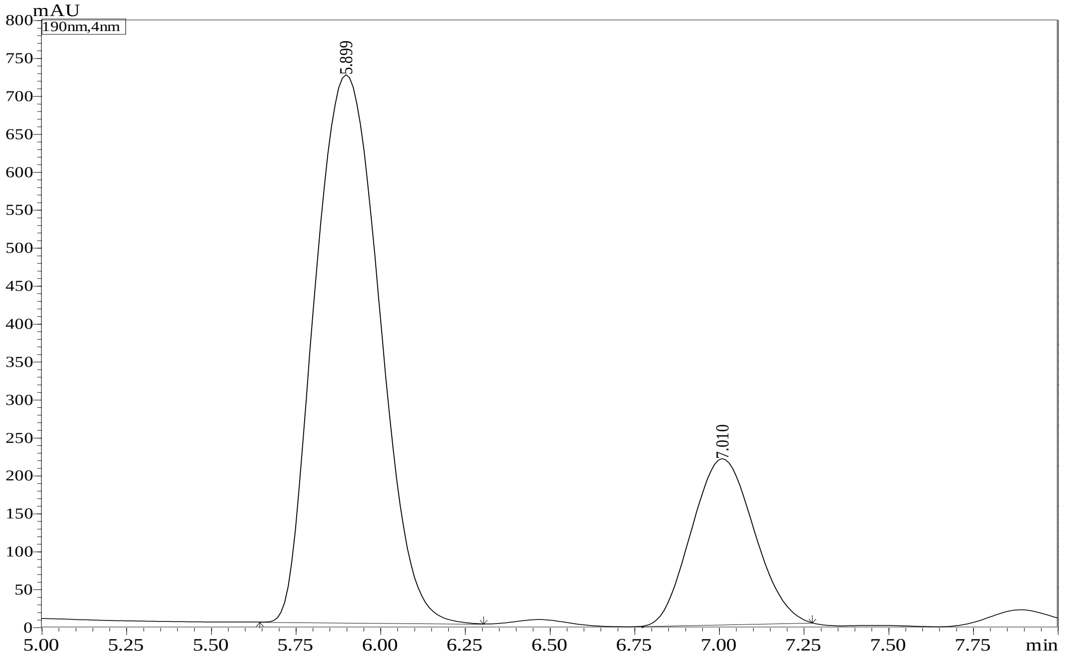

Peak No.	Retention time	Area	Height	Area%
1	5.899	9583295	722132	77.342
2	7.010	2807494	218514	22.658
Total		12390790	940647	100.000

Standard Curve for **3j** is displayed below:

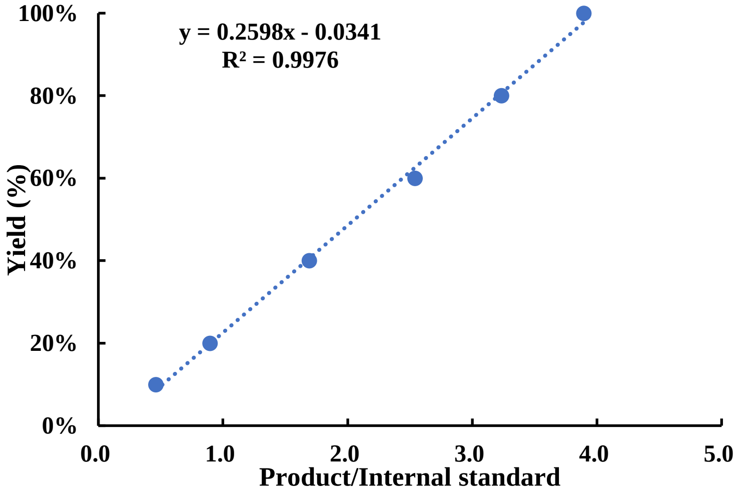

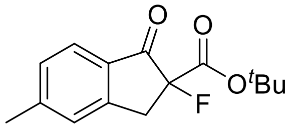

3j

Enantiomeric excess was established by HPLC: IK-3, n-hexane/isopropanol = 95:5, flow rate1mL/min, t major = 12.855 min, t minor = 15.356 min.

HPLC trace of racemate **3j**:

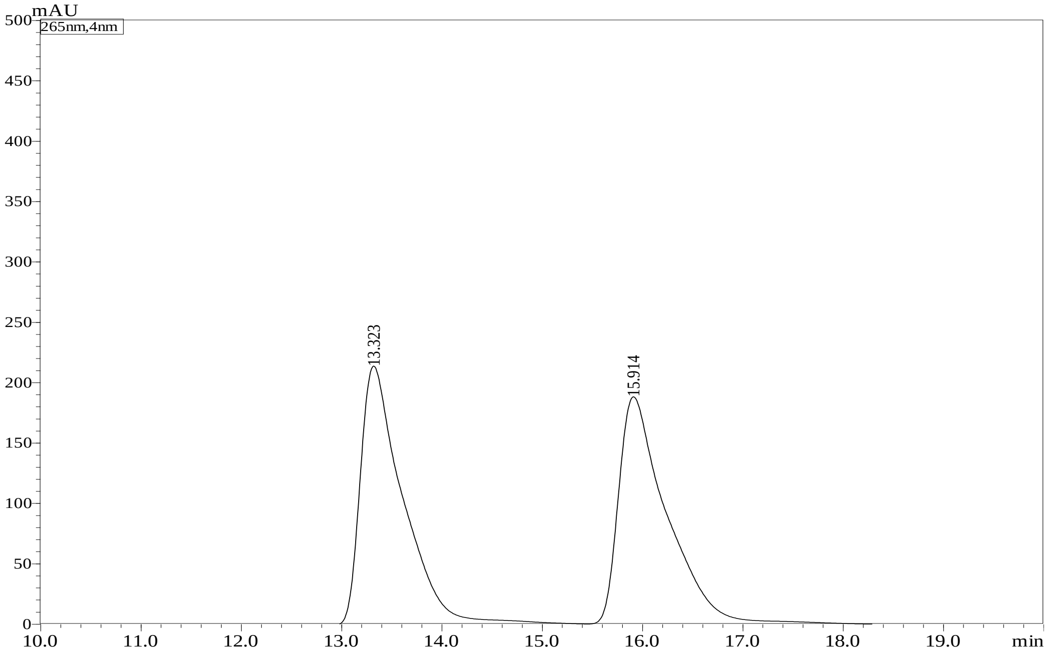

Peak No.	Retention time	Area	Height	Area%
1	13.323	6432456	214804	49.977
2	15.914	6438398	189173	50.023
Total		12870854	403977	100.000

HPLC trace of enantiomerically enriched **3j**:

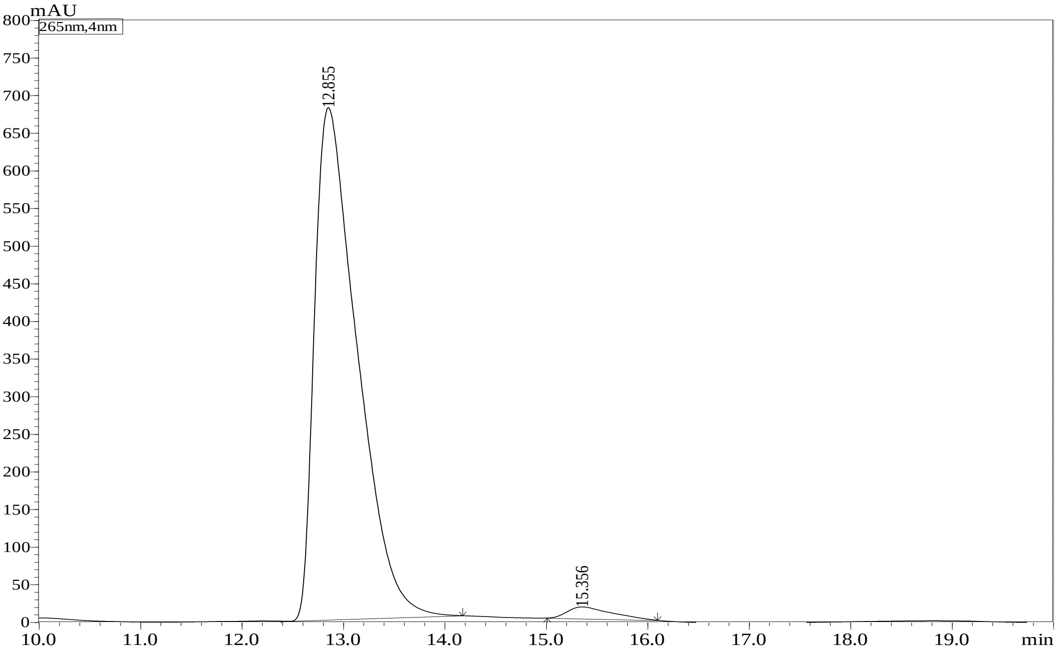

Peak No.	Retention time	Area	Height	Area%
1	12.855	19863526	680537	97.665
2	15.356	474926	15755	2.335
Total		20338453	696291	100.000

Standard Curve for **3k** is displayed below:

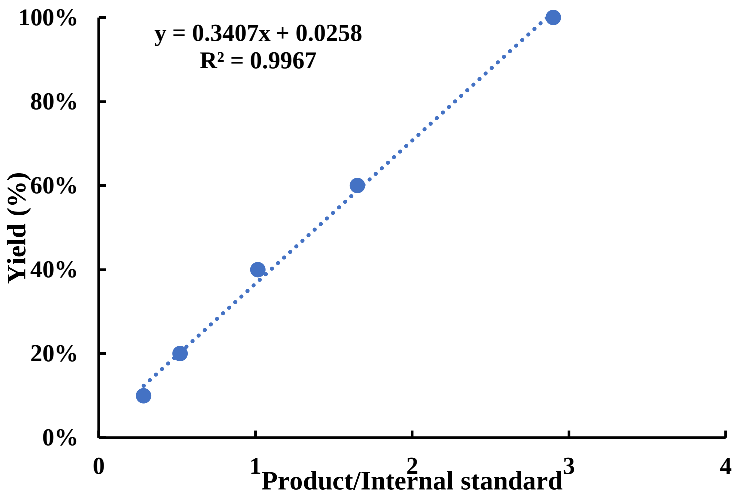

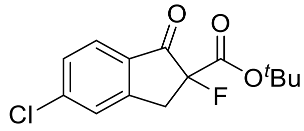

3k

Enantiomeric excess was established by HPLC: IK-3, n-hexane/isopropanol = 90:10, flow rate 1mL/min, t major = 8.050 min, t minor = 9.737 min.

HPLC trace of racemate **3k**:

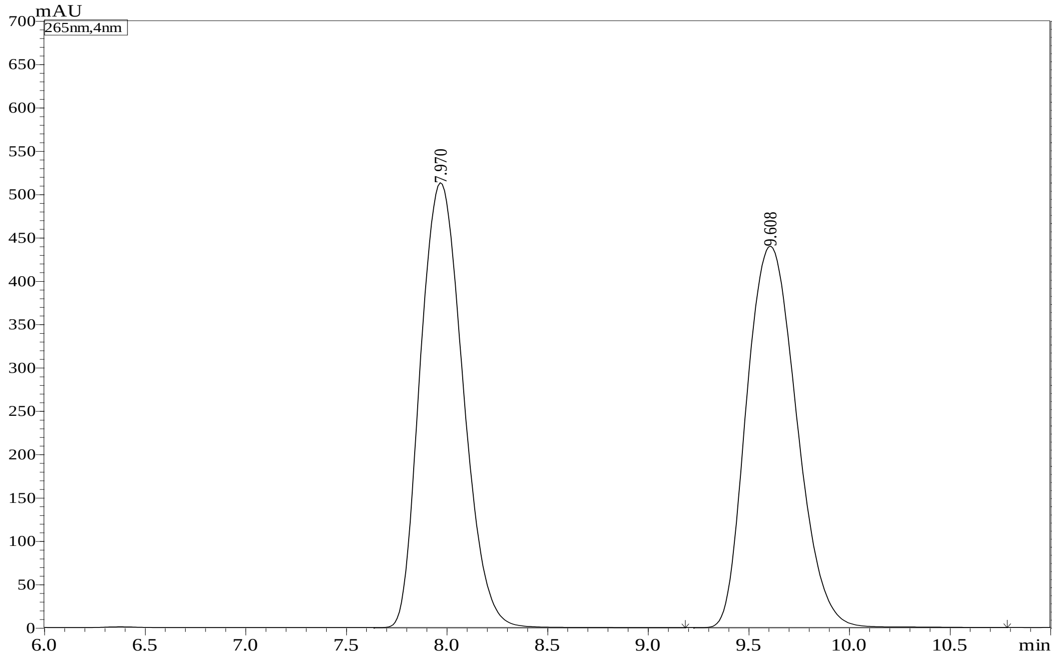

Peak No.	Retention time	Area	Height	Area%
1	7.970	7559140	513086	49.820
2	9.608	7613619	440048	50.180
Total		15172759	953134	100.000

HPLC trace of enantiomerically enriched **3k**:

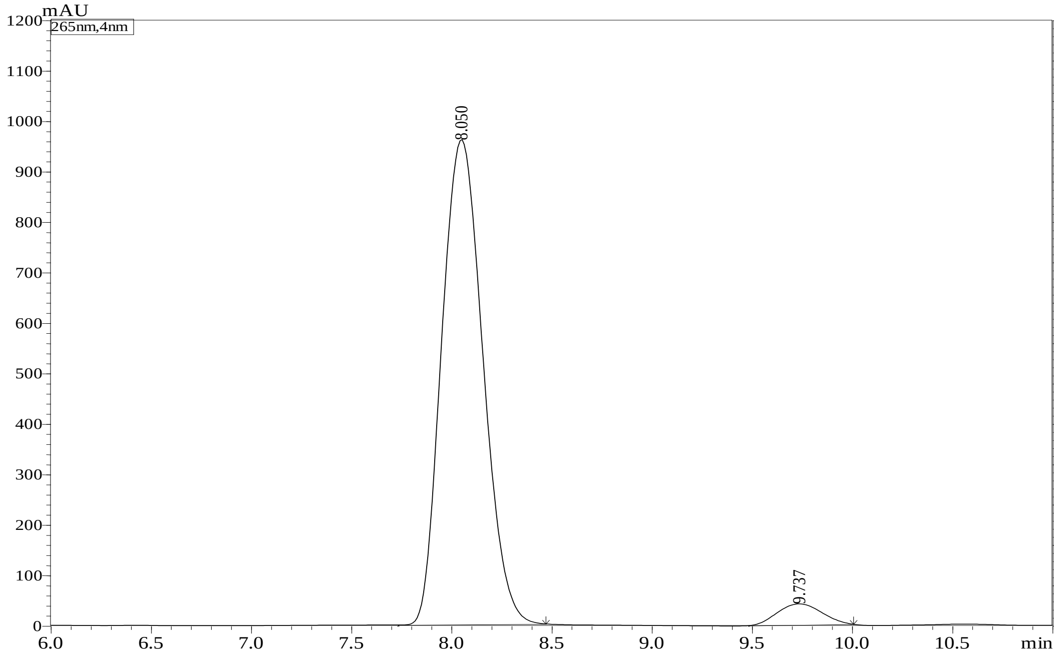

Peak No.	Retention time	Area	Height	Area%
1	8.050	13709175	960792	95.534
2	9.737	640852	41753	4.466
Total		14350027	1002545	100.000

Standard Curve for **3l** is displayed below:

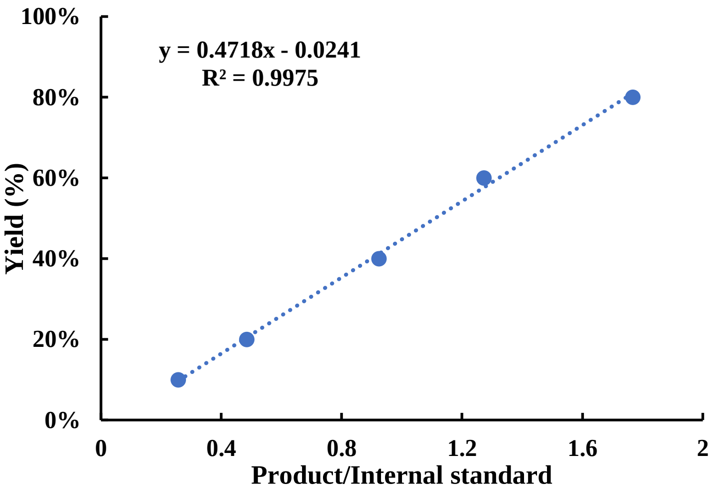

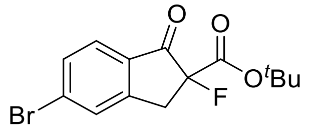

3l

Enantiomeric excess was established by HPLC: OD-H, n-hexane/isopropanol = 98:2, flow rate 1 mL/min, t major = 9.817 min, t minor = 8.749 min.

HPLC trace of racemate **3l**:

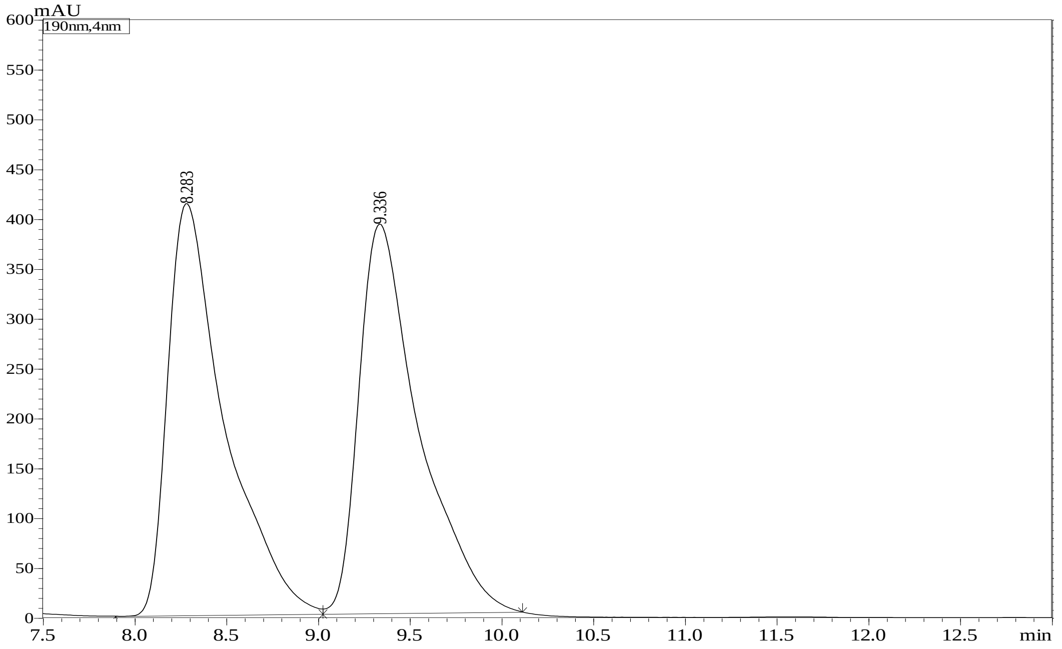

Peak No.	Retention time	Area	Height	Area%
1	8.283	8897604	413058	50.165
2	9.336	8838937	390659	49.835
Total		17736541	803718	100.000

HPLC trace of enantiomerically enriched **3l**:

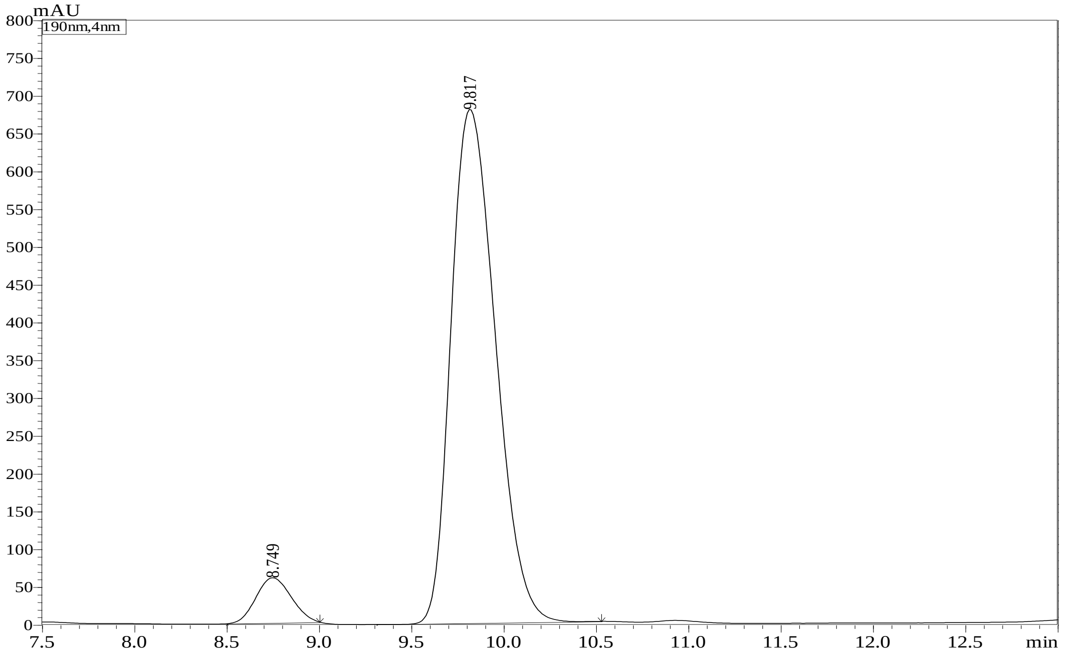

Peak No.	Retention time	Area	Height	Area%
1	8.749	789430	59725	6.492
2	9.817	11370474	680057	93.508
Total		12159904	739782	100.000

Standard Curve for **3m** is displayed below:

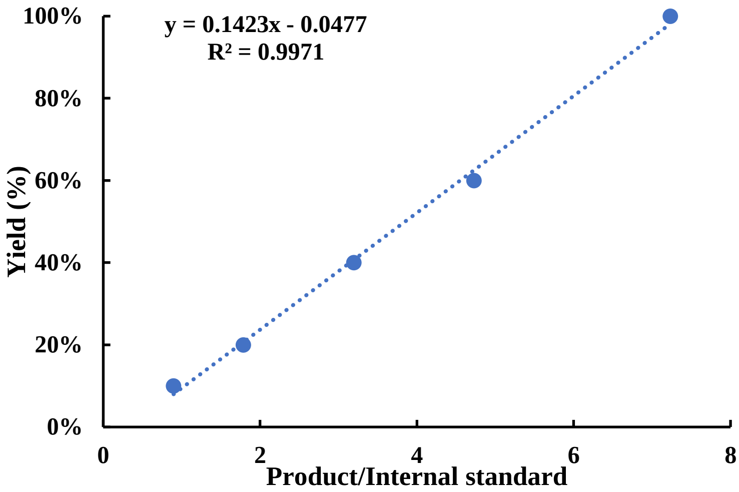

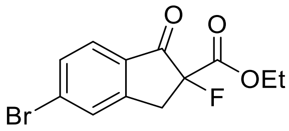

3m

Enantiomeric excess was established by HPLC: AD-H, n-hexane/isopropanol = 98:2, flow rate 0.5 mL/min, t major = 25.073 min, t minor = 28.444 min.

HPLC trace of racemate **3m**:

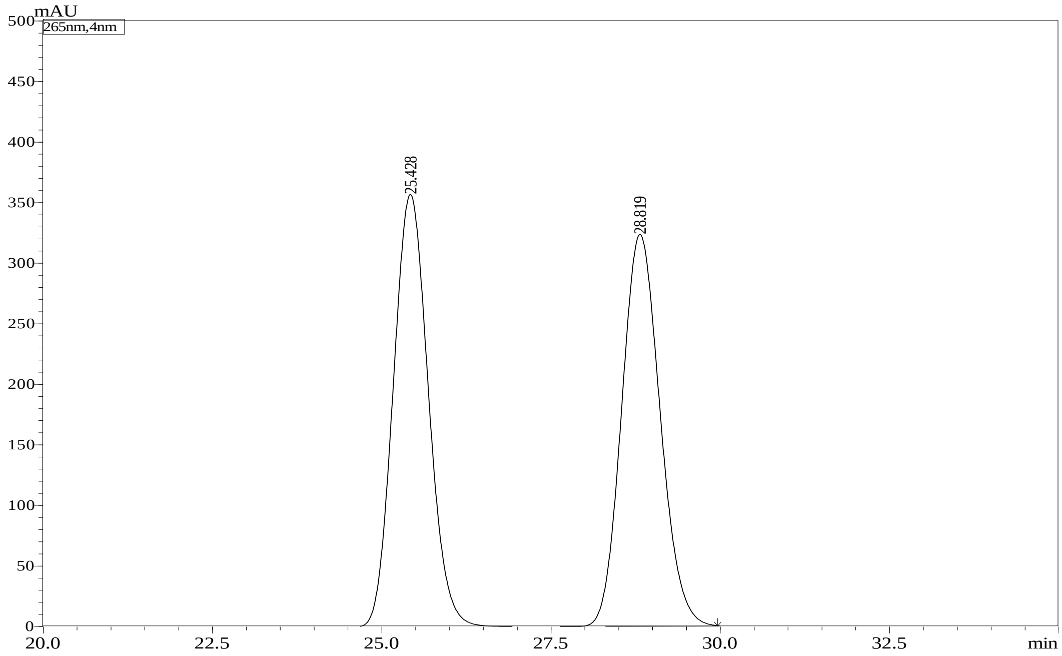

Peak No.	Retention time	Area	Height	Area%
1	25.428	12852199	357115	50.222
2	28.819	12738531	323438	49.778
Total		25590729	680553	100.000

HPLC trace of enantiomerically enriched **3m**:

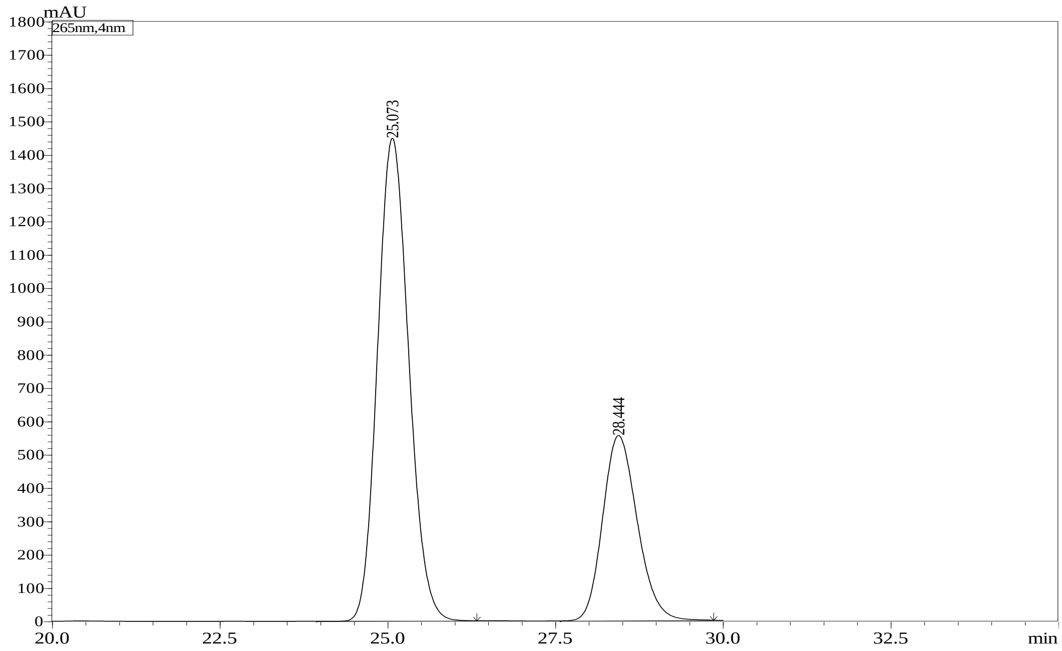

Peak No.	Retention time	Area	Height	Area%
1	25.073	46866481	1448789	70.195
2	28.444	19899606	555244	29.805
Total		66766087	2004032	100.000

Standard Curve for **3n** is displayed below:

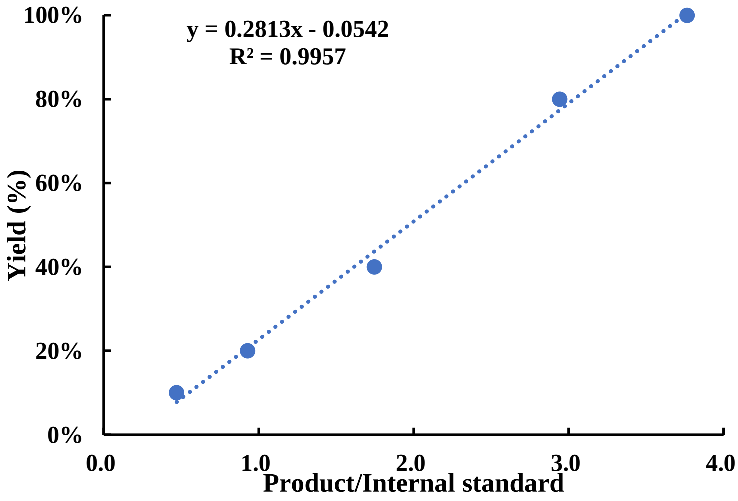

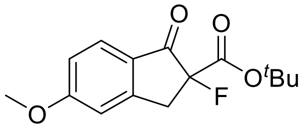

3n

Enantiomeric excess was established by HPLC: IK-3, n-hexane/isopropanol = 90:10, flow rate1mL/min, t major = 15.886 min, t minor = 19.882 min.

HPLC trace of racemate **3n**:

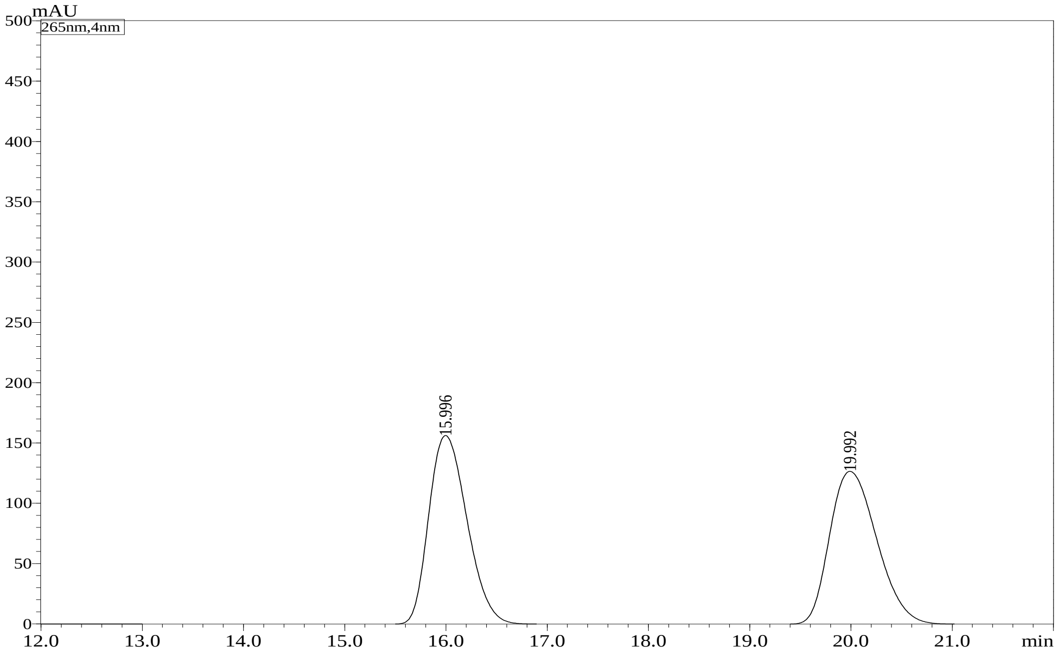

Peak No.	Retention time	Area	Height	Area%
1	15.996	4121256	156452	50.073
2	19.992	4109239	126752	49.927
Total		8230495	283204	100.000

HPLC trace of enantiomerically enriched **3n**:

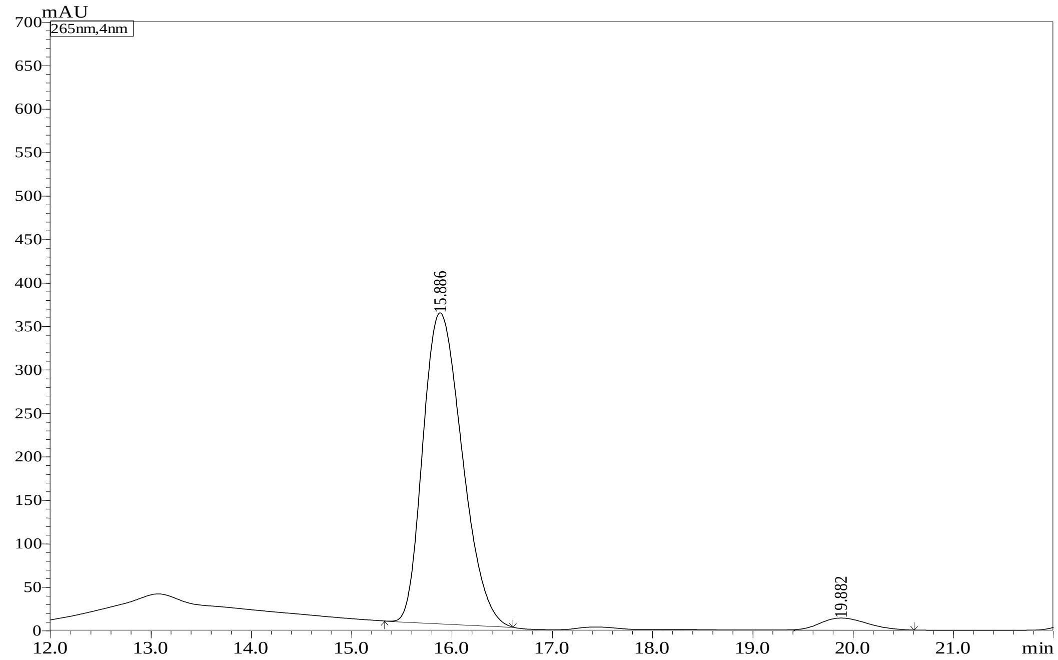

Peak No.	Retention time	Area	Height	Area%
1	15.886	9713725	357485	95.771
2	19.882	428885	13495	4.229
Total		10142610	370980	100.000

Standard Curve for **3o** is displayed below:

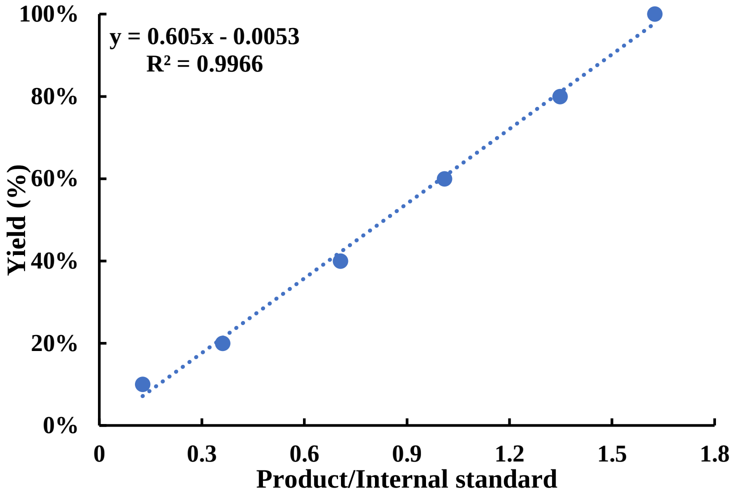

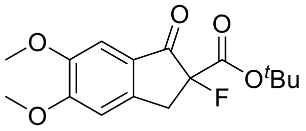

3o

Enantiomeric excess was established by HPLC: AD-H, n-hexane/isopropanol = 85:15, flow rate 0.7 mL/min, t major = 11.063 min, t minor = 12.234 min.

HPLC trace of racemate **3o**:

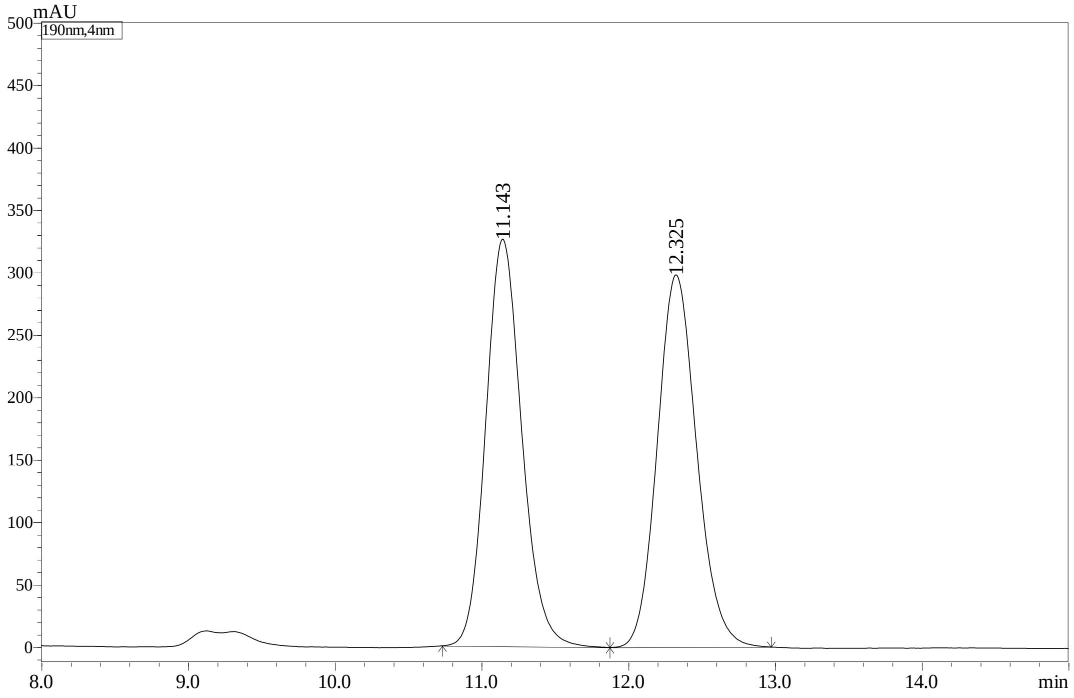

Peak No.	Retention time	Area	Height	Area%
1	11.143	5560972	326108	49.980
2	12.325	5565352	298148	50.020
Total		11126324	624256	100.000

HPLC trace of enantiomerically enriched **3o**:

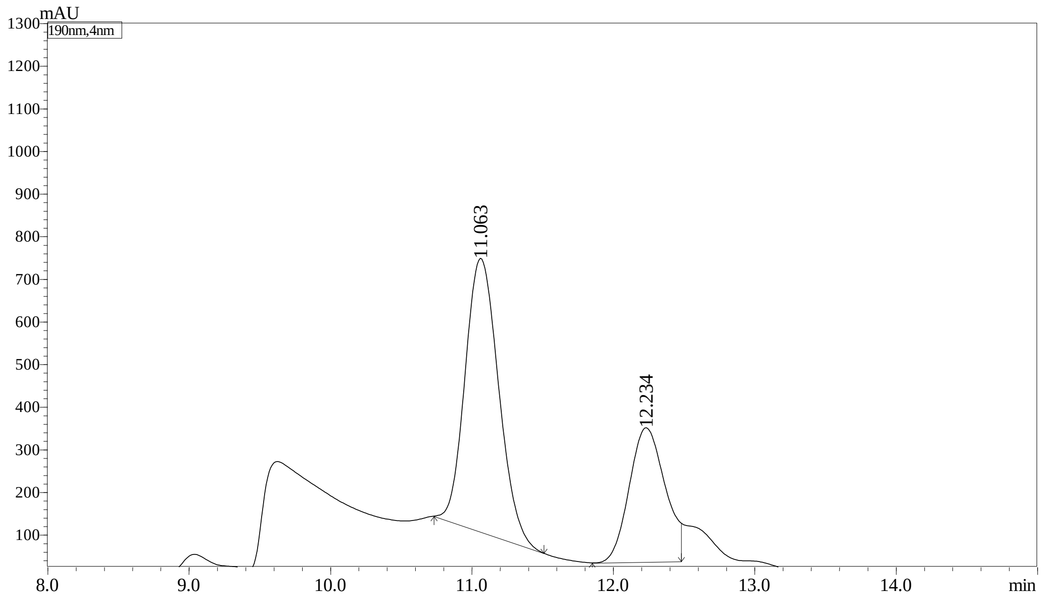

Peak No.	Retention time	Area	Height	Area%
1	11.063	10559115	641844	65.538
2	12.234	5552428	314976	34.462
Total		16111544	956820	100.000

Standard Curve for **3p** is displayed below:

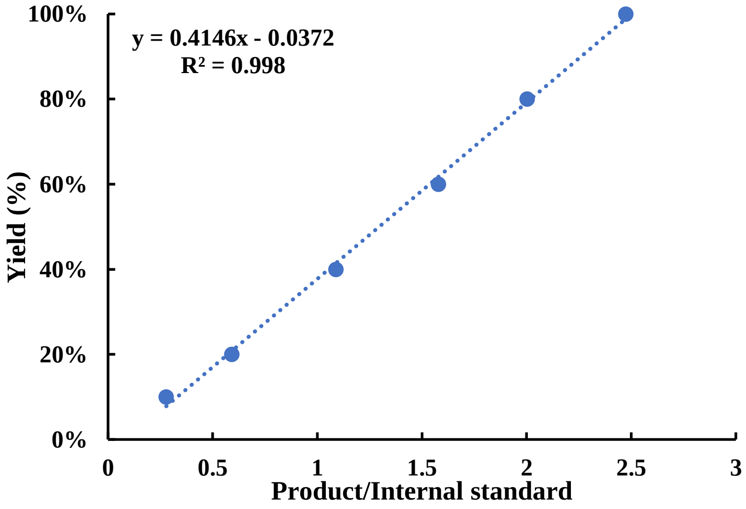

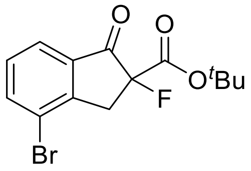

3p

Enantiomeric excess was established by HPLC: AD-H, n-hexane/isopropanol = 99.5:0.5, flow rate 0.5mL/min, t major = 16.353 min, t minor = 26.980 min.

HPLC trace of racemate **3p**:

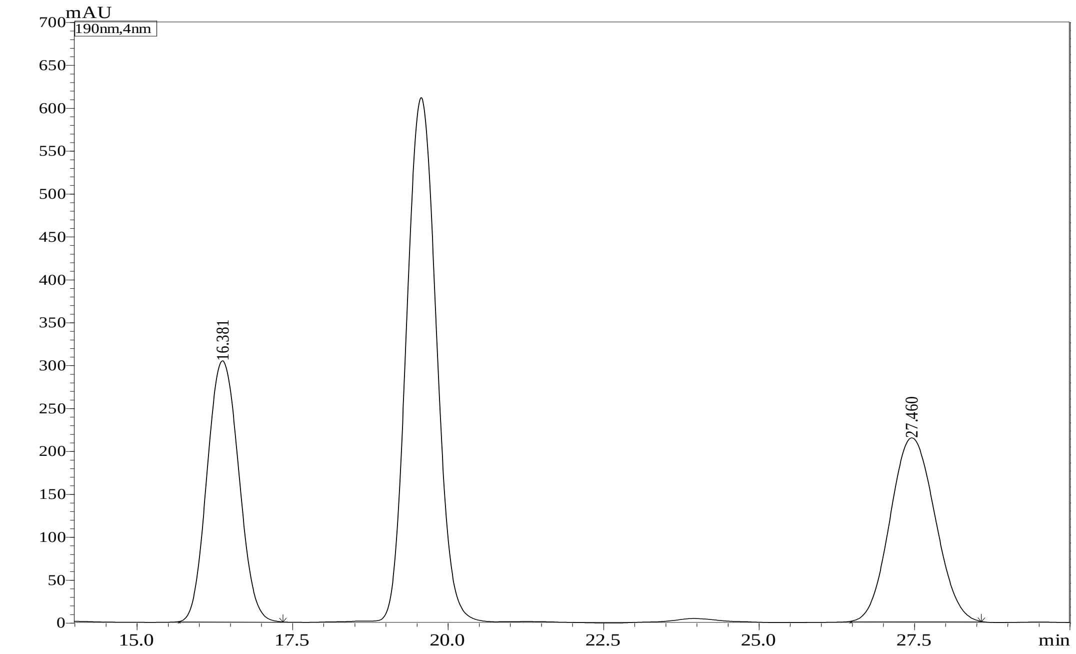

Peak No.	Retention time	Area	Height	Area%
1	16.381	10765544	303982	50.108
2	27.460	10719151	213862	49.892
Total		21484695	517844	100.000

HPLC trace of enantiomerically enriched **3p**:

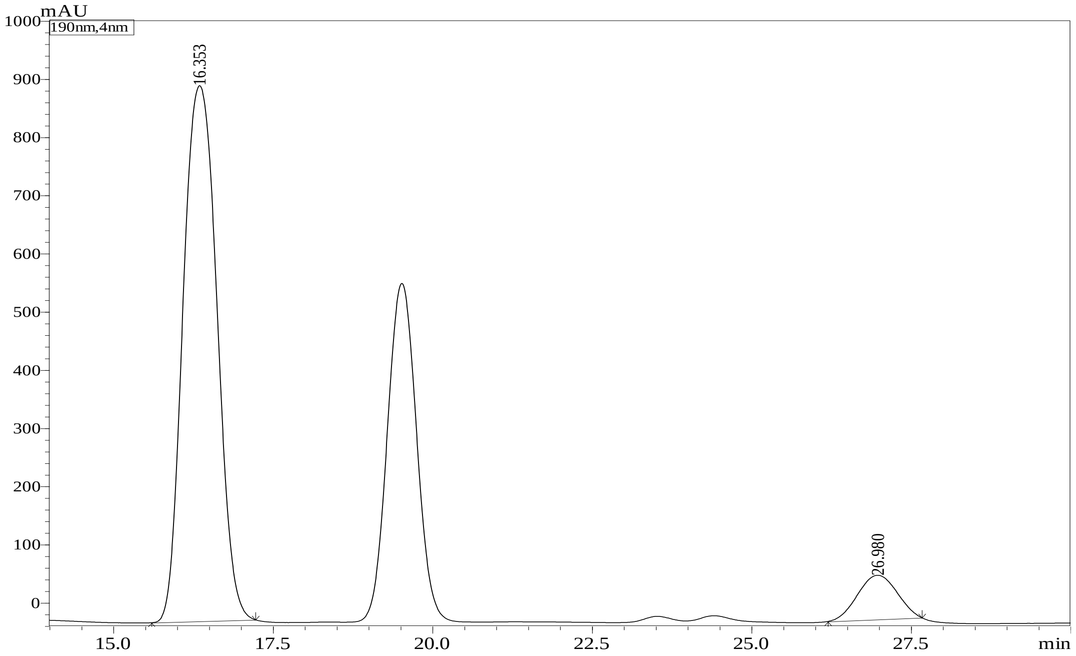

Peak No.	Retention time	Area	Height	Area%
1	16.353	33918191	920554	91.208
2	26.980	3269453	76048	8.792
Total		37187644	996602	100.000

Standard Curve for **3q** is displayed below:

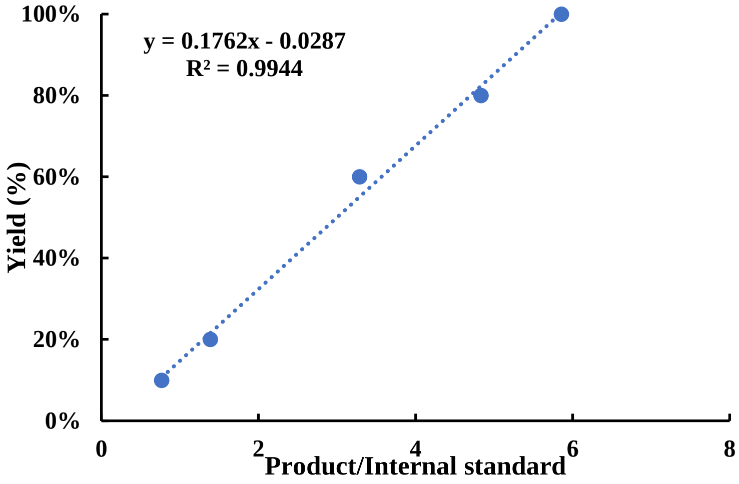

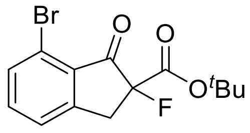

3q

Enantiomeric excess was established by HPLC: AD-H, n-hexane/isopropanol = 97: 3, flow rate 1 mL/min, t major = 10.049 min, t minor = 7.974 min.

HPLC trace of racemate **3q**:

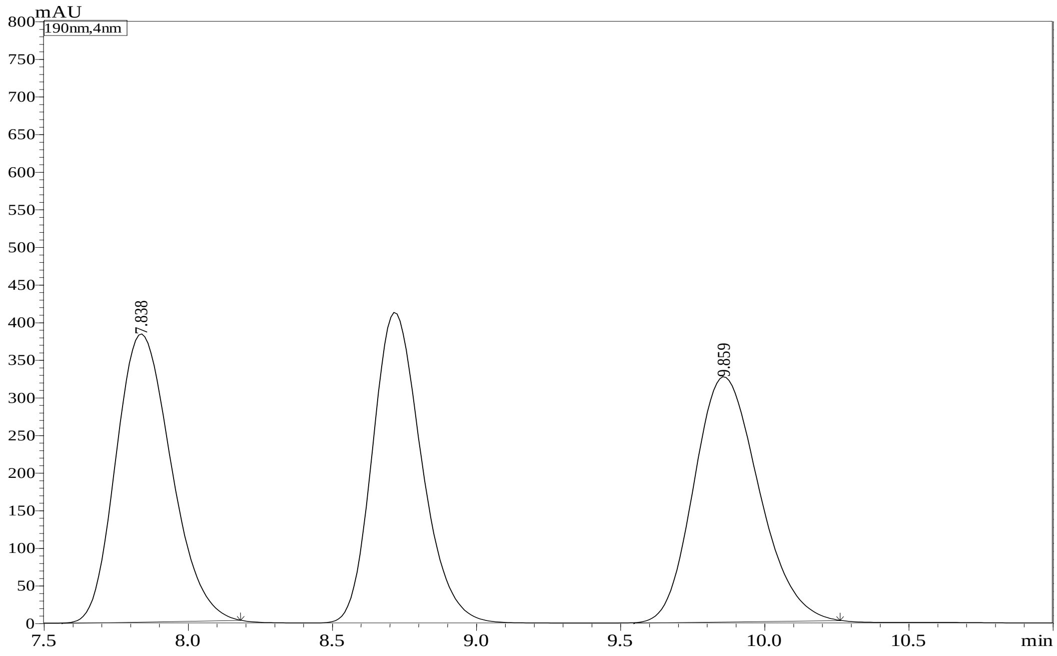

Peak No.	Retention time	Area	Height	Area%
1	7.838	5092506	382759	50.036
2	9.859	5085249	325446	49.964
Total		10177755	708204	100.000

HPLC trace of enantiomerically enriched **3q**:

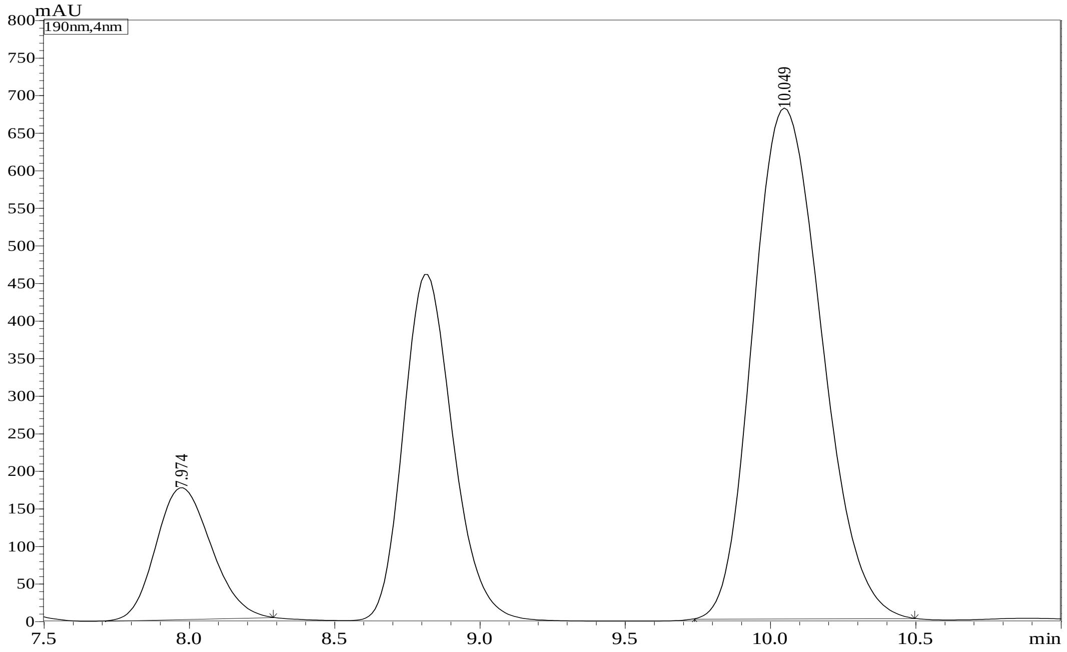

Peak No.	Retention time	Area	Height	Area%
1	7.974	2316498	174969	17.147
2	10.049	11193044	679446	82.853
Total		13509542	854415	100.000

Standard Curve for **3r** is displayed below:

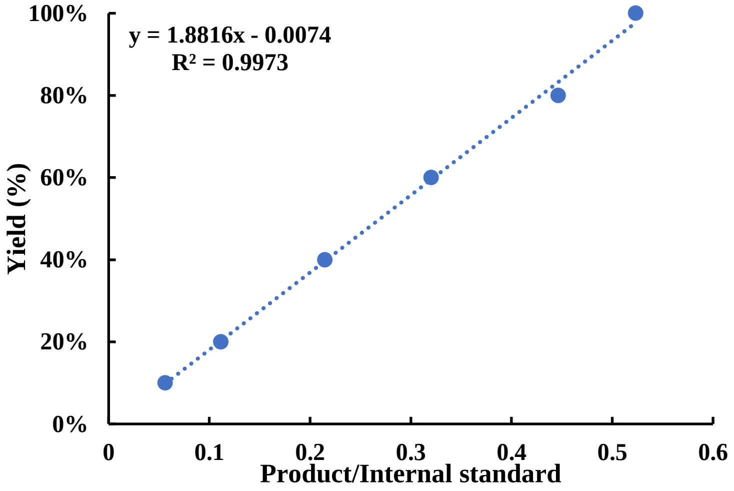

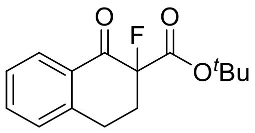

3r

Enantiomeric excess was established by HPLC: IE-3, n-hexane/isopropanol = 90:10, flow rate 1mL/min, t major = 11.884 min, t minor = 13.502 min.

HPLC trace of racemate **3r**:

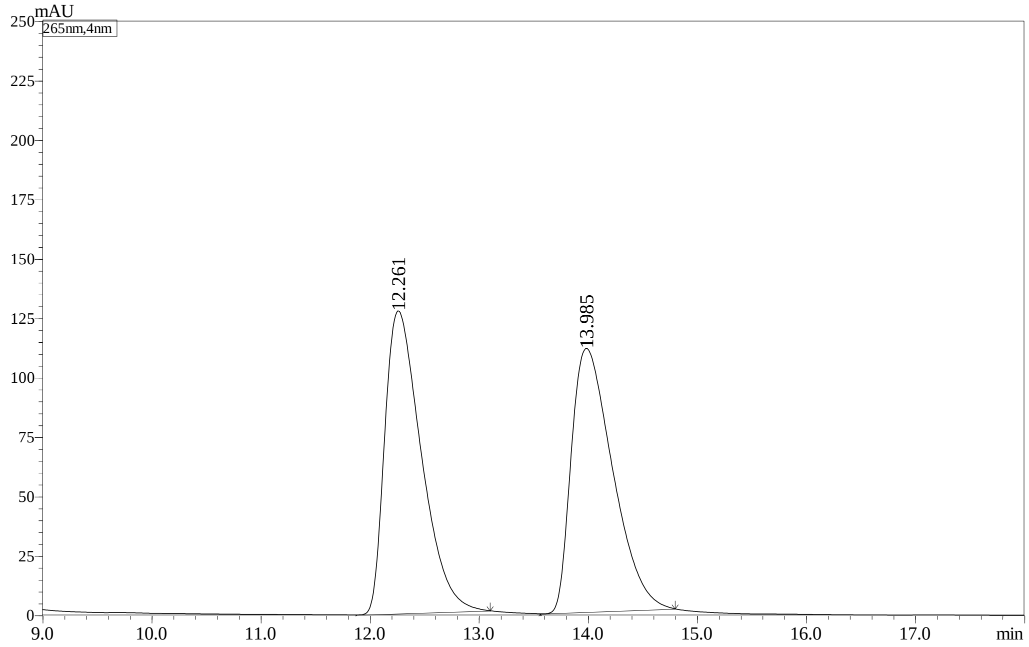

Peak No.	Retention time	Area	Height	Area%
1	12.261	2963722	127368	50.333
2	13.985	2924495	110942	49.667
Total		5888217	238310	100.000

HPLC trace of enantiomerically enriched **3r**:

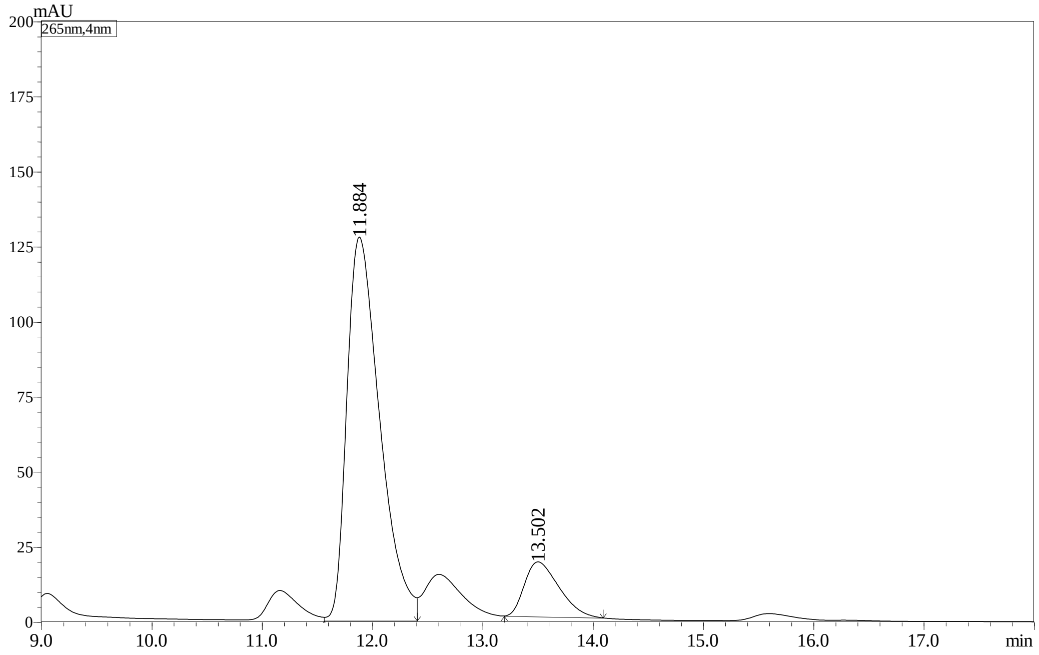

Peak No.	Retention time	Area	Height	Area%
1	11.884	2652649	127814	86.995
2	13.502	396555	18221	13.005
Total		3049204	146035	100.000

Standard Curve for **3s** is displayed below:

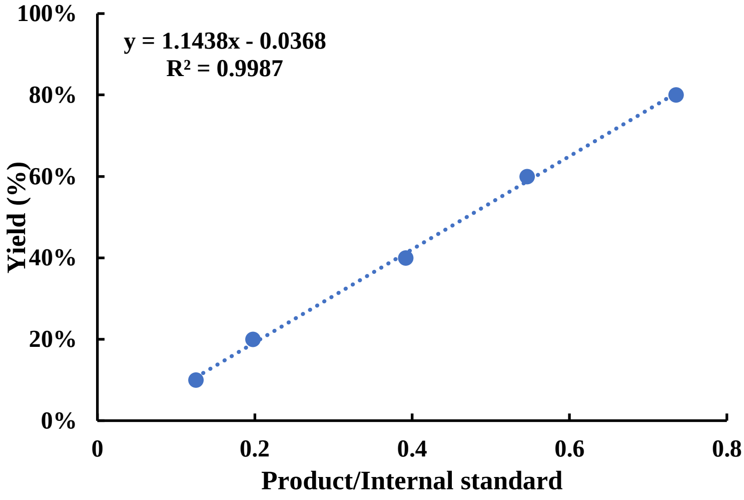

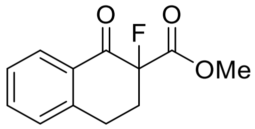

3s

Enantiomeric excess was established by HPLC: IK-3, n-hexane/isopropanol = 95:5, flow rate 1mL/min, t major = 25.092 min, t minor =22.870 min.

HPLC trace of racemate **3s**

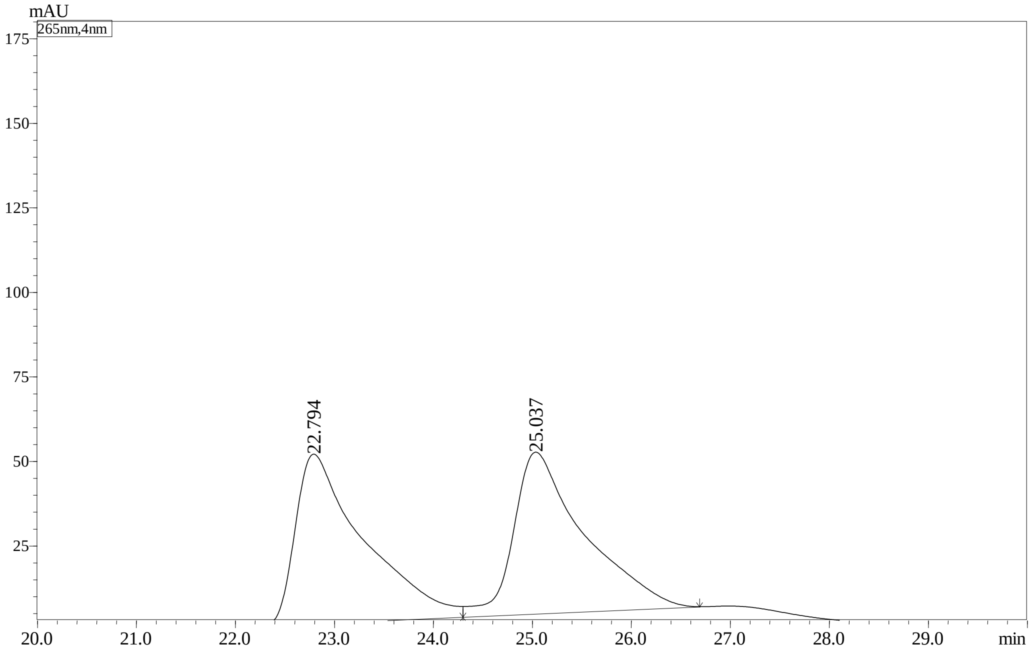

Peak No.	Retention time	Area	Height	Area%
1	22.794	2394079	50083	50.216
2	25.037	2373487	47829	49.784
Total		4767566	97912	100.000

HPLC trace of enantiomerically enriched **3s**:

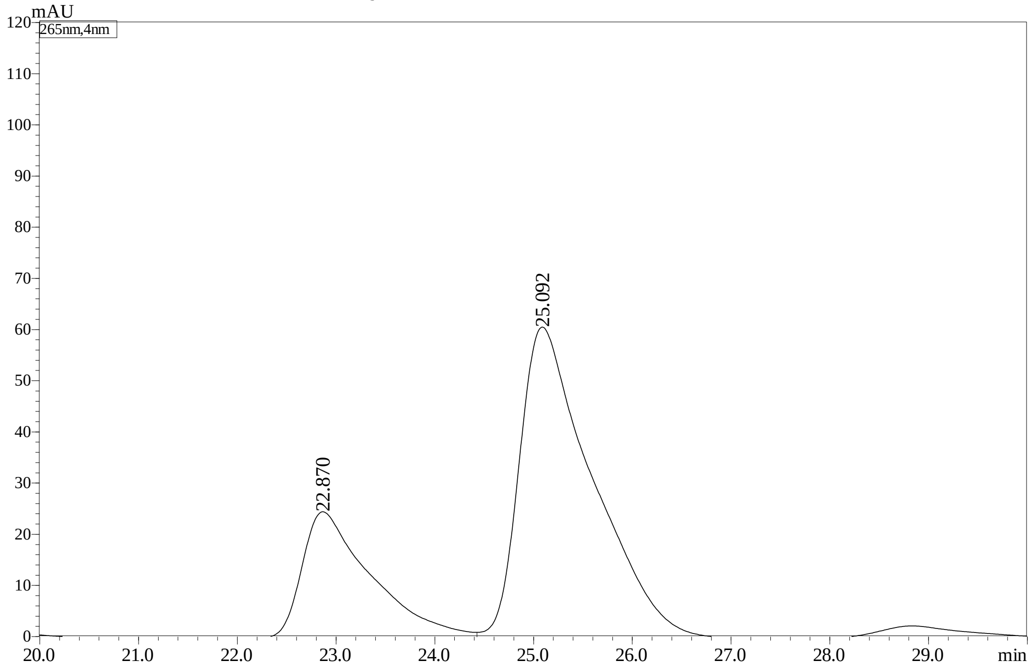

Peak No.	Retention time	Area	Height	Area%
1	22.870	1184996	24557	27.732
2	25.092	3088031	60527	72.268
Total		4273027	85085	100.000

Standard Curve for **3t** is displayed below:

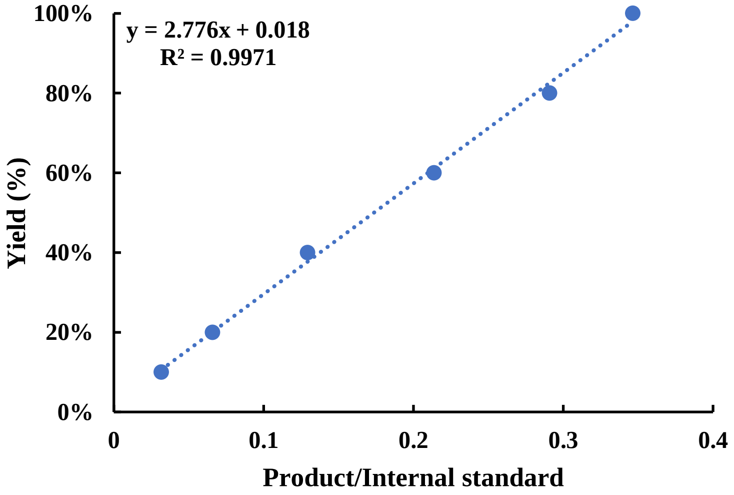

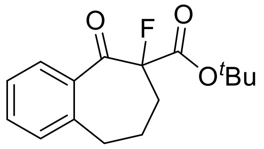

3t

Enantiomeric excess was established by HPLC: IE-3, n-hexane/isopropanol = 98:2, flow rate 1mL/min, t major = 14.785 min, t minor = 16.977 min.

HPLC trace of racemate **3t**:

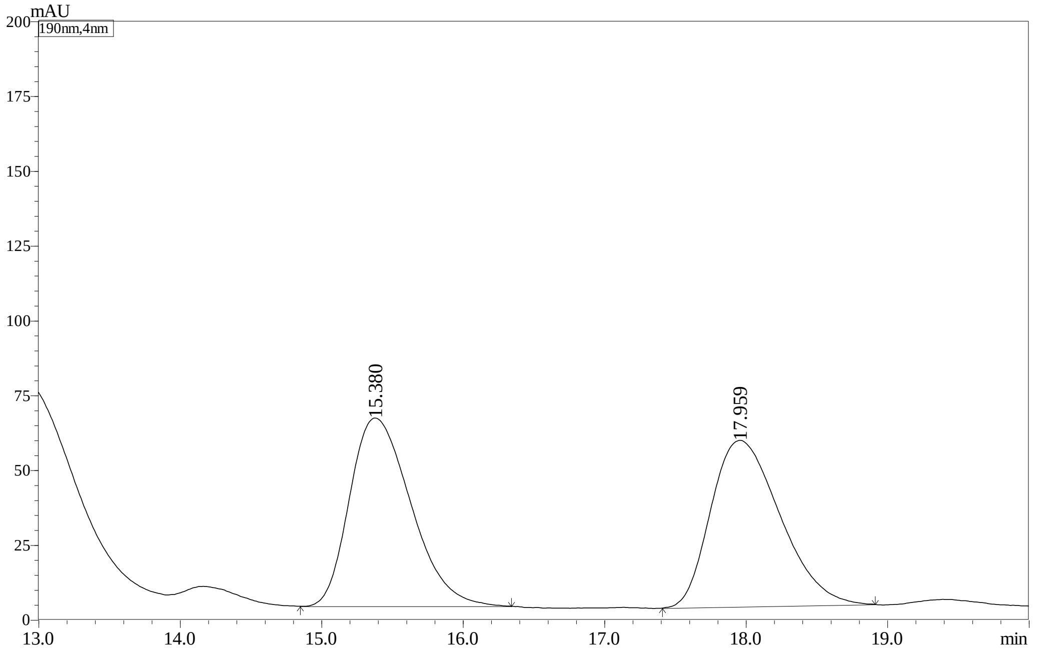

Peak No.	Retention time	Area	Height	Area%
1	15.380	1650460	58739	48.389
2	17.959	1760355	53775	51.611
Total		3410814	112515	100.000

HPLC trace of enantiomerically enriched **3t**:

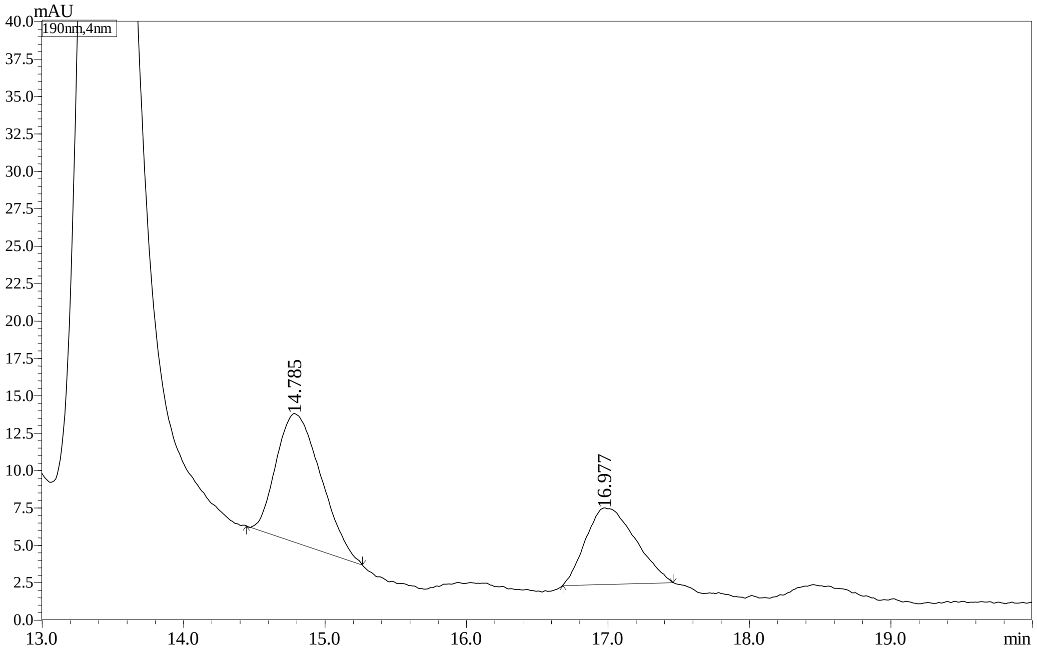

Peak No.	Retention time	Area	Height	Area%
1	14.785	187499	8584	60.258
2	16.977	123662	5093	39.742
Total		311161	13678	100.000

Standard Curve for **3u** is displayed below:

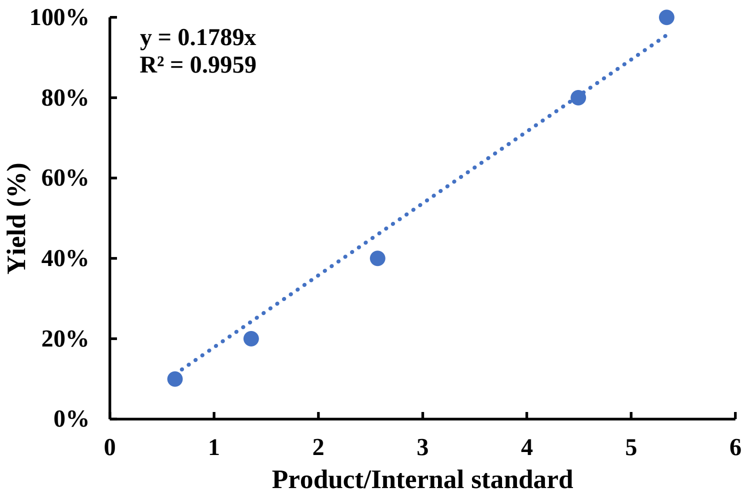

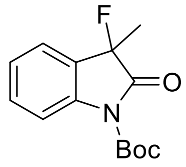

3u

Enantiomeric excess was established by HPLC: OD-H, n-hexane/isopropanol = 99:1, flow rate 0.5 mL/min, t major = 12.629 min, t minor = 10.408 min.

HPLC trace of racemate **3u**:

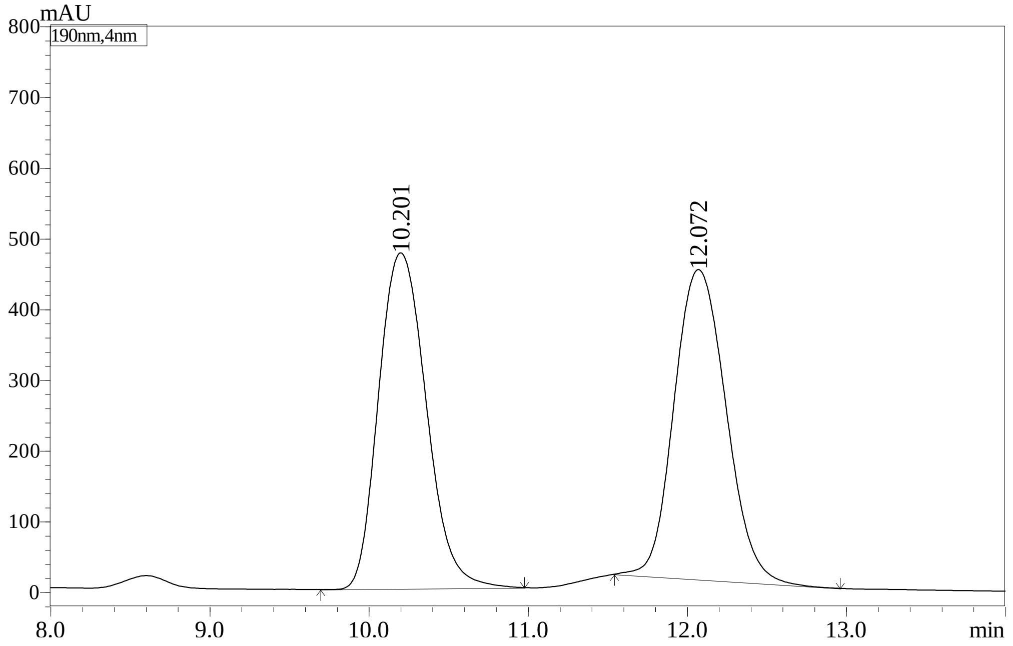

Peak No.	Retention time	Area	Height	Area%
1	10.201	9886830	474866	49.848
2	12.072	9946986	438237	50.152
Total		19833817	913102	100.000

HPLC trace of enantiomerically enriched **3u**:

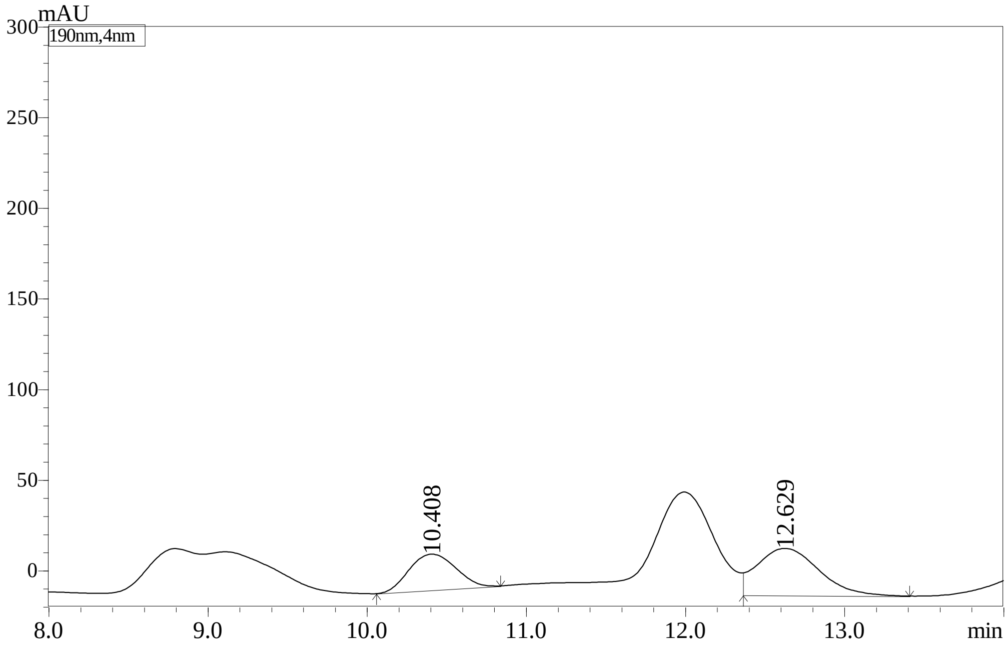

Peak No.	Retention time	Area	Height	Area%
1	10.408	406132	19889	36.798
2	12.629	697537	25859	63.202
Total		1103669	45748	100.000

Standard Curve for **3v** is displayed below:

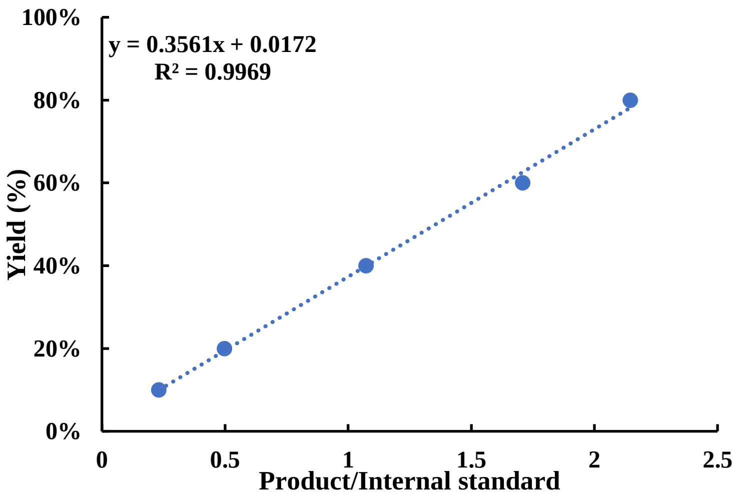

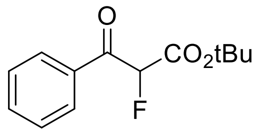

3v

Enantiomeric excess was established by HPLC: AD-H, n-hexane/isopropanol = 99.5:0.5, flow rate 0.4 mL/min, t minor = 22.572 min, t major = 23.924 min.

HPLC trace of racemate **3v**:

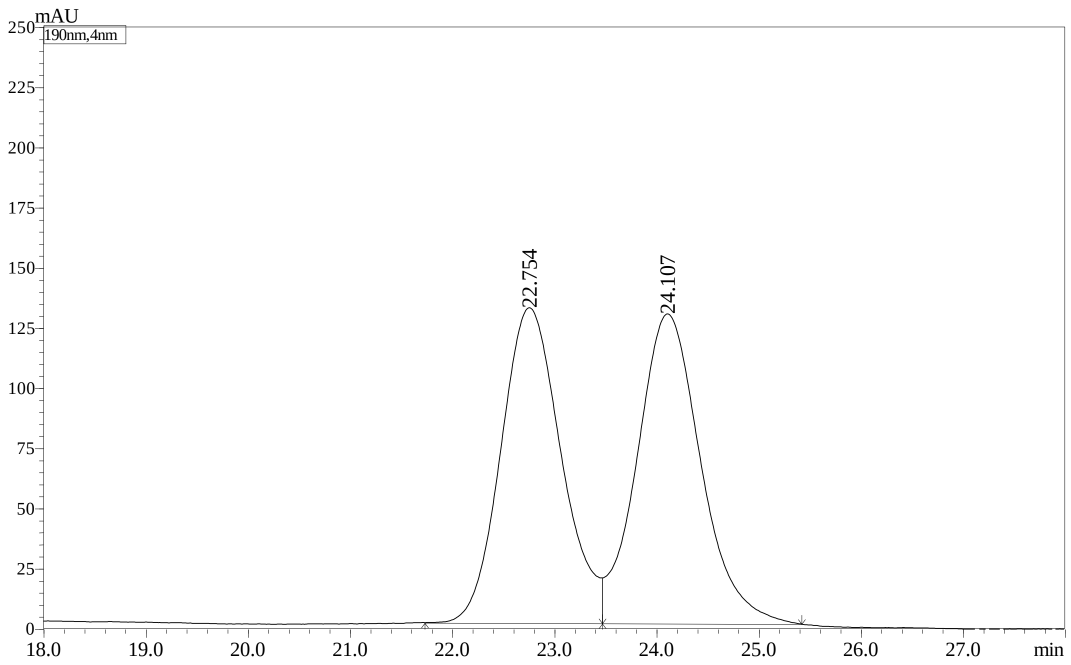

Peak No.	Retention time	Area	Height	Area%
1	22.754	5364347	130989	49.053
2	24.107	5571546	128654	50.947
Total		10935893	259643	100.000

HPLC trace of enantiomerically enriched **3v**:

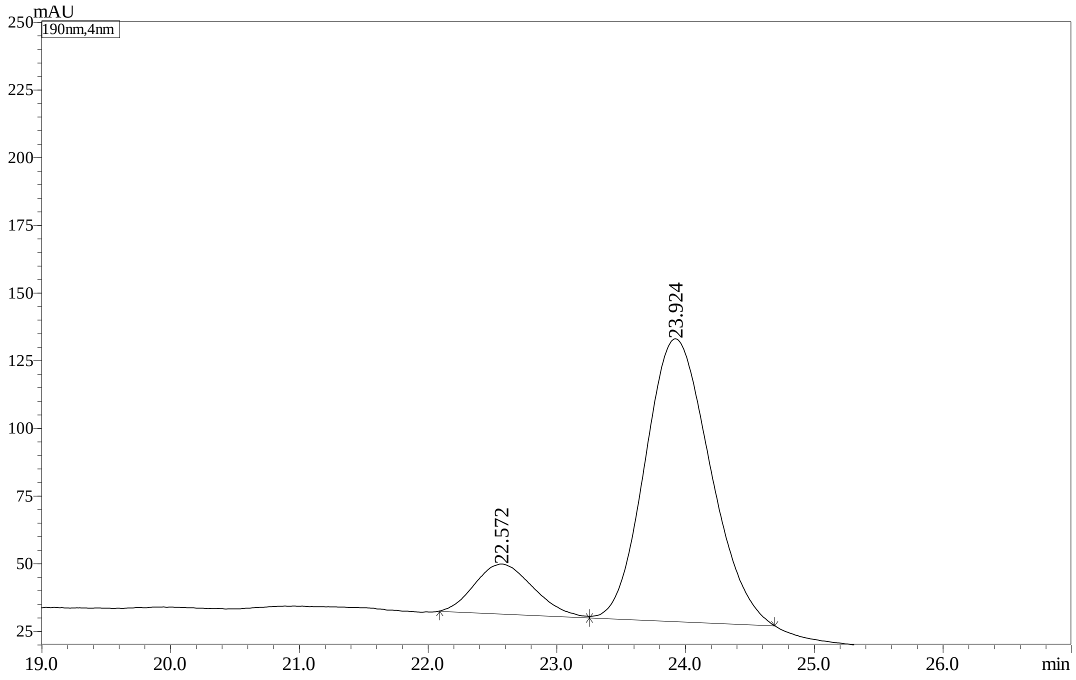

Peak No.	Retention time	Area	Height	Area%
1	22.572	577843	18374	13.635
2	23.924	3660066	104373	86.365
Total		4237090	122747	100.000

9. References

[1] O. Trott, A. J. Olson, *J. Comput. Chem.* **2010**, *31*, 455-461.

[2] G. M. Morris, R. Huey, W. Lindstrom, M. F. Sanner, R. K. Belew, D. S. Goodsell, A. J. Olson, *J. Comput. Chem.* **2009**, *30*, 2785-2791.

[3] D. A. Case, H. M. Aktulga, K. Belfon, D. S. Cerutti, et al. *Journal of Chemical Information and Modeling* **2023**, *63*, 6183-6191.

[4] I. Geibel, J. Christoffers, *European Journal of Organic Chemistry* **2016**, *2016*, 918-920.

[5] A. Dierks, M. Schmidtmann, J. Christoffers, *Chemistry A European Journal* **2019**, *25*, 5451-5462.

[6] G. Magagnano, V. Poirier, F. Romoli, D. Corbisiero, F. Calogero, P. G. Cozzi, A. Gualandi, *European Journal of Organic Chemistry* **2024**, *27*.

[7] X. Ban, Y. Fan, T.-K. Kha, R. Lee, C. W. Kee, Z. Jiang, C.-H. Tan, *CCS Chemistry* **2021**, *3*, 2192-2200.

[8] S. Zuo, Y. Tao, Z. Liu, K. Zhang, L. Zhang, Y. Ning, F.-E. Chen, *Organic Letters* **2023**, *25*, 410-415.

[9] X. Gu, Y. Zhang, Z.-J. Xu, C.-M. Che, *Chemical Communications* **2014**, *50*.

[10] S. G. Hammer, S. Gobleder, F. Naporra, H.-J. Wittmann, S. Elz, M. R. Heinrich, A. Strasser, *Bioorganic & Medicinal Chemistry Letters* **2016**, *26*, 292-300.

[11] L. B. Thomas B. Poulsen, JoseÂ AlemaÂn, Jacob Overgaard, and, K. A. Jùrgensen*, *J. Am. Chem. Soc* **2007**, *129*, 9.

[12] J. Alemán, E. Reyes, B. Richter, J. Overgaard, K. A. Jørgensen, *Chemical Communications* **2007**.

[13] Y.-N. Duan, Z. Zhang, C. Zhang, *Organic Letters* **2016**, *18*, 6176-6179.

[14] Y. N. Duan, L. Q. Cui, L. H. Zuo, C. Zhang, *Chemistry A European Journal* **2015**, *21*, 13052-13057.

[15] X. Wang, Q. Lan, S. Shirakawa, K. Maruoka, *Chem. Commun.* **2010**, *46*, 321-323.

[16] J. Chen, J. Park, S. M. Kirk, H.-C. Chen, X. Li, D. J. Lippincott, B. Melillo, A. B. Smith, *Organic Process Research & Development* **2019**, *23*, 2464-2469.

[17] S. Ghosh, S. Chaudhuri, A. Bisai, *Organic Letters* **2015**, *17*, 1373-1376.

[18] P. A. Peixoto, A. Boulangé, M. Ball, B. Naudin, T. Alle, P. Cosette, P. Karuso, X. Franck, *Journal of the American Chemical Society* **2014**, *136*, 15248-15256.

[19] M. S. a. H. F. Sleiman, *Bioconjugate Chem.,* **2004**, *15*, 4.

[20] L.-Y. Liao, X.-R. Kong, X.-F. Duan, *The Journal of Organic Chemistry* **2014**, *79*, 777-782.

[21] J. E. Jones, J. L. Slack, P. Fang, X. Zhang, V. Subramanian, C. P. Causey, S. A. Coonrod, M. Guo, P. R. Thompson, *ACS Chemical Biology* **2011**, *7*, 160-165.

[22] C. Mairhofer, V. Haider, T. Bögl, M. Waser, *Organic & Biomolecular Chemistry* **2021**, *19*, 162-165.

[23] Q. H. Deng, H. Wadepohl, L. H. Gade, *Chemistry A European Journal* **2011**, *17*, 14922-14928.

[24] J. Peng, D. M. Du, *RSC Adv.* **2014**, *4*, 2061-2067.

[25] Y. Wang, H. Yuan, H. Lu, W.-H. Zheng, *Organic Letters* **2018**, *20*, 2555-2558.

[26] A. Granados, P. Sarró, A. Vallribera, *Molecules* **2019**, *24*.

[27] T. S. Yoshitaka Hamashima, Hisashi Takano, Yuta Shimura,and Mikiko Sodeoka, *J. Am.*

Chem. Soc **2005**, *127*, 10164-10165.
[28] M. A. M. Huarui He, Marc J. P. Leiner, Robert J. Fraatz, and James K. Tusa, *J. Am. Chem. Soc* **2003**, *125*, 1468-1469.
